# The Sixth Brainstorming Research Assembly for Young Neuroscientists (BraYn), Naples, Italy, 27–29 September 2023

**DOI:** 10.3390/neurolint16010011

**Published:** 2024-01-19

**Authors:** Giovanni Ferrara

**Affiliations:** Experimental Neuroscience Laboratory, IRCCS Ospedale Policlinico San Martino, 16132 Genoa, Italy; giovanni.ferrara@hsanmartino.it

**Keywords:** neuroscience, basic research, clinical research, applied research young investigators, scientific society

## Abstract

The BraYn association aims to bolster young neuroscientists’ research endeavors through collaborative support, fundraising assistance, and events promoting knowledge exchange and collaboration across Europe. Central to its mission is the annual BraYn conference, tailored for PhD students, postdocs, junior PIs, neurologists, and clinicians. This gathering champions cooperation, offering talks by key figures, educational workshops, and opportunities for attendees to present their work, compete for grants, and engage in international scientific experiences. The conference, established in 2018, has grown substantially in attendance and industry support and was adapted during the pandemic with virtual editions. The last sixth edition in Naples (27–29 September 2023) attracted over 300 delegates, focusing on peer-to-peer discussions, interdisciplinary collaboration, and interaction with renowned speakers, solidifying its place as a flagship event for Europe’s budding neuroscience researchers.

## 1. Introduction

The BraYn (Brainstorming research assembly for Young neuroscientists) association stands as a beacon for the support and advancement of young neuroscientists at various stages of their careers. Their mission encompasses fostering fresh collaborations across disparate research groups, aiding in fundraising endeavors, orchestrating scientific events that draw in budding researchers from all corners of Europe to exchange insights and expertise, and providing financial backing for research projects and scientific pursuits abroad. Central to their initiatives is the annual BraYn conference, tailored explicitly for young researchers delving into neuroscience. This gathering is a melting pot of PhD students, postdoctoral fellows, junior PIs, neurology residents, young neurologists, and clinicians from diverse European locales. The conference serves as a response to the hurdles encountered by young neuroscientists in their research journeys, offering a unique platform where they can maximize their scientific exploration. Its ethos is simple yet profound: fostering connections, collaborations, and knowledge-sharing. By championing cooperation among diverse research groups, the conference aims to broaden horizons and elevate the quality of research outcomes. Crafted to nurture active participation and interaction among these young minds, the conference offers a remarkably low registration fee, granting attendees access to keynote speeches across various neuroscience disciplines, engaging educational workshops and symposia, opportunities to showcase their work through abstract submissions, and the chance to vie for grants and international experiences.

Since its inception in 2018, the BraYn conference has seen significant growth and adaptation, navigating the challenges posed by the COVID-19 pandemic with both in-person and virtual editions. Accordingly, the conference’s core aims are multifaceted: providing a platform for young neuroscientists to present and discuss their latest laboratory findings, facilitating long-lasting interdisciplinary collaborations, offering direct interaction opportunities with internationally renowned senior speakers, and solidifying its position as an annual flagship event for Europe’s burgeoning neuroscience researchers.

## 2. Conference Sections

The BraYn Conference encompassed a diverse range of sessions, each shedding light on critical facets within neuroscience research. Below is a comprehensive summary highlighting the pivotal discussions and focal points from each session:

### 2.1. Clinical Neuroscience

This session underscored the fusion of neuroscience data with clinical neurology, emphasizing the importance of leveraging research to comprehend neural foundations of nervous system disorders. The emphasis was on soliciting data with clear translational implications and real-world clinical applications, especially in the realm of biomarker utility and novel treatments for neurological diseases. Clinical case presentations elucidating the practical application of new therapies and biomarkers were also encouraged.

### 2.2. Neuroinflammation

Focusing on inflammatory responses within the central nervous system, this session delved into multiple sclerosis (MS), Neuromyelitis Optica Spectrum Disorder (NMOSD), and other impactful CNS inflammatory diseases affecting young adults. The discussions revolved around elucidating pathogenic mechanisms, understanding immune system involvement in CNS autoimmunity, and unraveling genetic and environmental influences in the development of neuroinflammatory diseases, all with a patient-centered approach.

### 2.3. Neuroplasticity and Neurophysiology

This session centered on exploring the adaptability and modification capacities of the nervous system in response to experiences and injuries. Spanning from the cellular to behavioral levels, discussions encompassed various experimental approaches, including molecular and developmental neurobiology, neuropharmacology, electrophysiology, and behavioral analysis across diverse model organisms, including humans.

### 2.4. Neuro-Oncology

The emergent field of neuro-oncology took the spotlight, highlighting investigations into nervous system tumors. The focus was on identifying molecular mechanisms underlying tumor pathogenesis, with an eye towards developing innovative therapeutic interventions for life-threatening conditions like glioma and medulloblastoma.

### 2.5. Neuroimaging

A comprehensive exploration of structural and functional neuroimaging techniques unfolded in this session. Techniques such as MRI, PET, EEG, and others were dissected for their applications in diagnosing and understanding neurological diseases, spanning from intracranial diseases to cognitive psychology research and brain–computer interfaces.

### 2.6. Neurodegeneration

Delving into progressive nervous system damage leading to irreversible neuronal death, this session centered on diseases like Parkinson’s and Alzheimer’s. Discussions encompassed disease progression, symptomatology, and recent advances in understanding these neurodegenerative conditions, along with a broader spectrum of neurodegenerative diseases and their implications.

### 2.7. Epilepsy, Neurodevelopment, and Neurogenetics

The interconnectedness of these fields was evident, emphasizing the dynamic nature of human neurodevelopment and its implications for neurodevelopmental disorders, epilepsy, and psychiatric conditions. The complexities of epilepsy etiology and the pursuit of therapeutic targets received significant attention, showcasing the interdisciplinary efforts driving advancements in the understanding and treatment of these conditions.

## 3. Conclusions

In conclusion, the BraYn association stands as a driving force behind the growth and empowerment of young neuroscientists across Europe. Through its flagship annual conference and multifaceted initiatives, it fosters a culture of collaboration, knowledge exchange, and support among budding researchers in the field of neuroscience. From facilitating peer-to-peer discussions and interdisciplinary collaborations to providing a platform for showcasing cutting-edge research and offering direct interaction with esteemed senior leaders, the BraYn conference serves as a pivotal event, nurturing the next generation of neuroscience pioneers. As it looks ahead to its seventh edition, the association remains committed to elevating the standards of research, broadening horizons, and cementing its status as a cornerstone in the journey of young European neuroscientists. Abstracts submitted by participating scholars can be found in the Supplementary Materials.

## Data Availability

The datasets utilized and/or examined in the present abstract collection study can be obtained from the first author of each abstract. Author on reasonable request.

## Supplementary Materials

The following supporting information can be downloaded at: https://www.mdpi.com/article/10.3390/neurolint16010011/s1. Abstracts submitted by participating scholars can be found in the Supplementary Materials.

