# Supplementary material for: The Sixth Brainstorming Research Assembly for Young Neuroscientists (BraYn), Naples, Italy, 27–29 September 2023
Source: Neurol Int. 2024 Jan 19;16(1):186–8. doi: 10.3390/neurolint16010011 (PMC10892396; doi:10.3390/neurolint16010011)
Supplement: Supplementary file 1 [file neurolint-16-00011-s001.zip › neurolint-2813205-supplementary.pdf]

A Blood Brain Barrier (BBB) Model to Test Novel Therapeutic Strategies for GLUT-1 Deficiency Syndrome

Simona Baldassari (1) - Valentina Castagnola (2) - Serena Cappato (1) - Ilaria Musante (1) - Renata Bocciardi (1,3) - Paolo Scudieri (1,3) - Fabio Benfenati (2) - Federico Zara (1,3)

Unit of Medical Genetics, IRCCS G. Gaslini, Genova, Italy (1) - Center for Synaptic Neuroscience and Technology, Istituto Italiano di Tecnologia, Genova, Italy (2) - Department of Neurosciences, Rehabilitation, Ophthalmology, Genetics, Maternal and Child Health (DINOGMI), University of Genova, Genova, Italy (3)

Glucose transporter type 1 (GLUT1) is a critical protein allowing glucose efflux to the brain through the Blood-Brain-Barrier (BBB). Monoallelic or bi-allelic mutations in the GLUT1 encoding gene - *SLC2A1* - result in GLUT1-deficiency (GLUT1DS) featuring intractable seizures, intellectual disability, ataxia, and dystonia.
Pathogenic mechanisms are still unclear and specific therapeutic approaches lacking due to the difficulty to obtain appropriate human-derived *in vitro* models.
In this study we aimed to generate a well-established *in vitro* Transwell model of BBB with brain endothelial cells derived from controls and GLUT1DS patients's induced pluripotent stem cells (iPSCs). We selected two patients carrying different mutations and showing a severe (p.Leu124Trpfs*12) and a milder (p.Arg400Cys) GLUT1-deficiency phenotype. We characterized the BBB model with standard tests of BBB functionality, including transendothelial electrical resistance (TEER), GLUT1 expression, immunocytochemistry for endothelial and tight junction markers and paracellular transport across the barrier. The results indicated a different rate of BBB differentiation between the severe and the milder patients' cells with respect to the controls, probably due to the drastic impairment in the nutritional molecules uptake. To improve the BBB model, we are moving from a static to dynamic culture system ensuring optimal microenvironment conditions and mimicking the in vivo physiology.
A standardized BBB model could be used to test novel therapeutical approaches aimed at enhancing BBB glucose permeability. In this regard, we will initiate the search of genetic (*e.g.* by CRISPRa technology) and pharmacological tools able to increase the expression of *SLC2A1* at the transcriptional and translational levels.

Nanostructured materials for the healing of peripheral nervous system (PNS) pathologies

Luca Scaccini (1) - Antonella Battisti (2) - Mariacristina Gagliardi (2) - Ambra Del Grosso (1) - Miriam De Sarlo (2) - Laura Colagiorgio (2) - Sara Carpi (2) - Marco Cecchini (2) - Ilaria Tonazzini (2)

Scuola Normale Superiore, NEST - National Enterprise for nanoScience and nanoTechnology, Pisa, Italy (1) - Istituto Nanoscienze (NANO) - CNR, NEST - National Enterprise for nanoScience and nanoTechnology, Pisa, Italy (2)

Peripheral neuropathies are a condition in which peripheral nerves are damaged. This condition affects millions of people every year worldwide and can be caused by external trauma, or by pathologies that impact peripheral nervous system components. Biocompatible scaffolds are emerging as important tools to promote nerve regeneration in case of resection. However, when there is no physical damage of the nerve an effective drug delivery system is still lacking. To address these issues, we are working on two different approaches. We already demonstrated that nano/micro-grooved substrates are capable to direct neuronal and glial cell differentiation, polarization, and migration. We developed microstructured scaffolds, with specific directional topographies and tuneable stiffness, for peripheral nerve regeneration: our scaffolds, made of biodegradable and soft materials (compliant with nerve mechanics), were tested in vitro with neuronal and glial cell models. Moreover, the restricted permeability of nerves, due to the presence of the blood-nerve barrier (BNB), makes difficult to transport drugs into their structure. Polymeric nanoparticles are under investigation for their ability to pass biological barriers (such as the blood-brain barrier). We developed polymeric nanoparticles (NPs) functionalized with peripheral nerve targeting molecules, procaine and a peptide, NP41. We tested their biocompatibility and internalization capability in vitro in neural cell models. These NPs can be further loaded with drugs of interest. These two strategies can be either used as stand-alone systems or can be further combined to create innovative devices (e.g. scaffolds functionalized/enriched with NPs loaded with compounds of interest). Such devices would be able to provide both a physical support for regeneration, and a controlled release of drugs for treating a wide variety of pathological conditions impacting PNS (e.g. nerve trauma, resections, neurodegenerative disorders).

Mechanisms of synaptic dysfunction in the Angelman syndrome

Federica Baronchelli [1] - Martina Biagioni [2] - Martina Di Nunzio [2] - Marco Erreni [2] - Alessandra Folci [3] - Matteo Fossati [3]

CNR Neuroscience Institute, Humanitas University, Rozzano, Italy (1) - Humanitas Research Hospital, Neuroscience, Rozzano, Italy (2) - CNR Neuroscience Institute, Humanitas Research Hospital, Rozzano, Italy (3)

The Angelman Syndrome (AS) is a neurodevelopmental disorder caused by the loss of maternally expressed *UBE3A* gene, which encodes an E3 ubiquitin ligase. Although considerable efforts have been put to dissect UBE3A function in the brain, the pathogenic mechanisms remain largely unknown and effective treatments are not available yet. Being an E3 ubiquitin ligase, defective ubiquitination is thought to be a primary mechanism underlying synaptic dysfunction in AS. However, only a few substrates of UBE3A have been shown to be relevant to AS pathogenesis and recent reports suggest that UBE3A might play a global regulatory role instead of acting through a specific neuronal pathway. Increasing evidence indicates a tight functional interplay between ubiquitination and sumoylation, an ubiquitin-related PTM consisting in the covalent conjugation of Small Ubiquitin-like MOdifier (SUMO) proteins to target proteins. In the brain the SUMO machinery finely modulates synaptic and extrasynaptic pathways that are fundamental to neuronal circuit formation and function. In this project, we evaluate the impact of *UBE3A* loss on synaptic development and test the hypothesis that alterations of the functional cross-talk between ubiquitination and sumoylation might contribute to AS pathogenesis. To explore the possibility that an unbalanced sumoylation is at the basis of AS, we are currently evaluating sumoylation of nuclear and cytosolic fractions obtained from cortices of AS and wild type mice throughout neurodevelopment. Strikingly, preliminary data obtained in the laboratory indicate that both SUMO1 and SUMO2/3 conjugation is impaired at different developmental stages in the nucleus and in the cytosol of AS cortices. These data might unveil a novel pathogenic mechanism of AS and provide the rationale to develop new therapeutic strategies to treat AS patients.

Immunoevasive Phenotype of Glioma Cells: Hindering CD8 Lymphocyte Cytotoxicity through CD4 Lymphocyte Modulation

Irene Appolloni [(1)] - Giorgia Guccione [(1)] - Francesca Piaggio [(2)] - Chiara Riviera [(1)] - Daniela Marubbi [(1,2)] - Davide Ceresa [(2)] - Fabrizio Loiacono [(1)] - Daniele Reverberi [(1)] - Paolo Malatesta [(1,2)]

Università degli Studi di Genova, DIMES, GENOVA, Italy (1) - IRCCS Ospedale Policlinico San Martino, Oncologia Cellulare, Genova, Italy (2)

Gliomas and immune system mutually reshape their phenotypes during malignant progression but, the mechanisms governing it are not fully elucidated. We used a glioma model, based on somatic gene transfer of PDGF-B, that recapitulates glioma progression. PDGF-B overexpression induces tumors that initially show low-grade gliomas (LG) features and do not successfully graft in immunocompetent mouse brains being highly immunostimulatory. Later on, these tumors progress into high-grade gliomas (HG) with a M2 pro-tumorigenic infiltrate and are able to generate secondary tumors when transplanted in immunocompetent mice. Interestingly, we showed that LG cells can successfully graft in immunodeficient NOD/SCID mice. To evaluate the ability of HG to modify the immune system phenotype we co-culture HG cells with freshly isolated splenocytes. Our results show that HG cells reduce the percentage of proliferating $CD8^+$ lymphocytes and drastically reduce the cytolytic activity of both $CD8^+$ and NK cells as shown by the decrease of Granzyme-B expression. Moreover, we noticed that HG cells assist the orthotopic grafting of LG cells, suggesting that the immunosuppressive environment induced by HG cells could tolerate also immunostimulatory LG cells. To dissect which immune subpopulation counteracts the growth of gliomas in the early stages of tumor progression, we orthotopically transplant LG in mice depleted for specific immune population ($CD4^+$, $CD8^+$, NK cells). We show that mice depleted for $CD4^+$ lymphocytes sustain the grafting of LG cells *in vivo* but have a dramatic reduction of $CD8^+$ infiltrating lymphocytes. Cytotoxic cells appear to serve as the ultimate effectors in immunosurveillance, and thus, the reduction of cytotoxic cell activity by glioma during tumor progression emerges as a critical determinant for achieving immunoevasion. However, this evasion mechanism may be indirectly mediated through the action of CD4 lymphocytes.

NKG2D CAR T Cells Target Pediatric Brain Tumor Cells In Vitro and in a Murine Model of Human Glioblastoma In Vivo

Marta Ibáñez Navarro (1) - Miguel Angel Navarro-Aguadero (1) - Ana de Pablos-Aragoneses (2) - Susana García-Silva (3) - Laura Clares-Villa (4) - Cristina Ferreras (4) - Hector Peinado (3) - Joaquín Martínez-Lopez (1) - Antonio Pérez-Martínez (4) - Lucía Fernández (1)

Spanish National Cancer Research Center (CNIO), H12O – CNIO Haematological Malignancies Clinical Research Unit, Madrid, Spain (1) - Spanish National Cancer Research Center (CNIO), Brain Metastasis Group, Department of Molecular Oncology, Madrid, Spain (2) - Spanish National Cancer Research Center (CNIO), Microenvironment and Metastasis Laboratory, Department of Molecular Oncology, Madrid, Spain (3) - Hospital La Paz Institute for Health Research, IdiPAZ, Translational Research in Pediatric Oncology, Hematopoietic Transplantation and Cell Therapy, Madrid, Spain (4)

Pediatric malignant Central Nervous System (CNS) tumors are the most common solid tumors and the leading cause of cancer-related death in children, underlying the need for new therapeutic approaches. In this regard, CAR T cells have emerged as a new pillar of treatment for pediatric CNS tumors. The interactions between NKG2D receptor on immune effector cells and NKG2D ligands on tumor cells are essential for tumor immunosurveillance. In the present study, we have explored the ability of NKG2D CAR T cells to target pediatric brain tumors. By using Europium-TDA cytotoxicity assays *in vitro*, we found 6 out of the 6 tested CNS tumor cells lines were sensitive to NKG2D CAR T cells lysis, with a percentage of cytotoxicity ≥30% when effector: target ratios of 20:1 were used. Furthermore, in 3D cultures, NKG2D CAR T cells showed ability to penetrate and eliminate glioblastoma tumor-spheres. In an orthotopic murine model of human glioblastoma, intracranial injections of NKG2D CAR T cells drastically reduced tumor growth, providing their potential to treat glioblastoma *in vivo*. However, NKG2D CAR T cells showed no clinical benefit when they were administered intravenously. Since the entrance of NKG2D CAR T cells to the tumor site could be hampered by the Blood Brain Barrier (BBB), we are currently exploring the permeability of the BBB to NKG2D CAR T cells by using a human BBB model. Additionally, in an attempt to facilitate homing and tumor infiltration, we have isolated Extracellular Vesicles (EVs) from NKG2D CAR T cells. At the moment, we have found EVs derived from NKG2D CAR T cells maintain CAR expression and we are now exploring their anti-tumor potential *in vitro*. In sum, although very preliminary, our results show that NKG2D CAR and Exo-NKG2D CAR could be a promising therapeutic approach to treat these tumors.

Rapamycin ameliorates the pathological phenotype in the Twitcher mouse by autophagy activation

Ambra Del Grosso (1) - Sara Carpi (2) - Miriam De Sarlo (1) - Laura Colagiorgio (1) - Luca Scaccini (1) - Alabed Husam B R (3) - Pellegrino Roberto Maria (3) - Ilaria Tonazzini (1) - Carla Emiliani (3) - Marco Cecchini (1)

Scuola Normale Superiore and Istituto Nanoscienze - CNR, NEST - National Enterprise for nanoScience and nanoTechnology, PISA, PI, Italy (1) - Università degli studi di Catanzaro della "Magna Grecia"; Istituto Nanoscienze - CNR, Dipartimento di Scienze della salute; NEST - National Enterprise for nanoScience and nanoTechnology, PISA, PI, Italy (2) - University of Perugia, Perugia Italy, Department of Chemistry, Biology, and Biotechnologies, Perugia, Italy (3)

Krabbe disease (KD) is a rare condition caused by a deficiency of the lysosomal enzyme galactosylceramidase (GALC). GALC lack leads to the accumulation of the cytotoxic metabolite psychosine (PSY) in the nervous system, with consequent demyelination and neurodegeneration. The KD-related pathogenetic mechanisms are still not completely understood, and no cure is available for KD. Recently, we demonstrated the involvement of autophagy dysfunctions in KD pathogenesis. We found p62-tagged protein aggregates in the brain of KD mice and increased p62 levels in the KD sciatic nerve. Here, we decided to test *in-vitro* and *in-vivo* the autophagy inducer Rapamycin (RAP) to remove unwanted cellular products from KD cells, such as p62 aggregates and PSY. We demonstrated that RAP can partially reinstate the WT phenotype in KD primary cells by decreasing the number of p62 aggregates. Therefore, we tested RAP in the Twitcher (TWI) mouse, a spontaneous KD mouse model. The drug has been administered ad libitum via drinking water (1,5 mg/Kg) starting from PND 21-23. We longitudinally monitored the mouse motor performance through the grip strength and the rotarod tests, and a set of biochemical parameters related to the KD pathogenesis (i.e. PSY accumulation, astrogliosis and autophagy markers expression). We found that RAP is able to improve motor functions at selected time points. Interestingly, we found that the treatment diminishes astrogliosis in the TWI brain, spinal cord, and sciatic nerves. Additionally, by western blot and immunohistochemistry, we found that RAP decreases the amount of p62 in TWI nervous tissues, confirming our *in-vitro* findings. Finally, RAP treatment demonstrated to be able to partially remove PSY in the spinal cord. Overall, these results suggest considering RAP as an option to be tested for KD clinical trials. This is especially encouraged by the fact that RAP is already in clinical practice.

Recovering STIMulation of astrocyte Ca2+ signal to shed light on Alzheimer's Disease

Annamaria Lia [1] - Gabriele Sansevero [2] - Angela Chiavegato [1] - Miriana Sbrissa [1] - Diana Pendin [3] - Letizia Mariotti [3] - Nicoletta Berardi [2] - Giorgio Carmignoto [3] - Cristina Fasolato [1] - Micaela Zonta [3]

University of Padova, Department of Biomedical Sciences, Padua, Italy (1) - CNR, Neuroscience Institute, Pisa, Italy (2) - CNR, Neuroscience Institute, Padua, Italy (3)

Alzheimer's disease (AD) is a chronic incurable neurodegenerative disorder characterized by progressive memory loss and cognitive dysfunctions. Brain function is governed by dynamic interactions between neurons and astrocytes. Noteworthy, calcium dynamics in astrocytes represent a fundamental signal that through gliotransmitter release regulates synaptic plasticity and behaviour. Here, by using cutting-edge techniques including 2-photon Ca^{2+} imaging, electrophysiology and behavioural memory tests, we present a longitudinal study in the PS2APP mouse model of AD linking astrocyte Ca^{2+} hypoactivity to memory loss. At the onset of plaque deposition, somatosensory cortical astrocytes of AD female mice swtich to a reactive pro-inflammatory state and exhibit a drastic reduction of Ca^{2+} signaling, closely associated with decreased endoplasmic reticulum Ca^{2+} concentration and reduced expression of the Ca^{2+} sensor STIM1. In parallel, astrocyte-dependent long-term synaptic plasticity declines in the somatosensory circuitry, anticipating specific tactile memory loss. Notably, we show that both astrocyte Ca^{2+} signaling and long-term synaptic plasticity are fully recovered by selective STIM1 overexpression in astrocytes. Our data unveil astrocyte Ca^{2+} hypoactivity in neocortical astrocytes as a functional hallmark of early AD stages and indicate astrocytic STIM1 as a target to rescue memory deficits.

Design of an innovative 3D model for blood-brain barrier towards improved translational medicine approaches

Stefania Scala [(1)] - Valentina Peluso [(2)] - Teresa Russo [(2)] - Salvatore Valiante [(1)] - Antonio Gloria [(3)]

Università degli studi di Napoli Federico II, Biology, Naples, Italy (1) - National Research Council of Italy, Institute of Polymers, Composites and Biomaterials IPCB, Naples, Italy (2) - Università degli studi di Napoli Federico II, Ingegneria Industriale, Naples, Italy (3)

The blood-brain barrier (BBB) is a crucial component of the central nervous system that protects the brain from harmful substances while allowing essential nutrients to pass through. Overcoming the BBB remains a crucial aspect for the delivery of drugs or therapeutics in neurological disease modelling. It is important to underline that *in vivo* models could provide experimental environments that closely mimic the complexity of human physiology, although no animal model can faithfully reproduce all the manifestations of human diseases. In this context*, in vivo* models must be interpreted as an approximation of human biology limited to particular regions or other features. The most important disadvantage of *in vivo* models is the translation of results towards human application. Furthermore, using an *in vitro* model it should be possible to closely reproduce the essential features of the human BBB *in vivo*. In this scenario, an ideal *in vitro* BBB model should have: (i)3D vessel-like structure design; (ii) multiple cell lines in co-culture cells line; (iii) flow-induced shear stress. Accordingly, a novel 3D BBB *in vitro* model has been provided adopting a bipartite vessel-like bioprinted scaffold using cell-laden sodium alginate hydrogels with two different cell lines, namely endothelial and neuronal cells. The optimization of both design and printing parameters has been carried out to achieve high-quality BBB models. The critical parameters that influence print quality and cell viability were also explored and tailored for achieving optimal results. Preliminary results suggested that cell-laden alginate hydrogels represent a valuable bioink for complex biological system modelling, also taking into account the possibility of adopting a bioreactor to study how the shear stress impacts on tight junction protein expression. This innovative *in vitro* BBB model could also be an interesting tool towards high-throughput drug screening.

HSV-1 Infection in Mouse Enteric Nervous Sistem: a Trigger for Alzheimer's Disease-Like Neurodegeneration Hallmarks

Veronica Zatta [(1)] - Paola Brun [(1)] - Ignazio Castagliuolo [(1)]

Università degli Studi di Padova, Dipartimento di Medicina Molecolare, Padova, Italy (1)

Alzheimer's disease (AD) is a neurodegenerative disease that induce progressive cognitive impairment in patients. It affects millions of people all over the world with an expected enormous increase in the next years, with a great impact on healthcare systems. In the last decades, a growing body of evidence supports the profound link between AD onset and infectious agents. Among infective agents the neurotropic Herpes simplex virus type 1 (HSV-1) is extremely attractive for a number of experimental and clinical observations. In particular, multiple HSV-1 reactivations from latency are thought to contribute to neuronal dysfunction, thus leading to neurodegeneration. To further investigate this hypothesis, our group established and characterized a mouse model of persistent HSV-1 infection (up to 10 weeks) in the enteric nervous system. By immunohistochemical staining of ileal Longitudinal Muscle/Myenteric Plexus (LMMP) we observe progressive APP (Amyloid Precursor Protein) and β-amyloid accumulation in response to HSV-1 infection, while by qPCR analysis we detect increased expression of β- and γ-secretase genes. A significant decrease in acetylcholine levels in the myenteric plexus was demonstrated via HPLC, suggesting synaptic damage. In addition, HSV-1 persistent infection induces IFNα, IFNβ, IL-1β and IL-6 overexpression in LMMP. Moreover, by immunofluorescence analysis on primary enteric neurons isolated from infected mice we found APP and β-amyloid accumulation and Tau hyperphosphorylation. Furthermore, we analysed myenteric neurons redox homeostasis, revealing peroxidation of membrane lipids and an increased mitochondrial production of free radicals in cells obtained from *in vivo* infected mice. Intriguingly, treatment of HSV-1 infected enteric neurons with a mitochondria-targeting antioxidant (MitoTEMPO) reduced β-amyloid accumulation. These preliminary *in vivo* and *ex vivo* data further support the active role of HSV-1 in AD pathogenesis.

Synaptic-dependent developmental dysconnectivity in 22q11.2 deletion syndrome

Filomena Grazia Alvino (1) - Silvia Gini (1) - David Sastre Yagüe (1) - Antea Minetti (2) - Alberto Galbusera (1) - Caterina Montani (1) - Marco Pagani (1) - Charlie Schleifer (3) - Francesco Papaleo (4) - Michael Vincent Lombardo (1) - Massimo Pasqualetti (2) - Carrie Elyse Bearden (3) - Alessandro Gozzi (1)

Istituto Italiano di Tecnologia, Centro di Neuroscienze e Sistemi Cognitivi (CNCS), Rovereto (TN), Italy (1) - University of Pisa, Unit of Cellular and Developmental Biology, Department of Biology, Pisa, Italy (2) - University of California, Semel Institute for Neuroscience and Human Behavior, Los Angeles, United States (3) - Istituto italiano di Tecnologia, Neuroscience Area, Genetics of Cognition Laboratory, Genova, Italy (4)

22q11.2 Deletion Syndrome (22qDS) is a genetic syndrome associated with increased risk of developmental disorders such as autism and schizophrenia. Brain imaging studies have shown that people with 22qDS exhibit altered large-scale functional connectivity. However, the developmental course and neural underpinnings of these alterations remain undetermined. Here, we investigated the developmental trajectory of functional connectopathy in 22qDS in both a mouse model and in human 22qDS carriers. To this aim, we longitudinally mapped resting-state fMRI connectivity in juvenile and adult LgDel mice, an established mouse model of 22qDS. We found that developmental connectopathy in LgDel mice undergoes a dramatic reconfiguration during the pubertal period, with widespread prepubertal fMRI hyper-connectivity reverting to focal hippocampal hypo-connectivity in adult LgDel mutant mice. We also found that fMRI hyper-connectivity in juvenile Lgdel mice is paralleled by a surplus of cortical dendritic spines, and that both of these phenotypes are normalized by pretreatment with the Gsk3B inhibitor SB216763. To probe the generalizability of these findings to human 22qDS, we examined fMRI connectivity in both pre-pubertal (TD=52, 22qDS n=21) and peri/post-pubertal (TD=204, 22qDS n=118) 22qDS carriers. We found that functional connectopathy in human 22qDS similarly undergoes a reconfiguration from dominant hyperconnectivity in prepubertal carriers, to hippocampal and cortical hypo-connectivity in adulthood. Prompted by our mouse investigation, we next tested the hypothesis that this reconfiguration could be driven by Gsk3B-related synaptic mechanisms. In keeping with this, we found that brain regions exhibiting functional connectivity reversal in 22qDS carriers are spatially enriched for gene transcripts encoding for synaptic proteins that interact with Gsk3B ($p=0.001$). Collectively, these findings provide evidence of synaptic-dependent, developmental dysconnectivity in 22qDS.

A novel method to estimate Multiple Sclerosis connectomes considering lesional tissue information

Sara Bosticardo (1) - Matteo Battocchio (2) - Po-Jui Lu (1) - Mario Ocampo-Pineda (1) - Xinjie Chen (1) - Laura Rustemi (1) - Sabine Schaedelin (1) - Matthias Weigel (1) - Lester Melie-Garcia (1) - Cristina Granziera (1) - Alessandro Daducci (2)

University Hospital Basel, Translational Imaging in Neurology (ThINK) Basel, Department of Biomedical Engineering, Faculty of Medicine, Basel, Switzerland (1) - University of Verona, Diffusion Imaging and Connectivity Estimation (DICE) Lab, Department of Computer Science, Verona, Italy (2)

Multiple sclerosis (MS) is a demyelinating disease of the central nervous system that leads to focal lesions that may bias structural connectivity estimates. Here we present a novel method for estimating structural connectivity in the presence of focal white matter alterations. It comprises the computation of connection weights between different gray matter (GM) regions by projecting myelin volume fraction-sensitive scalar map values onto streamlines reconstructed by tractography, modeling lesion-affected voxels, and scaling streamline weights accordingly.
We used data from 82 healthy controls (HC) (44 females, mean age 37.1±12.4) and 139 MS patients (82 females, mean age 45.4±13.8) to evaluate our method's usefulness in studying MS connectome. To assess the method's sensitivity to pathology, we extracted 4 global network metrics: mean strength, global efficiency, modularity, and clustering coefficient. We also evaluated changes in the myelination of bundles, typically affected in MS, connecting the precentral gyrus (PrCG) with the subcortical gray matter nuclei, the corticospinal tract (CS), the pons, and the corpus callosum (CC). We perform a robust linear model considering age, sex, and network density as covariates.
The results show that mean strength, efficiency, and clustering coefficient are notably higher in HCs than in MS patients ($p<0.001$, $R^2>0.40$), while modularity is significantly higher in MS patients than in HCs ($p=0.002$, $R^2=0.52$). We found that all the bundles analyzed are less myelinated in MS than in HC in both hemispheres ($p<0.01$, $R^2>0.10$ for bundles connecting PrCG to subcortical GM nuclei, and $p<0.001$, $R^2>0.10$ for CS, pons, and CC). The method demonstrates significant sensitivity to pathology-induced impairment in the structural connectivity of MS patients. In addition, it allows for precise estimation of damage caused by focal lesions without introducing any bias in the connectivity estimates, which was unreachable using state-of-the-art methods.

Immune cell migration towards the blood-brain barrier is mediated by EBI2 under inflammatory conditions

Fionä Caratis [(1)] - Bartosz Karaszewski [(2)] - Tomomi Furihata [(3)] - Aleksandra Rutkowska [(1)]

Medical University of Gdansk, Department of Anatomy and Neurobiology, Gdansk, Poland (1) - Medical University of Gdansk & University Clinical Center, Department of Adult Neurology, Gdansk, Poland (2) - Tokyo University of Pharmacy and Life Sciences, Laboratory of Clinical Pharmacy and Experimental Therapeutics, Tokyo, Japan (3)

Multiple sclerosis (MS) is a chronic, neurodegenerative and neuroinflammatory disease characterised by the entrance of peripheral immune cells into the central nervous system via a disruption of the blood-brain barrier (BBB). The infiltrating immune cells induce inflammatory signalling and attack the myelin sheaths surrounding the neuronal axons, leading to their neurodegeneration and death. The oxysterol 7α,25OHC is a natural ligand for the Epstein-Barr virus-induced receptor 2 (EBI2) which, among other functions, regulates immune cell migration. We specifically showed that brain microvessels express the EBI2 receptor as well as the enzymes necessary for 7α,25OHC synthesis (CH25H and CYP7B1) and that the oxysterol pathway is upregulated in brain's plaques in multiple sclerosis but also in the cerebrospinal fluid of MS patients. Using human in vitro BBB models, comprised of endothelial cells, pericytes and astrocytes, we characterised the expression of EBI2 and EBI2-related enzymes after inflammatory stimuli in the cells forming the BBB and how they impact the BBB permeability. Finally, we investigated immune cells migration patterns with immortalised monocytes and lymphocytes from MS and non-MS patients towards our in vitro spheroid and transwell models of the BBB. The BBB was stimulated with inhibitors of the EBI2/oxysterol pathway and showed that immune cell migration is dependent on the EBI2/oxysterol pathway. Thus, modulating this pathway directly at the BBB represents a therapeutic target in MS to prevent immune cell migration towards the brain parenchyma.

Characterization of Cerebellum Alterations in a Mouse Model of Friedreich's Ataxia

Veronica Ceci [(1)] - Francesca Sciarretta [(2)] - Daniele Lettieri-Barbato [(1)] - Katia Aquilano [(1)]

University of Rome Tor Vergata, Department of Biology, Rome (RM), Italy (1) - IRCCS, Fondazione Santa Lucia, Rome (RM), Italy (2)

Friedreich's ataxia (FA) is a neurodegenerative disease characterized by mitochondrial dysfunction due to mutation of the gene encoding the matrix protein frataxin (FXN) which is involved in iron metabolism and in the assembly of iron-sulfur clusters of mitochondrial enzymes. The *Fxn* knock-in/knockout (KIKO) mouse represents a valid model to study FA. Through a bulk mRNAseq, we found that 700 genes were differentially expressed in cerebellum of KIKO with respect to WT mice. Functional enrichment analysis of KEGG pathways revealed 4 clusters of genes pertaining to oxidative phosphorylation, myelin sheath, fatty acid metabolism and synaptic transmission. Taken together, transcriptomics findings revealed a metabolic perturbation in cerebellum of KIKO mice. We also performed qPCR analysis and found that mitochondrial biogenesis, ferroptosis/oxidative stress and inflammatory genes were altered in cerebellum of KIKO mice. Based on the findings that immune cell activation in the cerebellum induces neuronal hyperexcitability and disruption of psychomotor behaviors in animals, we focused our attention on immune cells. We isolated bone marrow-derived macrophages from WT and KIKO mice and found a higher mRNA expression of the inflammatory markers *Nos2* in KIKO than WT mice. We therefore moved at characterizing the immunophenotype of cerebellum of adult non-symptomatic KIKO mice in search for early alterations of resident immune cells. Albeit a similar abundance of microglia cells was found in cerebellum of KIKO and WT mice, KIKO microglia showed increased expression of pro-inflammatory markers. Microglia cells are important in brain development, maintenance of neuronal networks, injury repair and in the elimination of cellular debrides and protein aggregates that may endanger the CNS. Hence, the results highlight the importance to study the phagocytic activity of microglia in FA in order to find new pharmacological targets to counteract neuroinflammation and neurodegeneration.

Dendritic cells generated in the presence of specialized pro-resolving mediators display a tolerogenic effect on encephalitogenic T cells

Giada Pessina [(1)] - Marta Bottero [(1)] - Fabrizio Loiacono [(2)] - Nunzio Iraci [(3)] - Loredana Leggio [(3)] - Greta Paternò [(3)] - Silvia Ravera [(4)] - Nadia Bertola [(4)] - Santina Bruzzone [(4)] - Valerio Chiurchiù [(5)] - Nicole Kerlero de Rosbo [(1)] - Tiziana Vigo [(1)] - Antonio Uccelli [(1)] - Giovanni Ferrara [(1)]

IRCCS Ospedale Policlinico San Martino, Experimental Neuroscience, Genoa, Italy (1) - IRCCS Ospedale Policlinico San Martino, Experimental Pathology and Immunology, Genoa, Italy (2) - University of Catania, Department of Biomedical and Biotechnological Sciences, Catania, Italy (3) - University of Genoa, Department of Experimental Medicine, Genoa, Italy (4) - IRCCS Santa Lucia Foundation, Laboratory of Resolution of Neuroinflammation, European Center for Brain Research, Rome, Italy (5)

Tolerogenic dendritic cells (tDC) are a specialized subset of DC that promote immune tolerance, suppressing excessive immunogenic reaction thereby promoting milder T-cell response. The loss of immunological tolerance is a hallmark of several autoimmune diseases, such as multiple sclerosis (MS) and its animal model, the experimental autoimmune encephalomyelitis (EAE). With the perspective of designing therapeutic approaches, protocols have been developed to generate tDC but they failed in clinical applications. Accordingly, we aimed to use a new protocol to generate tDC by exposure to specialized pro-resolving mediators (SPM), a novel class of bioactive lipids involved in the resolution of inflammation. Our *in vitro* data showed that DC generated in the presence of SPM mixture (RvD1, RvE1 and MCTR1) and activated with LPS/IFNγ (DCSPM) display decreased migratory capacity, upregulation of tolerogenic markers and concomitant downregulation of pro-inflammatory markers. In addition, DCSPM reduce T cells pro-inflammatory response, mainly through a paracrine effect mediated by DC-derived extracellular vesicles (EV). Moreover, since tDC display less oxidative stress in comparison with immunogenic DC, we wondered whether SPM could influence the coupling between ATP synthesis and oxygen consumption (P/O ratio), incrementing the oxidative phosphorylation efficiency. We demonstrated coupled P/O in DCSPM and in T cells cocultured with DCSPM or treated with their secreted EV. We further hypothesized that the injection of DCSPM in EAE-affected mice could modulate the encephalitogenic response and could exert immunomodulatory effects *in vivo*. Our data on preventive and therapeutic treatment of EAE-affected mice revealed a milder disease course and decreased pro-inflammatory profile of lymph nodes. Taken together, these data suggest that DCSPM have a protective effect on EAE and could be promising candidates as a therapeutic approach for possible translation to MS.

CN04 | Human iPSC-based cellular systems to model Autosomal dominant leukodystrophy

Ingrid Battistella [1] - Pietro Cortelli [2] - Stefano Ratti [3] - Lucia Manzoli [3] - Pietro Guaraldi [2] - Mariia Zadorozhna [4] - Elisa Giorgio [5] - Luciano Conti [1]

Laboratory of Stem Cell Biology, University of Trento, Department CIBIO, Trento, Italy (1) - IRCCS Istituto delle Scienze Neurologiche di Bologna, Bellaria Hospital, Bologna, Italy (2) - University of Bologna, Department of Biomedical and Neuromotor Sciences, Bologna, Italy (3) - Laboratory of Medical Genetics, Department of Molecular Medicine, University of Pavia, Pavia, Italy (4) - Medical Genetics Unit, IRCCS Fondazione Mondino, Pavia, Italy (5)

Autosomal dominant leukodystrophy (ADLD) is a slowly, progressive, genetic, and fatal neurological disorder. The genetic cause of ADLD is Lamin B1 (LMNB1) overexpression due to coding duplications or noncoding deletions at the LMNB1 locus. Lamin B1 is a component of the inner nuclear membrane of cells and although LMNB1 is ubiquitously expressed, it appears that neurons and glial cells are particularly sensible to LMNB1 dosage. Currently, only symptomatic and palliative treatments are available for this fatal disease. Since its discovery, human induced pluripotent stem cell (hiPSC) technology has open to the generation of novel and pathological-relevant *in vitro* models for Central Nervous System human diseases, for which no appropriate model systems were available. In this work, we describe the reprogramming of peripheral blood mononuclear cells and fibroblasts from ADLD patients carrying different genetic mutations into hiPSCs by Sendai Virus-based method. These hiPSC lines were characterized to assess their pluripotency state by means of qRT-PCR and immunofluorescence assay. Also, embryoid bodies formation assay was used to evaluate their functional pluripotency. In parallel, we set up a procedure for the differentiation of hiPSCs into neuronal and astrocyte 2D cultures, and 3D cell models including hiPSCs derived forebrain and cerebellum organoids. These cultures were characterized to assess the expression of stage-specific markers and potential ADLD-relevant phenotypic and functional alterations. In conclusion, patient-derived ADLD hiPSC lines coupled with hiPSC-based technologies might represent valuable tools for studies aiming to investigate ADLD-specific alterations at molecular and cellular levels and develop potential target-specific drugs.

CN05 | CRISPR/Cas9 and piggyBac Transposon-Based Conversion of a Pathogenic Biallelic TBCD Variant in a Patient-Derived iPSC Line Allows Correction of PEBAT-Related Endophenotypes

Federica Benigni [(1)] - Valentina Muto [(1)] - Valentina Magliocca [(1)] - Rossella Borghi [(1)] - Elisabetta Flex [(2)] - Valentina Pallottini [(3)] - Alessandro Rosa [(4)] - Claudia Compagnucci [(1)] - Marco Tartaglia [(1)]

Ospedale Pediatrico Bambino Gesù, Molecular Genetics and Functional Genomics, Rome, Italy (1) - Istituto Superiore di Sanità, Department of Oncology and Molecular Medicine, Rome, Italy (2) - University Roma Tre, Science, Rome, Italy (3) - Sapienza University of Rome, Department of Biology and Biotechnologies "Charles Darwin", Rome, Italy (4)

Induced pluripotent stem cells (iPSCs) have been established as a reliable *in vitro* disease model system and represent a particularly informative tool when animal models are not available or do not recapitulate the human pathophenotype. The recognized limit in using this technology is linked to some degree of variability in the behavior of the individual patient-derived clones. The development of CRISPR/Cas9-based gene editing solves this drawback by obtaining isogenic iPSCs in which the genetic lesion is corrected, allowing a straightforward comparison with the parental patient-derived iPSC lines. Here, we report the generation of a footprint-free isogenic cell line of patient-derived *TBCD*-mutated iPSCs edited using the CRISPR/Cas9 and *piggyBac* technologies. The corrected iPSC line had no genetic footprint after the removal of the selection cassette and maintained its «stemness». The correction of the disease-causing *TBCD* missense substitution restored proper protein levels of the chaperone and mitotic spindle organization, as well as reduced cellular death, which were used as read-outs of the *TBCD* KO-related endophenotype. The generated line represents an informative *in vitro* model to understand the impact of pathogenic *TBCD* mutations on nervous system development and physiology.

CN03 | 3D-stem cell spinal cord model to study the therapeutic mechanisms of risdiplam-like compound for Spinal Muscular Atrophy

Andrea D'Angelo (1) - Francesca Beatrice (2) - Paola Rinchetti (1) - Irene Faravelli (1) - Matteo Miotto (3) - Simona Lodato (3) - Monica Nizzardo (2) - Federica Rizzo (1) - Giacomo Comi (4) - Linda Ottoboni (1) - Stefania Corti (4)

University of Milan, Dino Ferrari Centre/University of Milan/ Department of Pathophysiology and Transplantation (DEPT), Milan, Italy (1) - Foundation IRCCS Ca' Granda Ospedale Maggiore Policlinico, Foundation IRCCS Ca' Granda Ospedale Maggiore Policlinico/ Neurology Unit, Milan, Italy (2) - Humanitas Clinical and Research Center, Humanitas Clinical and Research Center, Milan, Italy (3) - Dino Ferrari Centre/Foundation IRCCS Ca' Granda Ospedale Maggiore Policlinico, Foundation IRCCS Ca' Granda Ospedale Maggiore Policlinico/University of Milan/Department of Pathophysiology and Transplantation, Milano, Italy (4)

Spinal Muscular Atrophy (SMA) is a severe neurological disorder characterized by early onset and degeneration of lower motor neurons due to mutations in the *SMN1* gene. To reproduce reliable human models, we generated and phenotypically characterized human spinal cord organoids from induced pluripotent stem cells (iPSCs) of SMA type 1 subjects (n=3) and healthy controls (n=2). Our study aimed at improving the treatment of SMA by investigating the efficacy of a Risdiplam-like compound on 3-dimensional (3D) spinal cord model. Treatment, whose main action is restoring SMN protein level, was started at different time points during the first 80 days of organoid development which parallels the first trimester post conception and was provided as daily therapy every two days. We observed that SMA samples present a pervasive cellular and molecular developmental alteration in multiple cell populations, including neural progenitors, beyond motor neurons. This was ascertained using bulk transcriptomics, single cells RNAseq, and multi-electrodes array analysis, along with immunophenotypic characterization. Our preliminary results on treatment demonstrated that 1) Risdiplam-like compound modulates at least 15% of disease affected genes; 2) long-term *in vitro* treatment is well-tolerated; 3) ratio between full length *SMN2* and Δ7 is robustly restored; 4) pathological hallmarks are reverted, all in all supporting the idea that SMA organoids represent a reliable model to explore drug kinetics and therapeutic consequences. Moreover, our study highlights the early-onset and pervasive developmental nature of SMA pathogenesis, which can be further disentangled exploiting organoids. Our study precisely contributes to the optimization of Risdiplam therapy and to the identification of targets for complementary treatment intervention in SMA patients.

CN01 | Serum NfL levels and cognitive performance in persons with multiple sclerosis

Rina Demjaha [1,2] - Stefanie Maria Charlotte Hechenberger [1,4§] - Arabella Buchmann [1,2] - Birgit Helmlinger [1,4] - Bettina Heschl [1] - Sebastian Wurth [1,3] - Peter Opriessnig [1] - Stefan Ropele [1] - David Leppert [5,6] - Pascal Benkert [6] - Jens Kuhle [5,6] - Christian Enzinger [1] - Daniela Pinter [1,4*] - Michael Khalil [1,2*§]

** shared senior author § shared corresponding author*

Medical University of Graz, Department of Neurology, Graz, Austria (1) - Medical University of Graz, Neurology Biomarker Research Unit, Graz, Austria (2) - Medical University of Graz, Division of Neuroradiology & Interventional Radiology, Department of Radiology, Graz, Austria (3) - Medical University of Graz, Research Unit for Neuronal Plasticity and Repair, Graz, Austria (4) - Neurologic Clinic and Policlinic, MS Centre and Research Centre for Clinical Neuroimmunology and Neuroscience Basel, University Hospital Basel, University of Basel, Basel, Switzerland (5) - Department of Clinical Research, Clinical Trial Unit, University Hospital Basel, University of Basel, Basel, Switzerland (6)

Introduction: Serum neurofilament light (sNfL) is a robust biomarker to indicate neuro-axonal damage in various neurologic conditions including multiple sclerosis (MS). Cognitive impairment (CI) is a frequent feature in MS with a huge impact on quality of life and social functioning. It is still not clear if sNfL correlates with or even predicts CI in MS. This study aims to elucidate the association between sNfL and CI in persons with MS (pwMS).

Methods: 186 pwMS (112 female; mean age=39.6±10.4; mean disease duration=10.6 years; median EDSS=1.5 (IQR=2.75)) and 49 healthy controls (HC) (35 females; mean age=33.4±10.7) underwent clinical examination, neuropsychological (Brief Cognitive Assessment for MS-BICAMS) and 3T brain-MRI assessment, including T2-hyperintense lesion load and normalized brain volumes calculations. sNfL was quantified by single molecule array (Simoa SR-X). We calculated sNfL Z-scores corrected for age and body-mass-index; Symbol Digit Modalities Test (SDMT) Z-scores corrected for age and education; Verbal Learning Memory Test (VLMT) and Brief Visuospatial Memory Test (BVMT) T-scores corrected for age.

Results: In this cross-sectional analysis, 48 pwMS showed CI in at least one BICAMS test and 38 in SDMT (M=-0.2±1.1); 6 in VLMT (M=56.2±9.5) and 20 in BVMT (M=54.3±12.7) in comparison to no CI in HC ($p<0.001$). Baseline sNfL Z-scores (M=0.73±1.27) were unrelated to BICAMS sub-tests, including the SDMT, VLMT and BVMT (all $p>0.05$, n.s.) both in pwMS and HC.

Conclusion: In this cross-sectional analysis, sNfL was unrelated to CI in pwMS. Longitudinal analyses investigating the relation of sNfL dynamics and MRI metrics with cognitive decline are currently ongoing.

Disclosures: M. Khalil has received speaker honoraria from Bayer, Novartis, Merck, Biogen Idec and Teva Pharmaceutical Industries Ltd. and serves on scientific advisory boards for Biogen Idec, Merck Serono, Roche, Novartis, Bristol-Myers Squibb and Gilead. He received research grants from Teva Pharmaceutical Industries, Ltd., Biogen and Novartis. • S. Wurth has participated in meetings sponsored by, received honoraria or travel funding from Allergan, Biogen, Ipsen Pharma, Merck, Novartis, Roche, Sanofi Genzyme, Teva and Bristol Myers Squibb. • D. Leppert is Chief Medical Officer of GeNeuro. MW received speaker honoraria from Novartis Pharma, Chugai Pharmaceutical, Biogen Japan and Alexion. • S. Hechenberger has received speaker honoraria from Roche and Bristol Myers Squibb. • B. Helmlinger has received speaker honoraria from Roche and travel funding from Janssen. • R. Demjaha has received travel funding from Janssen. • All other authors have nothing to disclose.

CN06 | Identification of novel antibodies in patients with small fiber neuropathy

Luana Morelli (1) - Fortuna Ricciardiello (2) - Alex Incensi (2) - Sara Parisini (2) - Vincenzo Angelo Donadio (2) - Rocco Liguori (1) - Maria Pia Giannoccaro (1)

University of Bologna, Department of Biomedical and Neuromotor Sciences, Bologna, Italy (1) - IRCCS, Istituto delle Scienze Neurologiche di Bologna, Bologna, Italy (2)

Small fiber neuropathy (SFN) is a peripheral neuropathy that manifests clinically with pain and autonomic disturbances. Over 50% of cases are idiopathic SFN (iSFN). Some studies suggest a possible autoimmune etiology; in particular, the presence of antibodies against FGFR3 has been identified in about 20% of patients with iSFN. In addition, a recent study suggested a possible role of antibodies to MX1, DBNL, and KRT8. Our aim was to evaluate the frequency of antibodies against FGFR3, MX1, DBNL, and KTRT8 in patients with suspected SFN. We included consecutive patients undergoing skin biopsy at the leg and thigh for suspected SFN afferent to the *UOC Neurological Clinic* of Bologna. Serum was tested for the presence of antibodies (Ab) against FGFR3, MX1 and DBNL by *in-house* cell-based assay. Small-fiber pathology was confirmed by a reduction of intraepidermal nerve-fiber (IENF) density. A total of 315 patients were investigated. Antibodies against the targets of interest were identified in 4/315 cases. Specifically, FGFR3-Ab were detected in 1/298, MX1-Ab in 2/296 and DBNL-Ab in 1/309. All positive patients had small fiber neuropathy on skin biopsy. Case 1 with MX1-Ab (M, 86 years old) showed somatic and autonomic SFN, whereas case 2 (M, 79 years old) showed only somatic fiber involvement. The patient with FGFR3-Ab (F, 46 years old) presented a somatic SFN with minimal autonomic fiber involvement distally. Finally, the patient with DBNL-Ab (F, 45 years old) showed somatic and autonomic SFN. In conclusion, antibodies against FGFR3, MX1, DBNL, and KRT8 are rare in patients with iSFN.

CN02 | Cognitive frailty and oxygen-ozone therapy: differential expressed genes as predictive biological markers of response/improvement to treatment

Cristian Bonvicini (1) - Catia Scassellati (2) - Antonio Galoforo (3) - Miriam Ciani (1) - Cristina Geroldi (4) - Ivan Arisi (5) - Valeria Manzini (6) - Chiara D'Amelio (7) - Tommaso Gosetti di Sturmeck (5) - Rossella Brandi (8) - Elisabetta Mori (9) - Mario Costa (10) - Mara D'Onofrio (5) - Antonino Cattaneo (9)

IRCCS Istituto Centro San Giovanni di Dio Fatebenefratelli, Molecular Markers Laboratory,, Brescia, Italy (1) - IRCCS Istituto Centro San Giovanni di Dio Fatebenefratelli, Biological Psychiatry Unit, Brescia, Italy (2) - Oxygen-Ozone Therapy Scientific Society (SIOOT), University of Pavia, Pavia, Italy (3) - IRCCS Istituto Centro San Giovanni di Dio Fatebenefratelli, Alzheimer Unit, Brescia, Italy (4) - European Brain Research Institute (EBRI) Rita Levi-Montalcini, Bioinformatics Facility, Roma, Italy (5) - Sapienza University of Rome,, Department of Biology and Biotechnologies C. Darwin, Roma, Italy (6) - Catholic University of Sacred Heart, Department of Neurosciences, Roma, Italy (7) - Army Medical Center Rome, Scientific Department, Roma, Italy (8) - Scuola Normale Superiore, Bio@SNS, Pisa, Italy (9) - CNR, Pisa, Bio@SNS, Pisa, Italy (10)

Frailty is a multidimensional geriatric syndrome characterized by increased vulnerability to stressors as a result of the reduced functional capacity of different physiological systems. This heterogeneous clinical syndrome, conceived as an innovative multidimensional concept, includes cognitive frailty (CF). Oxygen-Ozone (O_2-O_3) therapy is a no-invasive/no-pharmacological low-cost procedure with no side effects based on effects of low O_3 concentrations. This therapy induces a mild oxidative stress stimulating antioxidant defenses and prevents the inflammatory response and cell damage. We hypothesized that O_2-O_3 therapy might promote a significant effect on those oxidative and inflammation processes, strongly altered in CF. Specifically, we aim to identify *in vivo* predictive peripheral biological markers of response/improvement to treatment, integrating clinical and neuropsychological parameters with -omics analysis. We conducted the first pilot double blind randomized controlled trial: seventy-two elderly frail subjects aged between 65 and 80, were treated with free rectal insufflations of air, O_2 or O_2-O_3 mixture for 5 weeks (3 sessions for week). A total amount of 150cc of O_2-O_3 mixture at the concentration of 30 µg of O_3 per cc of O_2 over a 5-10 min period was administered. Subjects were monitored at different times: before (T0), 3 months (T1) and 9 months post-treatment (T2) while the RNA profiling was analysed in whole blood by Agilent microarray at T0 and T1. Based on CF evaluation, patients were divided in 3 sub-groups as Low, Moderate and High levels. Following O_2-O_3 treatment differentially expressed genes (DEGs) have been observed in the 3 subgroups. Those DEGs involve several molecular pathways highlighting a specific mRNA response to the O_2-O_3 treatment and representing potential biomarkers associated to O_2-O_3 therapy. Lastly, these biomarkers integrated with clinical data, might allow to predict and optimize the therapeutic response to O_2-O_3 therapy.

EBN01 | EPM1 3D human model: altered synaptic plasticity and neuronal morphology

Natalia Abate [(1)] - Amelia Pizzella [(1)] - Eduardo Penna [(2)] - Elisa Frenna [(1)] - Mario De Gregorio [(1)] - Laura Canafoglia [(3)] - Carla Perrone-Capano [(4)] - Silvia Cappello [(5)] - Marianna Crispino [(1)] - Rossella Di Giaimo [(1)]

University of Naples, Department of Biology, Naples, Italy (1) - Western University of Health Sciences,, College of Osteopathic Medicine of the Pacific, Pomona, United States (2) - Fondazione IRCCS Istituto Neurologico Carlo Besta, Department of Pediatric Neurology, Milan, Italy (3) - University of Naples, Department of Pharmacy, Naples, Italy (4) - Max Planck Institute of Psychiatry, Department of Developmental Neurobiology,, Munich, Germany (5)

Progressive myoclonic epilepsy 1 (EPM1) is a neurodegenerative disease characterized by loss-of-function mutations in Cystatin B (CSTB) gene. CSTB is an inhibitor of lysosomal cathepsins and it is involved in the development of human brain cortex: low levels of functional CSTB impact cell proliferation, differentiation and interneuron recruitment during neurogenesis. We previously demonstrated that CSTB is locally synthesized in rat brain synaptosomes and secreted into the media, suggesting its role in synaptic plasticity. In this work we investigate if synaptic physiology is impaired by pathological low levels of CSTB in human Cerebral Organoids (hCOs) from EPM1 patients. We showed that the synaptosomal fraction isolated from control hCOs at different developmental stages is enriched in pre-synaptic proteins and contains CSTB. CSTB presence in the synaptic territories was confirmed also by immunostaining on human neurons *in vitro*. Interestingly, CSTB is released by synaptosomes as a soluble protein and in extracellular vesicles-mediated manner. In synaptosomes from EPM1 hCOs, the levels of pre-synaptic proteins were significantly reduced and the extracellular vesicles trafficking was impaired. Furthermore, the expression levels of initiation factor linked to local protein synthesis was reduced in synaptosomes from EPM1 hCOs, suggesting an impairment in the synaptic translation system in the pathology. In addition, neurons differentiated from EPM1 patients NPCs showed longer, thinner and more branched neurites compared to controls, suggesting that altered neuronal morphology and connectivity are associated with the pathology. Altogether, these data indicate alteration of synaptic plasticity and neuronal morphology in EPM1, opening new venues toward the understanding of molecular mechanisms underlying the disease.

EBN08 | Metabolic supplementations and epigenetic alterations in in vitro AGC1 deficiency models

Giorgia Babini [(1)] - Eleonora Poeta [(1)] - Francesca Massenzio [(1)] - Federico Manuel Giorgi [(1)] - Laura Mercolini [(1)] - Massimo Lasorsa [(2)] - Barbara Monti [(1)]

University of Bologna, Dept. of Pharmacy and Biotechnology, Bologna, Italy (1) - University of Bari, Dept. of Biosciences, Biotechnologies and Biopharmaceutics, Bari, Italy (2)

AGC1 deficiency is an ultra-rare demyelinating disease caused by mutations in the SLC25A12 gene, which encodes for isoform 1 of the mitochondrial aspartate-glutamate carrier (AGC1). The main pathological features are secondary hypomyelination, along with impaired proliferation of brain cells. Probably, abnormal myelin production is due to a reduced synthesis of N-acetyl-aspartate (NAA), from which the acetyl groups mainly derive. This, in turn, leads to epigenetic alterations in the brain precursor cells and resulting in transcriptional dysregulation, causing proliferation and differentiation defects, as demonstrated by previous data on our *in vitro* AGC1 deficiency models (precursor cells of mouse oligodendrocytes -OPCs- where SLC25A12 is silenced by a shRNA and neurospheres by mouse model of AGC1 deficiency) of our laboratory.
Along with epigenetic alterations, lower production of NAA leads specifically to reduced levels of acetyl-CoA, involved in a large number of biological activities, including the synthesis of fatty acids, major components of the myelin sheath. Thus, an alteration of their production leads to hypomyelination. This is confirmed by RNA-seq analysis on OPCs, which show altered expression of transcriptional factors and enzymes involved in the fatty acid synthesis pathway.
Firstly, we would like to verify the *in silico* data of the RNA-seq analysis. Moreover, to try to compensate for the lack of acetyl-CoA, and the consequent epigenetic and metabolic alterations, supplementations with amino acids and ketone bodies -directly involved in the synthesis of acetyl-CoA- will be carried out to induce a potential recovery of differentiation/proliferation defects in both our *in vitro* models.

EBN04 | N-acetyl cysteine rescues cortical glial cell populations and results in functional improvements in a mouse model of primary autosomal recessive microcephaly 17 (MCPH17)

Maryam Khastkhodaei Ardakani [(1)] - Cecilia Astigiano [(1)] - Anna Incerti Tinterri [(1)] - Francesco Ferrini [(2)] - Chiara La Rosa [(3)] - Roberta Schellino [(1)] - Marina Boido [(1)] - Serena Bovetti [(3)] - Annalisa Buffo [(1)] - Enrica Boda [(1)]

Neuroscience Institute Cavalieri Ottolenghi, Dept. of Neuroscience , Università degli Studi di Torino, Torino, Italy (1) - Dept. of Veterinary Sciences, Dept. of Veterinary Sciences, Università degli Studi di Torino, Torino, Italy (2) - Neuroscience Institute Cavalieri Ottolenghi, Dept. of Life Sciences and Systems Biology, Università degli Studi di Torino, Torino, Italy (3)

Primary autosomal recessive microcephaly 17 (MCPH17) is a rare neurodevelopmental disorder caused by mutations in the CIT gene, which encodes for the Citron Kinase (CIT-K), a kinase involved in DNA repair and cytoskeletal dynamics. Patients show reduced brain volume, intellectual disability, motor deficits, epilepsy, and early mortality. Cit-k KO mice recapitulate MCPH17 phenotype. In the Cit-k KO mouse brain, DNA damage and reactive oxygen species (ROS) accumulation is accompanied by neural progenitor apoptosis and glial cell alterations, including oligodendroglia and astroglia reduction, hypomyelination, and increased numbers of microglia presenting dysmorphic features and engulfed synaptic material. To identify pharmacological treatments that can reduce cellular damage accumulation and improve the pathological features in Cit-k KO mice, we chronically treated Cit-k KO mice during the first 2 postnatal weeks with the FDA-/EMA-approved antioxidant drug N-acetylcysteine (NAC), which can pass the blood-brain-barrier. Treated mice showed motor improvement, reduction of epileptic myoclonus and decreased susceptibility to epileptogenic drugs, in association with a slight increase in the Cit-k KO mouse life span. These changes were accompanied by reduced brain ROS levels and by a prominent increase in the number of cortical oligodendroglia and astrocytes, though in the absence of a rescue of myelination. Moreover, microglia density and dysmorphic features decreased. Notably, deposition of perineuronal nets around cortical parvalbumin-positive interneurons was also significantly rescued by NAC treatment, suggesting a positive effect on the maturation/function of inhibitory neurons. Our data show that NAC treatment pervasively improves brain pathology and mitigates MCPH17 mouse phenotype. They also suggest that NAC-induced functional improvements may be at least in part mediated by the correction of Cit-k KO glial cell dysfunctions. Ongoing analyses will define the cellular and molecular bases of NAC effects.

EBN05 | Characterization of Early Communicative Deficits and Social Behaviors in a Mouse Model of Cdkl5 Deficiency Disorder

Nicola Mottolese (1) - Celeste Ferraguto (2) - Elisabetta Ciani (1) - Susanna Pietropaolo (2)

University of Bologna, Dept. Biomedical and NeuroMotor Sciences, Bologna, Italy (1) - Institut de Neurosciences Cognitives et Intégratives d'Aquitaine, University of Bordeaux, Bordeaux, France (2)

Mutations in the X-linked *CDKL5* gene cause CDKL5 deficiency disorder (CDD), a rare neurodevelopmental disease characterized by early onset epilepsy, severe intellectual disability, and autistic features. To date, no therapies are available for CDD. Mouse models that mimic the genetic disease, the *Cdkl5* knockout (KO) mice, were recently generated to study the pathophysiological mechanisms of CDD. *Cdkl5* KO mice recapitulate different features of CDD, showing impairments in hippocampus-dependent learning and memory, visual deficits, and autistic features. However, although CDD is an early onset neurodevelopmental disorder, most of the studies published so far have been conducted on adult *Cdkl5* KO mice, and the role of CDKL5 in the first weeks of life has not been thoroughly investigated. Importantly, none of the existing *Cdkl5* mouse models have been assessed for a thorough characterization of early communicative deficits through ultrasonic vocalizations (USVs), which could provide insights into brain circuit disruptions relevant to autism spectrum disorder (ASD) and language impairments. The aim of this study was to characterize communication in newborn/juvenile *Cdkl5* KO mice and to further investigate social behaviors during adulthood. We recorded USVs and conducted open field and auditory startle tests in newborn/juvenile mice. In addition, to evaluate social behaviors in adulthood, mice were subjected to the three-chamber test and direct social investigation towards a conspecific. We found that *Cdkl5* KO mice exhibited early quantitative and qualitative alterations in USVs and showed impairments in social behaviors, together with hyperacusis and higher levels of anxiety compared to wild-type mice. Our findings provide, for the first time, a comprehensive characterization of communication and social deficits in *Cdkl5* KO mice during early life phases, which could be useful to test therapeutic interventions for ASD in CDD patients.

EBN11 | Further insights into Allan-Herndon-Dudley syndrome: characterization of two genetic variants in SLC16A2 gene

Letizia Esposito (1) - Federica Rey (2) - Erika Maghraby (3) - Gianvincenzo Zuccotti (1) - Davide Tonduti (1) - Stephana Carelli (1) - Cristina Cereda (4)

Pediatric Research Center "Romeo ed Enrica Invernizzi", Department of Biomedical and Clinical Sciences, University of Milan, Milan, Italy, University of Milan, Children's Hospital, Milan, Italy (1) - Pediatric Research Center "Romeo ed Enrica Invernizzi", Department of Biomedical and Clinical Sciences, University of Milan, Milan, Italy, University of Milan, Milan, Italy (2) - Department of Biology and Biotechnology "L. Spallanzani, University of Pavia, Milan, Italy (3) - Center of Functional Genomics and Rare Diseases, Department of Pediatrics, Buzzi Children's Hospital, Milan, Italy, Buzzi Children's Hospital, Milan, Italy, Milan, Italy (4)

Genetics variants in SLC16A2 gene encoding for the monocarboxylate transporter 8 (MCT8) cause a severe X-linked intellectual deficit and neurological impairment known as Allan-Herndon-Dudley syndrome (AHDS). MCT8 promotes cellular uptake and efflux of thyroid hormone and its mutations provoke elevated serum T3 levels in children. Iodothyronine deiodinases (DIO) 1 and 2 are implicated in the conversion of T4 into biologically active T3, while DIO3 converts T4 into the inactive hormone reverse T3 (rT3). Active T3 and retinoid X receptors (RXR) can form heterodimer complexes which bind to hormone response elements (HREs) that leads to activate or repress transcription. Our aim is to investigate the impact of MCT8 mutations on the pathogenetic mechanisms of AHDS. Fibroblasts were obtained from skin biopsies of 2 AHDS and matched controls. To evaluate both MCT8 and thyroid hormone signaling pathway related genes expression, RNA was extracted with TRIzoL™ and assessed by Real-Time PCR. Protein expression was valuated via western blot and immunofluorescence. MTT assay was used to compare cell viability. Live and dead assay was used to discriminate live and dead populations. Lipids were detected via oil red o staining. MTT assay demonstrated a reduced cell viability as consequence of mutations in SLC16A2. We report that SLC16A2 RNA expression in AHDS patients was extremely reduced in comparison with total RNA from healthy controls. Additionally, DIO2, progastricsin, HR and KLF9 RNA expression resulted upregulated, whilst DIO1, DIO2-AS1, DIO3 and TH were downregulated influencing T_3 cell entrance. Myelin related genes were significatively reduced. The lipid staining revealed an increasing presence of lipid droplets in AHDS patients. Taken together, our preliminary data emphasize an impairment in AHDS fibroblasts in relation to mutations in MCT8 transporter, increasing our understanding in the pathogenic mechanism of mutation in two patients affected by AHDS.

EBN13 | CACNA1A mutations impair neuronal induction and function

Ilaria Musante [(1)] - Lorenzo Muzzi [(2)] - Fanny Jaudon [(3)] - Lorenzo Cingolani [(3)] - Federico Zara [(4)] - Paolo Scudieri [(4)]

IRCCS Istituto Giannina Gaslini, UOC Genetica medica, Genova, Italia (1) - Università di Genova, DIBRIS, Genova, Italia (2) - Università di Trieste, Dipartimento di Scienze della Vita, Trieste, Italia (3) - IRCCS Istituto Giannina Gaslini/ UOC Genetica medica, Università di Genova/ DINOGMI, Genova, Italia (4)

CACNA1A encodes the pore-forming α1A subunit of the voltage-gated CaV2.1 calcium channel. This channel is found primarily on presynaptic terminals, dendrites, and neuroendocrine cells in the brain, and it is critical on regulating synaptic function. Many mutations have been identified in *CACNA1A*, causing different neurological disorders, as various forms of ataxia, epilepsy, and migraine. However, the molecular mechanisms underlying these disorders are little known, and specific therapeutic approaches are lacking.
The aim of this study was to investigate the effects of *CACNA1A* loss-of-function mutations on neuronal development and function by using human-derived *in vitro* models. Accordingly, iPSCs carrying two *CACNA1A* variants causing episodic ataxia type 2 (Y1854X, selectively affecting CaV2.1[EFa] isoform, and F1491S, affecting both CaV2.1[EFa] and [EFb] isoforms) have been produced by CRISPR/Cas9 methods. Mutated and isogenic control iPSC lines were used to generate neuronal cultures by differentiation protocols passaging through embryoid bodies, neural rosettes, neural progenitors, and neuronal network stages. Morphological, molecular, and functional tests highlighted different neurodevelopmental defects caused by the two mutations. Cells carrying F1491S showed an impaired neuronal induction at very early stages, with mutated neural progenitors appearing with an altered morphology, reduced expression of neural markers, and enhanced migration. Instead, cells carrying Y1854X behaved apparently normal in terms of neuronal specification and maturation but showed a reduced spontaneous electrophysiological activity with lack of synchronous network events.
Our findings highlighted novel roles of CaV2.1 in neuronal induction, besides confirming its relevance in synaptic communications. Importantly, the iPSC-derived neuronal models developed in this study will pave the way for future therapeutics testing for neurological disorders involving *CACNA1A*.

EBN14 | Dopamine Transporter DNA Methylation modulation evoked by stress in university students

Alessandro Piccinini (1) - Eugenia Annunzi (1) - Loreta Cannito (2) - Irene Ceccato (3) - Alberto Di Domenico (3) - Rocco Palumbo (3) - Carmine Merola (1) - Riccardo Palumbo (4) - Claudio D'Addario (1)

Università degli studi di Teramo, Dipartimento di Bioscienze, Teramo, Italy (1) - University of Foggia, Department of Humanities, Foggia, Italy (2) - "G. d'Annunzio" University of Chieti--Pescara, Department of Psychological - Health and Territorial Sciences, Chieti, Italy (3) - University of Chieti--Pescara, Center for Advanced Studies and Technology, Chieti, Italy (4)

Stress can be defined as a physiological and psychological response to environmental changes that can affect well-being. We examined how stress can modulate the transcriptional regulation of key signalling pathways. Perceived Stress Scale-10 (PSS-10) at two time points within a year of each other was adopted as the measure of perceived stress level of university students and DNA methylation status at specific CpG sites of Oxytocin Receptor (OXTR), Dopamine transporter (DAT), and Serotonin transporter (SERT) genes was analysed at the same time points by pyrosequencing in DNA collected from salivary samples. PSS scores ranging from 0 – 13 are considered as low self-perceived stress, those ranging from 14 – 26 as moderate self-perceived stress, and those ranging from 27–40 as high self-perceived stress. At the first timepoint, significant increases have been observed for DNA methylation at DAT CpGs 1, 5, and 7 and in the average of all the CpG sites under study when comparing subjects with low and medium PSS scores to those with high scores. When we analysed DAT DNA methylation considering the PSS scores one year later, we observed that in people showing a reduction from high to medium or low scores, the alterations in the epigenetic mark disappeared, resulting also similar to the levels of controls. No changes between the two time points were observed for OXTR and SERT DNA methylation among students with different PSS scores. This study focuses on the dynamic nature of stress and DNA methylation patterns, biological markers that might have potential applications in stress management and interventions.

EBN21 | Novel Frontiers in Aicardi-Goutières Sydrome: Characterization of a RNU7-1 Mutation

Erika Maghraby [1] - Federica Rey [2] - Letizia Esposito [2] - Gabriele Panzeri [2] - Gianvincenzo Zuccotti [2] - Davide Tonduti [3] - Stephana Carelli [4] - Cristina Cereda [4]

Department of Biology and Biotechnology "L. Spallanzani", University of Pavia, Pediatric Research Center "Romeo ed Enrica Invernizzi", Department of Biomedical and Clinical Sciences, University of Milan, Pavia, Italy (1) - University of Milan, Pediatric Research Center "Romeo ed Enrica Invernizzi", Department of Biomedical and Clinical Sciences, Milan, Italy (2) - Buzzi Children's Hospital, Milan, Department of Pediatrics, Milan, Italy (3) - Buzzi Children's Hospital, Milan, Center of Functional Genomics and Rare Diseases, Department of Pediatrics, Milan, Italy (4)

Aicardi-Goutières Syndrome (AGS) is a rare and genetically determined pediatric disorder majorly defined by chronic lymphocytosis and raised levels of type I interferon-alpha (IFN-α) in the absence of demonstrable brain infections. Considering genetic aspects, AGS is associated with mutations in 9 genes (*TREX1, RNASEH2A, RNASEH2B, RNASEH2C, SAMHD1, ADAR, IFIH1, LSM11* and *RNU7-1*) which all encode for proteins involved in metabolism and nucleic acids uptake. Mutations in the *RNU7-1* gene (AGS9) lead to the least characterized form of the disease. This gene encodes for a small nucleolar RNA which is a member of the small nuclear ribonucleoprotein complex (U7 snRNP). It has been demonstrated that U7 snRNP is essential during the maturation of pre-mRNA of replication-dependent histones (RDH) as it leads to the cut of the Poly-A tail in these transcripts. The main aim of this work is to dissect the role of *RNU7-1* mutation in AGS pathogenesis. Specifically, we investigated canonical AGS features such as the upregulation of IFN-α, interferon-stimulated genes (ISGs) and specific outcomes of *RNU7-1* mutation in primary fibroblasts obtained from AGS patients and compared to healthy controls. Total RNA was extracted using TRIzol reagent and the genes' expression levels were determined by Real Time PCR. ELISA, Western Blot analysis and immunofluorescence were also performed to assess protein expression levels. Our results confirm the upregulation of ISGs and the typical "interferon signature" in AGS patients which cause an increase in IFN-α production. Moreover, we confirmed the modulation of this "interferon signature" through hydroxychloroquine treatment. Lastly, we assessed the increased presence of enriched Poly-A RDH transcripts confirming the role of U7 snRNP in the cleavage of Poly-A tail. In conclusion, our work describes the molecular mechanisms involved in AGS9 mutation which lead to the upregulation of ISGs, IFN-α overproduction, and misprocessing of RDH mRNAs.

EBN22 | Mutations in the stretch-activated ion channel TMEM63B associate with developmental and epileptic encephalopathies and progressive neurodegeneration

Cristiana Pelorosso (1) - Annalisa Vetro (1) - Simona Balestrini (1) - Alessio Masi (2) - Guido Mannaioni (2) - Valerio Conti (1) - Renzo Guerrini (1)

Meyer Children's Hospital IRCCS, Neuroscience Department, Florence, Italy (1) - University of Florence, Department of Neuroscience, Psychology, Drug Research and Child Health (NeuroFarBa), Section of Pharmacology and Toxicology, Florence, Italy (2)

By converting physical forces into electrical signals or triggering intracellular cascades, stretch-activated ion channels (SACs) allow the cell to respond to osmotic and mechanical stress. Knowledge of the pathophysiological mechanisms underlying associations of SACs with human disease is limited. Here we describe 17 unrelated patients, with severe early onset developmental and epileptic encephalopathy (DEE), intellectual disability, and severe motor and cortical visual impairment, associated with progressive neurodegenerative brain changes, carrying ten distinct heterozygous variants of *TMEM63B*, encoding for a highly conserved SAC. The variants occurred *de novo* in 16/17 patients for whom parental DNA was available and either missense, including the recurrent V44M in 7/17 patients, or in-frame, all affecting conserved residues located in transmembrane regions of the protein. In 12 patients, haematological abnormalities co-occurred, such as macrocytosis and haemolysis, requiring blood transfusions in some. We modelled six variants (V44M, R443H, T481N, G580S, R660T, and F697L), each affecting a distinct transmembrane domain of the channel, in transfected Neuro2a cells and demonstrated leak inward cation currents across the mutated channel even in isotonic conditions, while the response to hypo-osmotic challenge was impaired, as were the Ca^{2+} transients generated under hypo-osmotic stimulation. In conclusion, TMEM63B-associated DEE represents a novel clinicopathological entity in which altered cation conductivity results in a severe neurological phenotype with progressive brain damage and early onset epilepsy, associated with haematological abnormalities in most patients.

EBN24 | Epileptiform activity in a non-epileptic control rat: spontaneous syndrome or lesion-induced epilepsy?

Beatrice Casadei Garofani [(1)] - Stefania Bartoletti [(1)] - Arianna Capodiferro [(1)] - Federica Raimondi [(1)] - Elisa Ren [(1)] - Giulia Curia [(1)]

University of Modena and Reggio Emilia, Department of Biomedical, Metabolic and Neural Sciences, Modena, Italy (1)

Epilepsy is a pathological condition characterized by recurrent seizures that affects around 50 million people worldwide. Based on human clinical data, we hypothesize that spontaneous pathology may occur even in animals. Here, we report the case of a non-epileptic control (NEC) rat that showed electrographic ictal and interictal patterns, similar to what was described in the animal model of epilepsy. While some cases of seizures were reported in NEC rats, interictal activity has never been described in non-epileptic rodents. We carried out a video-EEG analysis in NEC adult Sprague Dawley male rats as a control group of rats with temporal lobe epilepsy of a larger project. NEC rats were injected intraperitoneally with saline; 2 weeks later epidural electrodes in the frontal cortex and depth electrodes in the hippocampal areas were implanted. Video-EEG recordings were performed over a period of 14 weeks, and recordings were 24-h long to monitor the circadian cycle of the electrical brain activity. Brains were then extracted, sliced, and used for histological staining to verify the correct position of the electrodes and the presence of any damage due to technical problems during the surgery. While 3 NEC rats showed normal electrographic activity in all the recorded traces, 1 animal exhibited interictal and ictal activity starting from week 5 post-injection, often correlating to scratching or facial automatisms corresponding to stages 1 and 2 of the Racine scale. We present here a descriptive analysis of the ictal and interictal activities from this rat. According to our preliminary results, we have hypothesized that this rat may have spontaneous epilepsy; however, histological data must demonstrate that there is no damage due to a suboptimal electrode implantation.

EBN07 | Therapeutic Opportunities in Lafora Disease

Gabriele Trentini (1) - Giulia Cazzanelli (1) - Andrea Dalle Vedove (1) - Nicolò Sbardellati (1) - Giuseppe D'Orsi (2) - Orazio Palumbo (2) - Massimo Carella (2) - Andrea Astolfi (3) - Maria Letizia Barreca (3) - Graziano Lolli (1)

University of Trento, CIBIO, Trento, Italy (1) - IRCCS Casa Sollievo della Sofferenza, UOC Genetica Medica, San Giovanni Rotondo, Italy (2) - University of Perugia, Dipartimento di Scienze Farmaceutiche, Perugia, Italy (3)

Lafora disease (LD) is a lethal autosomal recessive epilepsy, caused by loss-of-function mutations in EPM2A (encoding laforin) or NHLRC1 (expressing malin). In healthy condition, malin ubiquitinates its targets, among which protein targeting to glycogen (PTG), in a laforin dependent manner. PTG is a master regulator of brain glycogen synthesis, which is mainly metabolized and stored in astrocytes as glucose source for neurons. In LD, PTG escapes from degradation due to dysfunctional laforin-malin complex, leading to the accumulation of abnormal glycogen and neurodegeneration. Nowadays, no treatments are available as well as cellular models and clinically relevant targets for screening and drug development campaigns. In this window of opportunities, two parallel strategies are being investigated.

On one hand, a reverse chemogenomic approach has been employed with PTG as therapeutic target. The crystal structure of the PTG carbohydrate binding motif 21 (CBM21) has been determined for the first time. Hence, the molecular docking on PTG-CBM21 uncovered two potential druggable pockets. In the next future, the screening of compound libraries will be run to identify PTG-interacting molecules in order to design specific inhibitors.

On the other hand, a forward chemogenomic approach is being explored to screen FDA-approved drugs. To set up HTS experiments, different phenotypic assays to detect glycogen have been tested and validated. Moreover, genetically modified cell lines and LD patient-derived iPSCs have been developed and characterized as LD cellular models.

In this work, a druggable target involved in glycogen synthesis has been identified, aiming to reduce brain glycogen accumulation in LD. Meanwhile, the first LD-patient derived iPSCs have been reprogrammed and characterized to generate an *in vitro* model of LD.

Taken together, these findings will allow a multimethodological approach for drug screenings looking for candidates to be advanced to pre-clinical testing.

NO04 | Gold nanoparticles (AuNPs) in the radio-sensitization of glioblastoma cells

Laura Coppola (1) - Giovanna Navarra (1) - Beatrice Savarese (1) - Giorgio Avilia (1) - Chiara Laezza (2) - Maurizio Bifulco (1) - Cristina Pagano (1)

Università degli Studi di Napoli Federico II, Dipartimento di Medicina Molecolare e Biotecnologie Mediche, Napoli, Italy (1) - Consiglio Nazionale delle Ricerche - CNR, Center for Experimental Endocrinology and Oncology, Napoli, Italy (2)

Glioblastoma multiforme (GBM) is the most malignant form of primary brain tumour, with extremely poor prognosis due to bad response to therapeutic regimens. Ionizing radiation (IR) has been identified as a crucial treatment for GBM following surgical resection to improve overall survival. Unfortunately, radiotherapy resistance is a frequently observed phenomenon in affected patients. The mechanisms underlying the intrinsic radio-resistance in GBM are multifactorial, although altered DNA damage response seems to be the most crucial operator in the outcome to IR exposure. In the present work we are investigating the effectiveness of a novel approach to radio-sensitize GBM cells through the use of gold nanoparticles (AuNPs). AuNPs are promising radio-sensitizing agents due to their high biocompatibility and ability to be synthesized with various shapes and structures. AuNPs act by photothermal therapy (PTT), an efficient method of inducing localized hyperthermia aiming to selectively kill tumor cells. In this work, AuNPs, specifically nanoprisms (NPrs), have been tested in two GBM cell lines: U87MG stabilized cell line and a primary cell line named GBM3. Preliminary data show that AuNPrs alone at low concentrations have no toxic effects in both GBM cell lines used, where AuNPrs demonstrated an efficient cytosolic internalization. More importantly, the combination of AuNPrs with increasing IR doses (2Gy-8Gy) showed a greater reduction in cellular viability and colony formation when compared with samples treated with IR alone. This suggests that throughout thermoablation, AuNPrs are able to weaken cells thus making them more susceptible to lower doses of IR. Combination therapy based on AuNPrs and subsequent low-dose IR could be considered a promising alternative to standard GBM treatment involving much higher IR doses (60Gy).

NO06 | Astrocytes-derived small extracellular vesicles hinder glioma growth by the regulation of the volume-regulated anion channel (VRAC)

Mariassunta De Luca [(1)] - Carmela Serpe [(1)] - Lucia Monaco [(1)] - Arianna Rinaldi [(1)] - Igea D'Agnano [(2)] - Cristina Limatola [(1)] - Myriam Catalano [(1)]

Sapienza University of Rome, Department of Physiology and Pharmacology, Rome, Italy (1) - CNR - Centro Nazionale delle RIcerche, Institute of Biomedical Technologies, Segrate (MI), Italy (2)

Small extracellular vesicles (sEVs) represent a way used from all cells of the body, including brain, to exchange biological information (lipids, proteins and nucleic acids as mRNA, miRNA, ctDNA) during neural functional processes but also in pathological conditions (such as inflammation, neurodiseases or brain cancer).
sEVs mediate a bidirectional crosstalk between healthy and cancer cells in the most common and malignant primary brain tumor, the glioblastoma (GBM). GBM has a high rate of invasiveness, migration and chemoresistance. In the peri-tumoral environment, astrocytes act as pro-tumoral or antitumoral cells depending on the stage of GBM progression.
In this study, we demonstrated that astrocytes-derived sEVs (ADEVs) have a defensive mechanism against tumor invasion and cell growth. sEVs response is mediated by the transfer to tumor cells of factors that hinder glioma growth, reducing both tumor volume and tumor cell proliferation and prolonging survival of glioma-bearing mice. Among many factors transported by ADEVs, we identified miR124 that is enriched in these vesicles. Among downregulated target genes of miR124, we found the volume-regulated anion channels (VRACs). VRACs regulate GBM cell migration and invasion. We demonstrated that ADEVs reduce migration and invasion of GBM cells by translocating miR124.
In summary, astrocytes exert an anti-tumor response in the context of GBM by releasing ADEVs enriched in miR124.

NO08 | Phospholipases as potential prognostic biomarkers and targets in the development of new therapeutic strategies for glioblastoma

Maria Vittoria Marvi [(1)] - Isabella Rusciano [(1)] - Sara Mongiorgi [(1)] - Irene Neri [(1)] - Lucia Manzoli [(1)] - Lucio Cocco [(1)] - Nikos Tapinos [(2)] - Stefano Ratti [(1)]

Cellular Signalling Laboratory, Department of Biomedical and Neuromotor Sciences, University of Bologna, Bologna, Italy (1) - Laboratory of Cancer Epigenetics and Plasticity, Brown University, Rhode Island Hospital, Providence, United States (2)

Phospholipases (PLCs) are the hydrolyzing enzymes of phospholipids, which represent the most abundant species contributing to the biological membranes of nervous cells of the healthy human brain. Several studies have shown the importance of PLCs in the regulation of different mechanisms in the central nervous system as well as in glioblastoma, the most lethal brain tumor in adults. Nowadays, despite the progress made in understanding the molecular pathogenesis of glioblastoma, the survival rate of patients remains unsatisfactory. Consequently, a better understanding of the molecular mechanisms underlying tumor transformation could help to find new effective therapeutic strategies. Our studies suggested a potential role of PLCβ1 and PLCg1, in regulating the phenotypic characteristics of this tumor. It was demonstrated that PLCβ1 expression was relatively lower in glioblastoma patients compared to their healthy/low-grade counterparts. PLCβ1 silencing, in both immortalized and primary cell lines, led to increased cell migration, invasion, proliferation, cell survival and induced the upregulation of mesenchymal markers and metalloproteinases. Contrariwise, data collected on patients' biopsies and engineered cell models, proposed a strong association between PLCγ1 expression and the acquisition of a more aggressive tumor phenotype. This trend was deepened using patient derived glioma stem cells (GSCs), which represent a specific tumor population that drives aggressiveness and recurrence in glioblastoma. Transcriptomic analysis of GSCs confirmed that PLCγ1 downregulation led to the activation of pathways that negatively regulate cell motility and migration and led to reduced expression of genes involved in cancer development and progression. All these data highlight the importance of further investigating phospholipases as potential prognostic biomarkers and targets in the development of new therapeutic strategies for glioblastoma.

NO10 | The use of animal proteins in the diet: impact on gut microbiota and glioma growth in a preclinical murine model

Alice Reccagni [(1)] - Xingzi Lin [(1)] - Cristina Limatola [(1)] - Giuseppina D'Alessandro [(1)]

University La Sapienza, Dipartimento di Fisiologia e Farmacologia, Roma, Italy (1)

Glioblastoma multiforme (GBM) is the most common and deadly malignant brain tumor, with a low life expectancy (around 14-17 months) and poor efficacy of first-line therapies such as maximal surgical resection and chemotherapy, leading to a high relapse rate. However, it is already known that lifestyle changes, such as restricted diets and fasting, have an impact on GBM tumor growth. Less clear is the effect of eating red meat on the tumor. One observational study found that people who consume a diet high in animal protein and fat have changes in the composition of their microbiota, in particular an increase in common hydrogen sulfide (H_2S) producing bacteria. This metabolite appears to counteract GBM growth in both in vitro and in vivo models. Recent studies have highlighted the role of the gut-brain axis in altering the GBM tumor microenvironment and growth. Our aim is to investigate the effect of a standard animal protein diet on tumor growth by the possible involvement of the gut microbiota. To assess such a hypothesis, we fed mice two isocaloric diets with different percentages of proteins derived from red meat (protein diets) or animal-derived proteins (control). After two weeks on the diets, we orthotopically injected murine glioma cells (GL261). We allowed the tumors to grow for a further three weeks. At the end of the experiment, we collected stool for each group to assess H_2S concentration and brains to assess tumor volume. Preliminary data showed an increased concentration of H_2S in the faeces and a decreased tumor volume in mice fed the animal protein diet compared to controls. These results are consistent with our in vitro data showing a reduction in GL261 viability following treatment with two doses of sodium hydrosulfide (NaHS), an H_2S donor. These preliminary results suggest that a standard red meat diet may have an antitumor effect on GBM compared to a standard animal-derived protein diet.

NO11 | Identification of a Novel KDM5C-Related Signature in Glioblastoma Multiforme

Denise Drongitis (1) - Lucia Verrillo (1) - Martina Schiano Visconte (1) - Pasqualino De Marinis (2) - Valerio Costa (1) - Alberto de Bellis (2) - MariaGiuseppina Miano (1)

CNR, Institute of Genetics and Biophysics Adriano Buzzati-Traverso, Naples, Italy (1) - A.O.R.N. S. Anna and S. Sebastiano Hospital, Division of Neurosurgery, Caserta, Italy (2)

Glioblastoma multiforme (GBM) is the most aggressive brain tumour without an effective pharmacological treatment. We analyzed the expression levels of the cancer driver gene Lysine (K)-specific demethylase 5C (KDM5C) in a series of GBM tissues. KDM5C belongs to the Jumonji C domain-containing histone demethylase family involved in various cancer types. It catalyzes the removal of the methyl groups from di- and tri-methylated lysine 4 on histone H3 in a Fe (II)- and α-ketoglutarate-dependent manner. By using real-time quantitative PCR and Western blotting analysis, we found an altered abundance of KDM5C transcript and protein in GBM samples identifying patients with higher (KDM5CHigh) and lower (KDM5CLow) levels compared to control samples. By exploring the impact of the defective KDM5C quantity, a positive and negative relationship with hypoxia -inducible transcription factor-1α (HIF-1α) and BDNF levels were found in $KDM5C^{High}$ patients. KDM5C overexpression and hypoxic studies performed in glioblastoma cell line (T98G) suggest that the stimulation of KDM5C expression is preceded by the induction of HIF-1α. High levels of HIF1α-KDM5C axis was also found associated with high levels of NANOG, SOX2 and NESTIN in GBM tissues isolated from conventional and 5-aminoleveulinic acid (5-ALA) fluorescence-guided surgery (FGS). A pro-inflammatory condition was also detected in 5-ALA FGS highlighting differences across the GBM microenvironment. Taken together, our study reveals for the first time a correlation between the HIF-1α-KDM5C axis and GBM opening a new field of investigation to validate KDM5C as a new GBM biomarker.

ND03 | Betaine is a substrate of GAT1 that can modulate extracellular GABA

Manan Bhatt [1,2,4] - Guido Domingo [1] - Erika Lazzarin [3,4] - Angela Di Iacovo [1,2] - Chiara D'agostino [1,2] - Candida Vanini [1] - Cristina Roseti [1,2] - Thomas Stockner [3,4] - Elena Bossi [1,2,4]

Department of Lifesciences and Biotechnology, University of Insubria, Italy (1) - Centre of Neuroscience, University of Insubria, Italy (2) - Institute of Pharmacology, Medical University of Vienna, Austria (3) - NeuroTrans MSCA ITN (4)

Betaine, N,N,N-trimethyl glycine, is an osmolyte that shows ameliorating effects in neurological and neurodegenerative diseases like Alzheimer's, Parkinson's, schizophrenia. While there has been an ongoing surge of the studies demonstrating positive effects of betaine, the molecular mechanism of action and the translocation process is still not clear. The betaine/ γ-aminobutyric acid (GABA) transporter 1 (BGT-1, slc6a12) and the sodium-coupled neutral amino acid transporter 2 (SNAT2, slc38a2) can transport betaine in the brain, however due to their little expression, it has been suggested that betaine should be interacting with proteins other than these, especially across the GABAergic pathways.
In this work, we show that GAT1 (slc6a1), the most expressed GABA transporter in the central nervous system (CNS), can translocate betaine across the neuronal membrane, though with lower affinity ($K_{0.5}$≈11mM at -60mV) than GABA. Using electrophysiological experiments on *Xenopus laevis* oocytes heterologously expressing rGAT1, we demonstrate that betaine induces inward transport currents, which are dose-, voltage-, and Na^+ dependent. The betaine transport can be blocked by GAT1 specific inhibitor like SKF89976a. We also confirmed that betaine is a substrate of GAT1 using molecular docking, radiolabelled release assay in HEK293 cells, and LCMS-MS technique. More interestingly, our results on GABA-betaine relationship for GAT1 show that betaine at µM concentration can effectively modulate the GABA transport. It inhibits the transport when the extracellular GABA concentration is less than its $K_{0.5}$ (≈16 µM at -60mV), while at higher extracellular GABA concentration, betaine behaves like a secondary substrate. Uptake data obtained by LCMS-MS detection of GABA and betaine inside the oocytes expressing rGAT1, provide direct supporting evidence for the same phenomena. This modulatory behaviour of betaine on GABA transport suggests a role of neuromodulator in maintaining GABA homeostasis and excitatory/inhibitory balance in the CNS.

ND02 | A potential therapeutic strategy for CMT2A: combined RNA interference and gene therapy in vitro and in vivo disease models

Alessia Anastasia (1) - Linda Ottoboni (2) - SIlvia Bono (1) - Sabrina Salani (1) - Valentina Melzi (1) - Serena Pagliarani (1) - Elena Abati (2) - Giacomo Comi (1) - Stefania Corti (2) - Federica Rizzo (1)

Foundation IRCCS Ca' Granda Ospedale Maggiore Policlinico, Neurology Unit, Milan, Italy (1) - University of Milan, Department of Pathophysiology and Transplantation, Dino Ferrari Centre, Milan, Italy (2)

CMT2A is an autosomal dominantly inherited disease characterized by progressive muscle weakness, and atrophy, loss of sensation and motor difficulties mainly in the distal limbs. It is caused by missense mutations in the *Mitofusin2 (MFN2)* gene which induce the disease with a dominant negative mechanism, negatively regulating the wild-type MFN2 allele expression. Gene therapy for dominant inherited diseases uses RNA interference (RNAi) to selectively inhibit expression of the mutant allele, which results in a toxic protein. Since this approach can also reduce the expression of the wild-type functional allele, wild-type allele restoration, in combination with mutant allele silencing, could improve the therapeutic effects. Here, we propose this novel potential therapeutic approach combining RNAi and gene therapy, whereby mutant and wild-type *MFN2* mRNA are inhibited by RNAi, while the wild-type protein is restored by overexpressing cDNA encoding functional MFN2 modified to be resistant to RNAi. First, we tested the effective silence of the endogenous MFN2 (both mutant and wild-type *MFN2* alleles) and its replacement with an exogenous copy of the wild-type *MFN2* gene in CMT2A human induced pluripotent stem cells (iPSCs)-differentiated motor neurons, confirming the molecular efficacy of our strategy. To evaluate the amelioration of the disease phenotype, we analysed key motoneuronal features relevant to CMT2A, observing an enhancement in mitochondrial distribution and function, beyond in apoptotic and autophagic parameters in CMT2A MNs. We, also, assessed the molecular efficacy in Mitocharc1 CMT2A mouse model after cerebrospinal fluid (CSF) delivery of the constructs into newborn mice using adeno-associated virus 9 (AAV9). Our data confirm the feasibility of combined RNAi and gene therapy approach as potential therapeutic strategy for treating CMT2A and other similar genetic neurological disorders even if the therapeutical efficacy need to be validated in vivo.

ND08 | Intramuscular IL-10 Administration Enhances the Activity of Myogenic Precursor Cells and Improves Motor Function in ALS Mouse Model

Cassandra Margotta [(1)] - Paola Fabbrizio [(1)] - Jessica D'Agostino [(1)] - Giuseppe Suanno [(1)] - Lorenzo Quetti [(1)] - Caterina Bendotti [(1)] - Giovanni Nardo [(1)]

Institute for Pharmacological Research Mario Negri, Department of Neuroscience, Milan, Italy (1)

Amyotrophic Lateral Sclerosis (ALS) is the most common adult motor neuron disease, with a poor prognosis, a highly unmet therapeutic need, and a burden on health care costs. Hitherto, strategies aimed at protecting motor neurons have missed or modestly delayed ALS due to a failure in countering the irreversible muscular atrophy. We recently provided direct evidence underlying the pivotal role of macrophages in preserving skeletal muscle mass.
Based on these results, we explored whether the modulation of macrophage muscle response and the enhancement of satellite cell differentiation could effectively promote the generation of new myofibers and counteract muscle dysfunction in ALS mice. For this purpose, disease progression and the survival of SOD1G93A mice were evaluated following IL-10 injections in the hindlimb skeletal muscles. Thereafter, we used ex vivo methodologies and in vitro approaches on primary cells to assess the effect of the treatment on the main pathological signatures.
We found that IL-10 improved the motor performance of ALS mice by enhancing satellite cells and the muscle pro-regenerative activity of macrophages. This resulted in delayed muscle atrophy and motor neuron loss. Our findings provide the basis for a suitable adjunct multisystem therapeutic approach that pinpoints a primary role of muscle pathology in ALS.

ND12 | An insight into Alzheimer's disease pathogenesis: cell-laden hydrogels and oxidative stress

Valentina Peluso [(1)] - Stefania Scala [(2)] - Roberto De Santis [(1)] - Antonio Gloria [(3)] - Teresa Russo [(1)]

National Research Council of Italy, Institute of Polymers, Composites and Biomaterials, Naples, Italy (1) - University of Naples Federico II, Department of Biology, Naples, Italy (2) - University of Naples Federico II, Department of Industrial Engineering, Naples, Italy (3)

Alzheimer's disease (AD) is a progressive neurodegenerative disorder leading to the most common form of dementia in elderly people. Over time, a person with Alzheimer's gradually loses his/her ability to live independently. Research on the pathogenesis of AD is mainly focused on the amyloid cascade, tau protein, neuroinflammation, metal ions, and oxidative stress hypotheses. Oxidative stress is a process that causes neuronal damage and occurs in various pathways, acting as a bridge between the different hypotheses and mechanisms of AD. Meanwhile, nanocomposite and nanostructured hydrogels, functionalized for drug and cell delivery, offer a variety of possible cutting-edge scenarios such as tissue repairing and bioinspired scaffolds, extracellular matrix analogues, exploiting their good biocompatibility. The aim of this study was to develop a novel *in vitro* model based on co-culture of MG63/SHSY5Y cell lines embedded in different formulations of collagen and hyaluronic acid-based semi-IPNs. Alamar Blue assay and western blot analysis were performed to investigate the differentiative and proliferative capacities of the proposed model after oxidative stress. Moreover, Confocal Laser Scanning Microscopy has allowed to evaluate the capability to improve the expression of neurological differentiation markers for the co-culture system, if compared to single culture system. Results showed an improved expression of Neuron Specific Enolase(NSE) in co-culture systems in comparison to single culture system. The obtained results support a biomaterials-based approach for controlled delivery of cell-produced neuroprotective factors in AD experimental context. The authors thank PRIN2017 2017XKJTLW_003 - "EXPLOITATION OF CIRCULATING MIRNAS FOR DIAGNOSIS AND NEUROPROTECTION IN TRANSLATIONAL STROKE STUDIES" and PRIN 2020WREYF2 "3D Customized HYbrid MedicAl Devices for Alzheimer's disease-related Periodontitis Treatment - 3D CHYM ADAPT" for providing the financial support to this work.

ND13 | In vitro evaluation of the antioxidant capacity of novel benzofuran-2-ones in a cellular model of neurodegeneration

Sofia Scibetta (1) - Martina Miceli (2) - Marco Iuliano (1) - Luca Stefanuto (2) - Giovanna Romeo (1) - Antonella Calogero (1) - Giorgio Mangino (1) - Tecla Gasperi (2) - Paolo Rosa (1)

Sapienza, Department of Medical-Surgical Sciences and Biotechnologies, Latina, Polo Pontino, Italy (1) - Roma Tre, Department of Science, Section of Nanoscience and Nanotechnology, Rome, Italy (2)

Neurodegenerative diseases (NDs) are characterized by the progressive loss of neurons in the Central Nervous System (CNS). In the healthy brain, oxidative stress (OS) is well counterbalanced by antioxidative defences. The augmented OS is generally associated with neuronal loss, a typical hallmark of several NDs. A pivotal role in the pathogenesis and progression of NDs is played by heme oxygenase-1 (HO-1), a 32 kDa heat-shock protein which may be protective or toxic, depending on its levels of expression. Therefore, it becomes of fundamental importance to consider antioxidants (AOs) as an adjuvant strategy to control cellular OS within the CNS.

The aim of this study was to investigate the antioxidant activity of four newly synthesized benzofuran-2-one derivatives in differentiated SH-SY5Y cells exposed to catechol-induced OS.

Our results show that, upon phorbol 12-myristate 13-acetate (PMA) differentiation, SH-SY5Y cells were more sensitive to OS than the undifferentiated counterpart. Among the tested AOs, compounds 1 and 4 significantly reduced catechol-induced cell death, being the AO 4 the most effective. Moreover, dichlorofluorescin diacetate (DCFH-DA) assay confirmed that both molecules can reduce intracellular ROS concentration in our cellular model in a greater extent than Trolox, a reference AO. Further, western blot analysis showed that catechol induced high levels of HO-1, thus confirming toxic effects on cells, and that AOs 1 and 4 can limit this induction. Additionally, we have evaluated the efficacy of the AOs in an *in vitro* model of microglia/neuron interplay by treating SH-SY5Y cells with supernatants collected from LPS/IFN-γ activated THP-1 monocytes. In this condition, only AO 4 showed an appreciable antioxidant capacity.

In conclusion, the newly synthesized benzofuran-2-ones tested in this work could represent promising molecules to contrast OS, opening the way for new adjuvant strategies that might improve the life quality of patients with NDs.

ND14 | Alteration of lipid metabolism in the pathogenesis of Hereditary Spastic Paraplegia: unraveling the mechanisms to recover cell function

Sonia Sonda (1) - Ambra Bertocco (1) - Manuela Santalla (2) - Alberto Ongaro (3) - Manuela Simonato (4) - Andrea Mattarei (3) - Diana Pendin (2)

University of Padova, Department of Biomedical Sciences, Padova, Italy (1) - National Research Council, Neuroscience Institute, Padova, Italy (2) - University of Padova, Department of Pharmaceutical and Pharmacological Sciences, Padova, Italy (3) - Fondazione Istituto di Ricerca Pediatrica "Città della Speranza", Pcare laboratory, Padova, Italy (4)

Hereditary Spastic Paraplegias (HSPs) are a group of inherited neurologic disorders in which lower extremity weakness and spasticity are the predominant symptoms. HSPs are characterized by high genetic heterogeneity. Nevertheless, alterations in morphology or distribution of the Endoplasmic Reticulum (ER) appear to be a critical pathogenic factor. Mutations in two genes encoding crucial enzymes to the plasmalogens (PLs) biosynthetic pathway, *i.e.*, SPG81 and SPG82, have been recently identified in HSP patients. PLs are ether phospholipids abundant in ER membranes. Ethanolamine-based PLs (PE-PLs) are enriched in nervous system membranes, constituting up to 85 mol% of total phosphatidylethanolamine (PE) species and up to 30 mol% of total phospholipids in mammalian brains. Notably, PLs amount was found decreased in several neurological diseases, suggesting that PLs could play a role in neuronal membranes welfare. PE-PLs are suggested to promote the formation of inverted hexagonal phases, thus facilitating membrane fusion events. However, the sub-molecular details behind the above properties are not fully understood. We aim at identifying a potential role for PLs in the remodeling of ER membranes. Our hypothesis is that manipulating ER membrane lipid composition in a way that favors membrane dynamics, we could rescue HSP-related ER morphology defects. Our goal is to test if administration of bioavailable PLs precursors to validated HSP fly models is able to increase the amount of membrane PLs, and to improve HSP-related phenotypes (birth rate, survival rate, and locomotor ability). The validated approach could prove a new therapeutic option for HSPs and potentially for other neurodegenerative diseases involving phospholipid-related membrane impairment.

ND18 | Identification of a Novel Class of Small Molecules for the Treatment of TREM2-Based Diseases

Carmela Gallo (1) - Lucia Verrillo (2) - Maria Giuseppina Miano (2) - Emiliano Manzo (1) - Giusi Barra (1) - Mario Dell''Isola (1) - Mario Affuso (3) - Dalila Carbone (1) - Marcello Ziaco (1) - Laura Fioretto (1) - Angelo Fontana (1)

National Research Council, Institute of Biomolecular Chemistry, Pozzuoli, Italy (1) - National Research Council, Institute of Genetics and Biophysics, Napoli, Italy (2) - University of Naples Federico II, Department of Biology, Napoli, Italy (3)

The triggering receptor expressed on myeloid cells 2 (TREM2) is a member of immunoglobulin superfamily mainly expressed by microglia. This receptor promotes microglial proliferation and survival, as well as regulating phagocytosis and metabolism, and is proposed to mediate a novel form of microglial anti-inflammatory activation. A number of TREM2 variants have been identified as risk factors for a wide array of neurodegenerative diseases (NDs), including Nasu-Hakola disease, Alzheimer's disease and Parkinson disease (PD). In AD-related conditions, TREM2 interacts with lipoproteins, anionic lipids, and Aβ, which contributes to microglial metabolism remodelling, as well as promotes microglial phagocytosis of cell debris and Aβ. These effects support the hypothesis that TREM2 might play a protective role through regulating microglia polarization and be a potential target for AD prevention and treatment.
Many proteins or compounds that bind to TREM2 have been reported, but the natural signal-transducing ligands of TREM2 present in the brain have not been identified. We recently reported a novel immunomodulatory sulfolipid named Sulfavant A (SULF-A), which primes maturation of dendritic cells (DC) towards a novel homeostasis-determining phenotype (homeDCs) by engagement of TREM2. Preliminary results suggested the ability of this molecule to activate also microglial cells towards an unconventional non inflammatory state, prompting the idea that the investigation of TREM2 pathway may lay the groundwork for the development of a new class of drugs with therapeutic potential in neurodegenerative diseases, chronic-inflammation and cancer.

ND19 | The role of G2019S LRRK2 in excitatory/inhibitory imbalance of Parkinson's disease

Angela Di Iacovo [(1)] - Ludovica Iovino [(2)] - Mattia Vimercati [(1)] - Manan Bhatt [(1)] - Raffaella Cinquetti [(1)] - Laura Civiero [(2)] - Elena Bossi [(1)] - Cristina Roseti [(3)]

University of Insubria, Department of Biotechnology and Life Sciences, Varese, Italy (1) - University of Padua, Department of Biology, Padova, Italy (2) - Universitt of Insubria, Department of Biotechnology and Life Sciences, Varese, Italy (3)

The excitatory/inhibitory (E/I) balance of neural circuits is tightly regulated by an appropriate ratio of excitatory to inhibitory synaptic signals, which falls in several neurodevelopmental and neurodegenerative disorders. Parkinson's disease (PD) is associated with general modifications in the circuitry, resulting in E/I imbalance in the striatum caused by an aberrant excitatory input responsible for the excitotoxicity phenomenon. Recently, *Leucine-rich repeat kinase 2* (LRRK2) has been discovered to play a role in both monogenic and sporadic forms of PD, in which the substitution Gly2019Ser has been observed at a high frequency. It has been demonstrated that G2019S LRRK2 takes part in glutamatergic reuptake process by regulating the activity of glutamate transporter EAAT2 and its membrane localization. On the contrary, the role of LRRK2 on GABAergic transmission is poorly understood. By two-electrode voltage-clamp technique, we investigated the possible modulation of LRRK2 on inhibitory neurotransmission. Taking advantage of microtransplantation technique, striatal membranes derived from G2019S LRRK2-associated mouse model were be injected in *Xenopus laevis* oocytes.

Our data showed a significant reduction of GABA evoked current amplitude in G2019S LRRK2 striatum compared to the wild-type tissue, indicating that LRRK2 affects GABAergic transmission. The reason behind this reduction is still unclear as the $GABA_A$ receptors are functionally unaltered. Notably, our findings suggest an altered distribution of different $GABA_A$ receptor isoforms in G2019S LRRK2 striatum membrane compared to the wild-type counterpart, demonstrated by both slower desensitization of $GABA_A$ receptor and reduced phasic GABA current.

Overall, our results highlight a critical role of LRRK2 on GABAergic signalling, participating in E/I imbalance of PD LRRK2-associated.

ND20 | Effect of 3D Synthetic Microscaffold Nichoid on the Morphology of Cultured Hippocampal Neurons and Astrocytes

Arianna Giani [1] - Clara Alice Musi [1] - Luca Colnaghi [2] - Erica Cecilia Priori [1] - Matteo Tironi [3] - Claudio Conci [4] - Giulio Cerullo [5] - Roberto Osellame [5] - Manuela Teresa Raimondi [4] - Andrea Remuzzi [6] - Tiziana Borsello [1]

Università degli Studi di Milano, Department of Pharmacological and Biomolecular Sciences, Milan, Italy (1) - IRCCS San Raffaele Scientific Institute, Division of Neuroscience, Milan, Italy (2) - Mario Negri Institute for Pharmacolgical Research—IRCCS, Department of Neuroscience, Milan, Italy (3) - Politecnico di Milano, Department of Chemistry, Materials and Chemical Engineering "G. Natta", Milan, Italy (4) - Politecnico di Milano, Department of Physics, Istituto di Fotonica e Nanotecnologie (IFN)-CNR, Milan, Italy (5) - University of Bergamo, Department of Management, Information and Production Engineering, Bergamo, Italy (6)

The human brain is the most complex organ in biology, being composed of an extraordinary number of synapses. Considering that the brain pathologies are bound to rise, there is an essential need to establish effective in-vitro system of Central Nervous System that could be applied to test new therapeutic avenues. To this aim, we set up a new model of hippocampal neurons and astrocytes co-culture taking advantage of the Nichoid technology, a 3D scaffold microfabricated by two photon laser polymerization technology, to generate brain micro-tissues of 30 µm thickness. After 21 days in-vitro, by confocal microscopy, we morphologically characterized the co-cultures comparing 2D and 3D conditions. We observed that astrocytes as well as and neurons had become well-differentiated and colonized the entire volume of the Nichoid. This was further elaborated with the use of Drebrin, PSD-95, and Synaptophysin antibodies that labelled most neurons, both in the 2D as well as in the 3D co-cultures. Interestingly, in the Nichoid, astrocytes displayed a more physiological morphology, closer to the in-vivo condition, appearing more starry compared to 2D cultures. Lastly, using Scanning Electron Microscopy, we found that neurons co-cultured with astrocytes in the 3D environment showed more dendritic spine protrusions compared to the 2D culture, suggesting they could be more prone to form connections.

Our results show that the Nichoid can be used as a 3D device to investigate the structure and morphology of neurons and astrocytes in-vitro as well as the complex cell-cell interactions within the brain. In addition, it may serve as a tool to study mechanisms governing synaptic plasticity/dysfunction and to drug discovery.

ND22 | What about astrocytes? Elucidating the new role of astrocytes in Autosomal Dominant Leukodystrophy

Foteini-Dionysia Koufi (1) - Isabella Rusciano (1) - Giulia Ramazzotti (1) - Sara Mongiorgi (1) - Pietro Cortelli (2) - Elisa Giorgio (3) - Marianna Bugiani (4) - Lucia Manzoli (1) - Stefano Ratti (1)

Cellular Signaling Laboratory, University of Bologna, Department of Biomedical and Neuromotor Sciences, Bologna, Italy (1) - University of Bologna, Department of Biomedical and Neuromotor Sciences, Bologna, Italy (2) - University of Pavia, Department of Molecular Medicine, Pavia, Italy (3) - Amsterdam University Medical Centers, Department of Pathology, Amsterdam, Netherlands (4)

Autosomal Dominant Leukodystrophy (ADLD) is an ultra-rare and fatal late-onset neurodegenerative disorder that affects the central nervous system myelination and lacks effective therapy. The disease is caused by lamin B1 (*LMNB1*) gene alteration that leads to demyelination with the disease mechanisms remaining unknown. Although oligodendrocytes are responsible for myelination, astrocytes and ADLD patients' cells overexpressing *LMNB1* have displayed nuclear alterations with activation of proinflammatory and oxidative stress mechanisms that were absent in oligodendrocytes. The present study involved the characterization of astrocytes overexpressing lamin B1 and the elucidation of their new role in demyelination. Human astrocytes (HA) were transfected with *LMNB1* and sorted for two assays: 1) characterization of astrocytes for expression of proinflammatory markers, and 2) myelination assay on 3D microfiber co-cultures with oligodendrocyte precursor cells (OPCs). For the characterization, immunocytochemical analysis displayed nuclear localization of NFAT4 (nuclear factor of activated T cells 4) and NF-κB (nuclear factor kappa-light-chain-enhancer of activated B cells) suggesting astrocytic activation and inflammation. Additionally, proteome array of the transfected HA supernatants revealed increased levels of serpin E1, osteopontin and Dkk-1 which are markers present in activated astrocytes during CNS injury. For the myelination assay the sorted HA were co-cultured with human OPCs on a microfiber scaffold for two weeks. It was displayed that OPCs were unable to produce myelin basic protein when grown with HA overexpressing *LMNB1* indicating the crucial role of astrocytes in supporting myelination. Overall, the study elucidated that *LMNB1* overexpression leads to astrocyte activation that consequently triggers inflammatory states and hinders myelination. Thus, these novel findings could place astrocytes at the epicenter of ADLD demyelination and drug development studies.

ND16 | Brain organoids: a promising approach to investigate neuro-degeneration in MSA-C and SCA2

Lorenzo Brambilla [(1)] - Mafalda Rizzuti [(1)] - Valentina Melzi [(1)] - Sabrina Salani [(1)] - Jessica Ongaro [(1)] - Noemi Galli [(2)] - Linda Ottoboni [(2)] - Stefania Magri [(3)] - Caterina Mariotti [(3)] - Chiara Cordiglieri [(4)] - Giacomo Pietro Comi [(1)] - Franco Taroni [(3)] - Stefania Corti [(2)]

Foundation IRCCS Ca' Granda, Ospedale Maggiore Policlinico, Milan, Italy (1) - University of Milan, DEPT, Milan, Italy (2) - Foundation IRCCS Istituto Neurologico Carlo Besta, U.O.C. Genetica Medica e Neurogenetica, Milan, Italy (3) - Istituto Nazionale di Genetica Molecolare Romeo ed Enrica Invernizzi, Imaging facility, Milan, Italy (4)

The complexity of the human brain has made the study of brain disorders difficult, thus highlighting the need to generate *in vitro* models for human brain degeneration to overcome the limitations of *in vivo* animal studies. The generation of a three-dimensional model is one of the most promising approaches to study human diseases of the central nervous system (CNS), as it provides robust and consistent phenotypes with clinical translatability. Here, we take advantage of optimized iPSC-derived cortical organoids to study the pathological mechanisms underlying two rare neurological diseases, Multiple System Atrophy Cerebellar type (MSA-C) and Spinocerebellar Ataxia type 2 (SCA2), associated with the expansion of Ataxin-2. First, we generated iPSC lines for MSA-C subjects (n=3), age-matched controls (n=3), SCA2 individuals (n=3) and respective isogenic controls. Further, we cultured and deeply characterized via immunofluorescence, RT-PCR, calcium imaging and electrophysiology cortical organoids derived from subject-specific iPSCs lines. Finally, we also explored the use of antisense oligonucleotides (ASOs) designed with Morpholino chemistry to modulate the expansion of *ATXN2*. Indeed, although there are currently no approved disease-modifying treatments for these two diseases, RNA-targeted therapies are promising for neurodegenerative disorders. Regarding SCA2, creating new reliable human models may help optimize this antisense therapeutic strategy. On the other hand, the pathogenesis of MSA is still puzzling, and a comparison of data obtained in 3D models of MSA-C with those from SCA2 may provide insights into understanding the pathogenesis of this complex disorder.

ND26 | Intensive exercise training counteracts nigrostriatal degeneration and striatal structural changes in an alpha-synuclein based experimental model of Parkinson's disease

Federica Servillo [1] - Maria De Carluccio [1,2] - Gioia Marino [1] - Federica Campanelli [1] - Giuseppina Natale [1] - Veronica Ghiglieri [1,3] - Maria Teresa Viscomi [1,4] - Paolo Calabresi [1,4]

Facoltà di Medicina e Chirurgia, Università Cattolica del Sacro Cuore, Rome, Italy (1) - IRCCS San Raffaele Roma, Roma, Italy (2) - Department of Human Sciences and Quality of Life Promotion, Università Telematica San Raffaele, Rome, Italy (3) - Fondazione Policlinico Universitario Agostino Gemelli IRCCS, Rome, Italy (4)

Parkinson's disease (PD) is characterized by Lewy-body aggregates formation, and dopaminergic neuronal loss. Clinically, PD is associated with motor slowing, rigidity, and tremor. Intensive physical exercise has beneficial effects on PD patients at early stages, but the underlying mechanisms are not completely understood. In this study, we investigated whether an intensive treadmill training program, at an early phase, counteracts nigrostriatal neurodegeneration in intrastriatal alpha-synuclein (α-syn) preformed-fibrils (PFFs)-injected rats. To evaluate the effects of treadmill on nigrostriatal morpho-functional changes, we assessed the dopaminergic neurons survival, counting tyrosine hydroxylase (TH)-positive neurons of the Substantia Nigra *pars compacta* (SNpc); and the functional integrity of their striatal terminals, by dopamine active transporter (DAT) expression level. Furthermore, we analyzed the dendritic spine density of the striatal spiny projection neurons. Comparisons were made between sedentary and active α-syn-PFFs-injected animals compared to control animals. Interestingly, in active parkinsonian animals we found increased number of SNpc neurons with a higher density of DAT+ - terminal-fibers in the dorsolateral-striatum, compared to sedentary α-syn-PFF-injected rats. Furthermore, active animals showed increased spine density in striatal projection neurons with a greater proportion of young immature spines. These structural changes were also associated with a functional outcome as active animals displayed better performances in motor coordination and visuospatial learning tests. In conclusion, we demonstrate that intensive exercise training in parkinsonian animals, at presymptomatic stages of disease, has effects in counteracting neurodegeneration of the nigrostriatal pathway. Physical activity induces striatal adaptive responses to the α-syn-PFFs intoxication, associated with improved motor and cognitive performances compared to the parkinsonian sedentary group.

ND27 | Neuroprotective activity of the new metabotropic Glutamate Receptor 3 Positive Allosteric Modulator in Parkinson's Disease in vitro and in vivo models

Giulia Urone [(1)] - Monica Frinchi [(1)] - Miriana Scordino [(1)] - Virginia Spanò [(2)] - Paola Barraja [(2)] - Giuseppa Mudò [(1)] - Valentina Di Liberto [(1)]

University of Palermo, Department of Biomedicine, Neuroscience and Advanced Diagnostic, Palermo, Italy (1) - University of Palermo, Department of Biological, Chemical and Pharmaceutical Sciences and Technologies, Palermo, Italy (2)

Parkinson's Disease (PD) is a complex and progressive neurodegenerative disorder associated with the gradual loss of dopaminergic neurons in the nigrostriatal circuit, which leads to the development of motor dysfunctions, such as rest tremors and rigidity, and non-motor symptoms, including dementia. Currently, no disease-modifying pharmacological treatments are available, and dopamine-based therapies typically help to mitigate symptoms but also cause side-effects such as dyskinesia. In this context, indirect pieces of evidence have suggested that activation of metabotropic Glutamate Receptor 3 (mGluR3) is able to exert neuroprotective effects in animal models of PD. However, so far, the lack of selective agonists/ligands for this receptor has hindered more in-depth investigations. Recently, a new Positive Allosteric Modulator (PAM) selective for mGluR3 has been synthesized. In this study, we demonstrate that the novel mGluR3 PAM is able to protect SH-SY5Y cells, a human neuroblastoma cell line, against the degeneration induced by 6-hydroxydopamine (6-OHDA) exposure through the modulation of Mitogen-activated protein kinases/extracellular signal-regulated kinase (MAPK/ERK) and phosphatidylinositol 3-kinase (PI3K)-Akt signaling pathways. Furthermore, *in vivo* treatment with mGluR3 PAM up-regulates the expression of Glial cell line-derived neurotrophic factor (GDNF) and Brain-derived neurotrophic factor (BDNF), and modulates the activation of MAPK/ERK and PI3K-Akt pathways in several mouse brain regions. Overall, though preliminary, our results suggest the therapeutic potential of the new mGluR3 PAM in the context of PD management.

ND28 | Investigating the role of large microglial extracellular vesicles carrying pathogenic misfolded proteins in Alzheimer's disease and their interaction with neurons

Elisabetta Battocchio (1) - Martina Gabrielli (2) - Francesca Tozzi (3) - Stefano Guglielmo (3) - Nicola Origlia (3) - Claudia Verderio (2)

University of Milan Bicocca, School of Medicine and Surgery, Monza, Italy (1) - National Research Council, Institute of Neuroscience, Vedano al Lambro, Italy (2) - National Research Council, Institute of Neuroscience, Pisa, Italy (3)

Extracellular vesicles (EVs) are lipid-encased nanoparticles that convey bioactive signals from a donor to specific target cells, influencing their functions, and can transfer misfolded pathological proteins. Our previous work demonstrated that large (>200 nm) microglial EVs carrying Aβ (Aβ-EVs) are more prone to motility and move faster on neuronal surface (optical manipulation) and can propagate synaptic dysfunction in the mouse brain compared to EVs from non-treated microglia (Ctr-EVs). Here we investigated the effects of Tau on microglia and the interaction of large EVs derived from tau-treated microglia (Tau-EVs) and neurons. Microglial primary culture has been exposed to recombinant tau protein (200nM o/n) and EVs have been isolated from the cell supernatant by differential centrifugation after ATP stimulation. Analyses of EV production and size distribution by Tunable Resistive Pulse Sensing (TRPS) technique didn't highlight any difference between Tau-EVs and Ctr-EVs. Tau-EVs ability to interact and move at the surface of axonal projections has been tested *in vitro* by optical manipulation, finding no differences compared to Ctr-EVs. However, calcium imaging experiments showed that Tau-EVs increase basal calcium levels in neurons, and stereotaxic injection of Tau-EVs but not Ctr-EVs caused LTP impairment at the enthorinal cortex and in its target region, the dentate gyrus of the hippocampus, suggesting the presence of detrimental EV cargoes. We are currently investigating whether the detrimental action of Tau-EVs on neurons and synaptic plasticity depends on tau or other pathogenic molecules. Our preliminary data indicate decreased proliferation (EdU^+ cells $\%_{Ctr}$=26.71%; $\%_{Tau}$=1.99%) and decreased expression of activation markers (CLEC7A, CD11c) in tau-treated microglia, suggesting that tau can drive a microglial senescent phenotype. Further experiments are needed to clarify whether tau-treated microglia may sort ageing/senescent signals into EVs.

ND29 | The interplay between Rab proteins and mitochondrial dysfunction in PD pathology

Martina Brughera [(1)] - Adeena Shafique [(1)] - Francesca Martorella [(1)] - Chiara Frigé [(1)] - Marta Lualdi [(1)] - Tiziana Alberio [(1)]

University of Insubria, Department of Science and High Technology, Busto Arsizio, Italy (1)

Mitochondria undergo processes of fusion, fission and selective degradation via mitophagy, collectively referred as mitochondrial dynamics. Alterations in the fine tuning of these processes negatively impact on neuronal cells, which are characterised by a high energetic demand. In fact, dysfunctional mitochondrial dynamics have been widely associated to neurodegenerative diseases, like Parkinson's disease (PD). Mutations in mitophagy-related *PRKN* gene, which encodes the E3 ubiquitin ligase Parkin, have been linked to autosomal recessive juvenile PD. Recently, also genetic/functional alterations of Rab proteins, involved in vesicles trafficking in the endosomal-lysosomal pathway, have been implicated in PD pathogenesis. In order to investigate the interplay between Rab proteins and dysfunctional mitophagy in the context of PD, we evaluated levels and sub-cellular localization of a subset of Rab proteins in different models: 1) human neuroblastoma SH-SY5Y cells treated with dopamine (DA), to recapitulate the impaired DA homeostasis of early PD stages, 2) SH-SY5Y cells after carbonyl cyanide 3-chlorophenylhydrazone (CCCP) treatment, as a positive control of mitophagy induction, and 3) patient-derived primary skin fibroblasts carrying *PRKN* mutations. After CCCP-induced mitophagy, Rab5 levels were increased, while in DA-induced impaired mitophagy, Rab5 and Rab7 proteins were up-regulated. In *PRKN*-mutated fibroblasts, Rab7 and Rab11 protein levels were increased with respect to controls. Moreover, preliminary data suggest Rabs colocalization with the mitochondrial network. Thus, our results suggest that Rab proteins may be an interesting molecular target to unveil early pathogenetic events related to mitochondria dynamics in PD.

ND30 | Developing a localised GDNF gene therapy to treat neurodegenerative diseases

Lucia Crippa [(1)] - Barbara Bettegazzi [(1)] - Stefano Cattaneo [(1)] - Cristina Porcari [(1)] - Michele Simonato [(2)]

Università Vita Salute San Raffaele, Division of Neuroscience, Milano, Italy (1) - Università degli Studi di Ferrara, Department of Neuroscience and Rehabilitation, Ferrara, Italy (2)

GDNF (glial cell line-derived neurotrophic factor) is a neurotropic factor produced and secreted by glial cells and neurons in the brain, where it has been found to exert potent neuroprotective effects. Based on these data, GDNF has been employed as a drug candidate for the treatment of Parkinson's disease (PD), a progressive neurodegenerative disorder characterized by loss of dopaminergic neurons. However, while promising results were observed in preclinical studies, GDNF-based therapies failed in patients. To fully explore the therapeutic potential of GDNF, we plan to employ integrating lentiviral vectors (LVs) to achieve its prolonged expression in the *substantia nigra (s. nigra)*. At variance with previous studies, we plan to perform the *in vivo* testing in a recently developed genetic animal model of Juvenile PD that resembles most of the features of the human disease. In particular, we will engineer a bicistronic LV containing the GDNF sequence and a reporter gene (GFP) and we will inject it directly in *s. nigra* of mice before the symptoms onset. With the aim to identify an ideal vector design, we performed *in vitro* experiments using the two different isoforms of GDNF (alpha and beta) and investigated possible differences in their expression levels and secretion efficiency. In parallel, to investigate the ability of the LV to reach the target area and express the transgene *in vivo,* we injected wild type animals with a control, GFP-expressing, LV. Confocal microscopy confirmed local transduction of neurons in the *s. nigra* but revealed poor transduction of the dopaminergic population. Stereological counting highlighted potential toxicity of the vector, likely related to the dose employed. Further studies are now ongoing to better characterise this toxicity and to understand whether GDNF production by neurons and/or glial cells in the *s. nigra* might prevent symptoms onset in the mouse model of PD.

ND31 | Analysis of astrocyte calcium activity in alpha7 nicotinic receptor KO Alzheimer's disease mouse model

Alessandro Di Spiezio (1) - Marta Gómez-Gonzalo (1) - Angela Chiavegato (2) - Annamaria Lia (2) - Gabriele Losi (3) - Giorgio Carmignoto (1) - Micaela Zonta (1)

CNR, Institute of Neuroscience, Padua, Italy (1) - University of Padua, Department of Biomedical Sciences, Padua, Italy (2) - CNR, Institute of Nanoscience, Modena, Italy (3)

Alzheimer´s disease (AD) is a neurodegenerative condition which affects more than 40 million people worldwide, as reported by the World Health Organization. AD patients develop cognitive decline and learning issues. Two important hallmarks for AD diagnosis are i) the presence of amyloid plaques, formed by aggregation of amyloid beta (Aβ) peptides and ii) neurofibrillary tangles, characterized by hyper-phosphorylation of microtubule associated protein tau. It is widely believed that accumulation of Aβ peptides triggers the disease arise. Indeed, it was shown that Aβ oligomers are prone to aggregation which induces cell death and inflammation. Furthermore, Aβ oligomers are able to bind the alpha7 nicotinic receptor (A7-nAChR) inducing glutamate release at the pre-synaptic terminal. With the progression of AD disease and the increasing levels of Aβ, the complex A7-nAChR/Aβ gets internalized. This reduces the amount of glutamate released, contributing to a loss of synaptic plasticity. Several studies demonstrate that A7-nAChR is expressed by several cells types in the central nervous system, including astrocytes and microglia. In astrocytes, the activation of A7-nAChR by Aβ oligomers triggers Ca^{2+} elevation and glutamate release. In this project we aim to investigate the role of astrocytic A7-nAChR in the AD pathophysiology. By employing Ca^{2+} sensitive fluorescent probes, such as GCaMP6f, we are characterizing astrocytic spontaneous and evoked Ca^{2+} signal in hippocampal slices from young and aged mice. The same approach will be used in hippocampal slices of A7-nAChR KO AD mouse model to investigate the possible changes of astrocyte Ca^{2+} activity and the influence of astrocytic A7-nAChR on AD progression. Furthermore, we plan to analyse how astrocytic Ca^{2+} events change in presence of pathological levels of Aβ1-42 oligomers, to possibly find a new therapeutic target to treat AD.

ND32 | The UPR response and ER stress in a mouse model of Alzheimer's disease obtained by intracerebroventricular injection of β-amyloid oligomers at different ages

Luca Ghelli [(1)] - Agnese Graziosi [(1)] - Camilla Corrieri [(1)] - Giulia Sita [(1)] - Patrizia Hrelia [(1)] - Fabiana Morroni [(1)]

University of Bologna, Department of Pharmacy and Biotechnology, Bologna, Italy (1)

Alzheimer's disease (AD) is emerging as the most prevalent and socially disruptive illness of aging populations. The major organelle involved in protein folding and quality control, the endoplasmic reticulum (ER), is dramatically affected in AD neurons. The abnormal levels of misfolded proteins at the ER engage the unfolded protein response (UPR) that in turn activates a quick response to restore proteostasis. The present work aims to study the role of UPR an ER stress in AD by using an integrated approach of behavioral test, bioinformatics and biomolecular analysis in a murine model of AD induced. Murine model (mice C57BL76) of AD was obtained by intracerebroventricular injection of β-amyloid oligomers ($A\beta_{1-42}$) at different ages, 3 and 18 months. After 10 days, mice underwent behavioral assessment and then were sacrificed to collect hippocampal sample. RNA sequencing was carried out. The expression of each gene was assessed for different age and treated and not treated mice (3 Aβ1-42/18 Aβ1-42, 3/18 Sham, 18 Sham/18 Aβ1-42, 3 Sham/18 Aβ1-42) by their log2 fold change (log2FC) from the basal-state to investigate the UPR, oxidative stress, inflammation and cell death on hippocampal samples. Our data showed as the impairment induced by Aβ1-42 injection worsen in aging, underlying the involvement of UPR and inflammatory response. RNA-seq data highlighted the presence of 125 common genes between all group, 47 of them involved in important pathways as "Neurodegenerative disorder" and "Inflammation mediated by chemokine and cytokine signaling pathway". The injection of $A\beta_{1-42}$ in mice of different age turn on cellular pathways involved in the cellular response to stress and in the regulation of cellular death. Among them, the involvement of the ER stress and the UPR seem to play a role not only in relation to the presence of Aβ1-42 oligomers but also to the aging process. These results are preliminary and point out the close relation between ER stress,AD and aging process.

ND33 | The role of astrocytic Ca2+ dynamics in Alzheimer’s disease associated neuroinflammation

Neha Kachappilly [(1)] - Emy Basso [(1)] - Paola Pizzo [(1)]

University of Padova, Department of Biomedical Sciences, Padova, Italy (1)

Alzheimer’s Disease (AD), a progressive neurodegenerative disease, is the leading cause of dementia worldwide. Histopathological hallmarks of AD include brain region-specific extracellular deposition of Amyloid-β (Aβ) plaques and intracellular Tau neurofibril aggregation, accompanied by neuronal loss. Current treatments for AD, focussed mainly on addressing Aβ plaques, have had limited success at controlling the cognitive decline associated with the disease. Compared to plaque deposition, neuroinflammation in the brain is known to better correspond with AD-related cognitive decline, and understanding inflammatory mechanisms in AD could reveal potentially more effective therapeutic targets. While microglia are the main initiators of inflammatory responses in the central nervous system, the active role of astrocytes in propagating and sustaining the response has been elaborated in recent years. Astrocytes possess several receptors that respond to extracellular stimuli, including neurotransmitters and inflammatory molecules, causing elevations in cytosolic levels of calcium ion (Ca^{2+}). Intracellular Ca^{2+} responses are an important secondary signal in the physiological function of astrocytes. In this project, we analyse the changes in astrocytic Ca^{2+} handling during Alzheimer’s disease-linked neuroinflammation and the associated functional changes of astrocytes which might promote neurodegeneration. We investigate the role of these Ca^{2+} responses in inducing and amplifying inflammatory responses of astrocytes, including the release of inflammatory cytokines. Further, we investigate the involvement of astrocytic mitochondria in these changes, since they localize to the regions of Ca^{2+} elevation and, by actively taking up or releasing Ca^{2+}, can tune the cytosolic Ca^{2+} signal. We assess whether manipulating mitochondrial Ca^{2+} dynamics can modulate the neuroinflammatory markers of astrocytes in AD.

ND37 | Corroboration of Stathmin-2 in human and murine models of Spinal Muscular Atrophy as potential therapy target

Luca Sali (1) - Lorenzo Quetti (1) - Elisa Pagliari (2) - Michela Taiana (2) - Valentina Melzi (1) - Manuela Garbellini (1) - Giacomo Pietro Comi (1) - Stefania Corti (2) - Monica Nizzardo (1) - Federica Rizzo (2)

Fondazione IRCCS Ca' Granda, Ospedale Maggiore Policlinico, Neurology Unit, Milano, Italy (1) - Università degli studi di Milano, Department of Pathophysiology and Transplantation (DEPT), Milano, Italy (2)

Spinal Muscular Atrophy (SMA), one of the most common infantile inherited neurological diseases characterized by impairing function and survival of lower motor neurons (LMNs), is caused by mutations in the *Survival Motor Neuron 1* gene (*SMN1*). The recently approved SMA therapeutic approaches are focused on increasing the levels of full-length SMN protein. However, some critical issues still remain and research aims to identify novel SMN-independent targets as future complementary strategies, to improve the therapeutic opportunities for all SMA treated patients. To develop this complementary approach, one possibility is to identify downstream genes responsible for selective motoneurons (MNs) dysfunction. Recent studies showed how STATHMIN-2 (STMN2), a protein involved in neurite outgrowth and axonal regeneration, is dysregulated in different neurodegenerative disorders and that its overexpression rescues axonal defects *in vitro* Amyotrophic Lateral Sclerosis (ALS) models. Remarkably, our group also observed STMN2 deregulation in SMA mice spinal cord and in human SMA MNs, suggesting a potential involvement of STMN2 in SMA pathogenesis and hinting at STMN2 as a new therapeutic target. In this study, we investigate for the first time the therapeutic impact of STMN2 modulation on *in vitro* and *in vivo* SMA models. Interestingly, the overexpression of STMN2 ameliorated the typical pathological features in SMA MNs, especially the axonal integrity and complexity. Similarly, in severe SMAΔ7 mouse models the treatment with AAV9 encoding *Stmn2* improved motor phenotype and histologic features related to muscle and neuromuscular junction (NMJ) alteration. Therefore, the capability of STMN2 to ameliorate the effects of reduced SMN levels on axon growth supports the view that axon biology is crucial for SMA pathogenesis and that the modulation of axon stabilizing proteins can modify the disease phenotype. Overall, our data provide evidence that STMN2 may act as a protective modifier of SMA.

ND40 | Emerging value of olfactory neuronal Prokineticin-2 as novel target in Parkinson's disease

Martina Vincenzi (1) - Daniela Maftei (1) - Tommaso Schirinzi (2) - Francesco M. Passali (3) - Piergiorgio Grillo (2) - Henri Zenuni (2) - Davide Mascioli (2) - Riccardo Maurizi (3) - Laura Loccisano (3) - Anna Maria Rinaldi (4) - Massimo Ralli (5) - Stefano Di Girolamo (3) - Alessandro Stefani (2) - Cinzia Severini (6) - Nicola B. Mercuri (2) - Roberta Lattanzi (1)

Sapienza University of Rome, Dept. of Physiology and Pharmacology "V. Erspamer", Rome, Italy (1) - Tor Vergata University of Rome, Unit of neurology, dept. of systems medicine, Rome, Italy (2) - Tor Vergata University of Rome, Unit of ENT, Dept. of clinical sciences and translational medicine, Rome, Italy (3) - Tor Vergata University of Rome, Department of Systems Medicine, Rome, Italy (4) - Sapienza University of Rome, Department of Sense Organs, Rome, Italy (5) - National Research Council of Italy, Dept. of biochemistry and cell biology, Rome, Italy (6)

Prokineticin 2 (PK2) is an inducible chemokine that is overexpressed in response to pathological perturbations to promote neuroprotective or neurotoxic responses. Remarkable neuroprotective effects have been observed in animal models of Parkinson's disease (PD). Specifically, PK2 increases in dopaminergic nigral cells in early stages of neurodegeneration or immediately after toxic exposure, triggering positive bioenergetic and anti-inflammatory cascades. Although a preliminary study measured higher levels of PK2 in the blood and substantia nigra of PD patients, the actual contribution of PK2 to PD in humans remains to be elucidated. Because PK2 signaling is also critical for the proper development and survival of the olfactory system, which is affected early and has the specific neuropathological features of PD, we examined the mRNA and protein expression of PK2 and its receptors (PKRs) in the olfactory neurons (ONs) of 38 PD patients at different stages of disease and 26 healthy controls (CTRLs). PK2 protein expression was also correlated with the expression of different α-synuclein species (total and oligomeric) and with the clinical parameters of the patients. We found that PK2 expression was significantly increased in the ONs of PD patients compared with CTRLs. PK2 expression was higher in early disease stages, proportional to motor severity, and associated with accumulation of pathological α-synuclein forms. Conversely, PKR1 and PKR2 expression levels remained unchanged, suggesting that PK2 increase serves as a mediator and does not compensate for loss of receptors due to neurodegeneration and cell depletion. In later stages of disease, in patients receiving dopaminergic therapy, PK2 expression instead decreased and did not correlate with key clinical features. These data, consistent with preclinical findings, support PK2 as a potential target in the early stages of PD and confirm the reliability of olfactory neurons in reflecting PD pathological changes.

NIM01 | Development of a deep-learning tool for the detection and segmentation of contrast-enhanced lesions in multiple sclerosis patients

Martina Greselin (1) - Po-Jui Lu (1) - Lester Melie-Garcia (1) - Pietro Cerveri (2) - Mario Alberto Ocampo Pineda (1) - Riccardo Galbusera (1) - Alessandro Cagol (1) - Nina Siebenborn (1) - Esther Ruberte (1) - Pascal Benkert (3) - Stefanie Müller (4) - Lutz Achtnichts (5) - Jochen Vehoff (4) - Giulio Disanto (6) - Oliver Findling (5) - Andrew Chan (7) - Anke Salmen (7) - Caroline Pot (8) - Claire Bridel (9) - Chiara Zecca (6) - Tobias Derfuss (10) - Johanna M. Lieb (11) - Luca Remonda (12) - Franca Wagner (13) - Maria I. Vargas (14) - Renaud Du Pasquier (8) - Patrice H. Lalive (9) - Emanuele Pravatà (15) - Johannes Weber (16) - Claudio Gobbi (6) - David Leppert (10) - Ludwig Kappos (1) - Jens Kuhle (10) - Cristina Granziera (1)

Translational Imaging in Neurology (ThINk) Basel, Department of Biomedical Engineering, Faculty of Medicine, University Hospital Basel and University of Basel, Basel, Switzerland (1) - Polytechnic University of Milan, Polytechnic University of Milan, Milan, Italy (2) - Clinical Trial Unit, Department of Clinical Research, University Hospital Basel, University of Basel, Basel, Switzerland (3) - Department of Neurology, Cantonal Hospital St. Gallen, St. Gallen, Switzerland (4) - Department of Neurology, Cantonal Hospital Aarau, Aarau, Switzerland (5) - Neurology Department, Neurocenter of Southern Switzerland, Lugano, Switzerland (6) - Department of Neurology, Inselspital, Bern University Hospital and University of Bern, Bern, Switzerland (7) - Division of Neurology, Department of Clinical Neurosciences, Lausanne University Hospital (CHUV) and University of Lausanne, Lausanne, Switzerland (8) - Department of Clinical Neurosciences, Division of Neurology, Geneva University Hospitals and Faculty of Medicine, Geneva, Switzerland (9) - Research Center for Clinical Neuroimmunology and Neuroscience Basel (RC2NB), University Hospital Basel and University of Basel, Basel, Switzerland (10) - Division of Diagnostic and Interventional Neuroradiology, Clinic for Radiology and Nuclear Medicine, University Hospital Basel, University of Basel, Basel, Switzerland (11) - Department of Radiology, Cantonal Hospital Aarau, Aarau, Switzerland (12) - Department of Diagnostic and Interventional Neuroradiology, Inselspital, Bern University Hospital and University of Bern, Bern, Switzerland (13) - Department of Radiology, Geneva University Hospital and Faculty of Medicine, Geneva, Switzerland (14) - Faculty of biomedical Sciences, Università della Svizzera Italiana, Lugano, Switzerland (15) - Department of Radiology, Cantonal Hospital St. Gallen, St.Gallen, Switzerland (16)

Background: The detection of contrast-enhanced lesions (CELs) is fundamental for the diagnosis of multiple sclerosis (MS) and for the monitoring of MS patients. CELs detection in clinical practice is time-consuming and suffers from high inter- and intra-rater variability, especially for small CELs. **Objective**: To develop a deep-learning tool to automatically detect and segment CELs, which can support clinical radiological practice. **Methods**: We studied 157 MS patients with CELs and 129 patients without CELs who underwent a clinical MRI scan including a T1-weighted image acquired with pre- and post-injection of a gadolinium-based contrast agent, as well as FLAIR images. White matter lesion (WML) masks were obtained with an automated method and then manually corrected. 557 CELs were segmented by experienced clinicians and the masks are utilized as ground truth. For the purpose of this study, we adapted a UNet-based convolutional neural network that had been previously tested for the detection of cortical lesions, which are notoriously small. To overcome the problem of the low frequency of CELs smaller patches are cropped based on WML mask regions. Moreover, a new loss function is introduced, which takes into account the class imbalance and partly also the heterogeneous shape of CELs. Finally, an ablation study was performed to fine-tune the neural network architecture. **Results**: In the test dataset (n= 63 patients, 125 CELs) we obtained a DSC of 0.74, a True Positive (TP) rate of 0.94 and a False Positive (FP) rate of 0.0085. These values for small lesion sizes (3-10 mm^3) are 0.76/0.769/0.090, for medium lesion sizes are 0.67/0.92/0 and for lesions with volume larger than 50 mm^3 are 0.79/1/0. **Conclusions**: Our results were comparable with those obtained in a few previous studies performed using larger datasets, more contrast images and considering larger lesion size. Future work will aim for improving its performance and integration into clinical practice.

NIM02 | Mapping structural disconnection and morphometric similarity in Multiple Sclerosis: a longitudinal study

Mario Tranfa [1] - Alessandra Scaravilli [1] - Maria Petracca [2] - Marcello Moccia [3] - Mario Quarantelli [4] - Sirio Cocozza [1] - Arturo Brunetti [1] - Giuseppe Pontillo [1]

University of Naples, Department of Advanced Biomedical Sciences, Naples, Italy (1) - University of Rome Sapienza, Department of Human Neurosciences, Rome, Italy (2) - University of Naples, Department of Molecular Medicine and Medical Biotechnology, Naples, Italy (3) - Italian National Research Council, Institute of Biostructures and Bioimaging, Naples, Italy (4)

Multiple sclerosis (MS) can be conceptualized as a network disorder. The accumulation of white matter (WM) lesions leads to progressive disconnection, while the development of atrophy disrupts the morphological similarity between brain regions. We used conventional MRI to assess cross-sectional and longitudinal modifications of structural disconnection and morphometric similarity networks in MS.
We retrospectively collected 3T structural brain MRIs of 461 MS patients (age=37.2±10.6y; F:M=324:137), corresponding to 1235 visits (follow-up time=1.9±2.0y; range=0.1-13.3y), and 55 healthy controls (age=42.4±15.7y;F:M=25:30). From 3D-T1w and FLAIR-T2w scans, WM lesions were automatically segmented and the brain was parcellated into 100 cortical (Schaefer atlas) and 14 subcortical (Aseg atlas) regions. For MS patients, subject-level WM masks were registered to MNI space and, using the Lesion Quantification Toolkit, disconnection between pairs of regions was estimated as the proportion of connecting streamlines passing through WM lesions. Likewise, with the Morphometric Inverse Divergence (MIND) method, we computed networks of morphometric similarity between cortical regions from 3D-T1w derived FreeSurfer outputs for both groups. Using network-based statistics, effect of time (and group, for MIND networks) was tested with linear mixed-effects models.
We identified a small subnetwork (27 edges) of significant progressive structural disconnection mainly encompassing cortico-subcortical tracts ($p \leq 0.001$). MIND networks were sensitive to disease status and time, with distributed effects of decreased morphological similarity in large subnetworks of 125 and 174 edges, respectively ($p \leq 0.001$).
We demonstrated that structural disconnection and morphometric similarity networks, as assessed through conventional MRI, are sensitive to MS-related brain damage and its evolution over time, potentially adding to established MRI-derived measures as biomarkers of disease severity and progression.

NIM03 | Investigating Cerebral Lateralization during Visual Stimulation using Functional Transcranial Doppler: A Preliminary Study

Rosita Rabbito (1) - Caterina Guiot (1) - Silvestro Roatta (1)

University of Turin, Neuroscience, Torino, Italy (1)

The Transcranial Doppler (TCD) is a noninvasive neuroimaging technique used to assess the cerebral blood flow velocity (CBFV) in the major cerebral arteries, which closely correlates with cerebral metabolism and function. Compared to other neuroimaging methods, TCD is portable, cost-effective, and robust in assessing brain area activation. Functional Transcranial Doppler (fTCD) employs TCD to analyze brain activity during specific tasks and to detect brain lateralization. Brain activity lateralization refers to the preferential increase in blood flow to one hemisphere compared to the other when performing a particular task or function. This study presents a preliminary exploration of local cerebral perfusion by monitoring bilaterally Posterior Cerebral Arteries (PCA), the suppliers of the visual cortex, using fTCD. The study focuses on selective stimulation of the visual field using a black and white checkerboard pattern with a inversion frequency of 10 Hz. The brain lateralization of nine healthy subjects was evaluated during three visual stimuli: bilateral, left hemifield only and right hemifield only, obtained by extending the checkerboard over the full screen or limiting it to left or right half-screen, respectively. Based on the hemodynamic changes exhibited by the two PCAs a Lateralization Index (LI) was calculated, quantifying the asymmetry (right-left) of the response during each stimulus.
The results demonstrated a low LI during bilateral stimulation. Conversely, a larger and positive (negative) LI was observed in response to the left (right) hemifield stimulations, in agreement with the expected functional splitting of visual pathways at the optic chiasm. The assessment of cerebral lateralization through fTCD presents an additional potential in supporting and monitoring post-stroke rehabilitation, as well as facilitating comprehension of cerebral reorganization subsequent to cerebrovascular accidents, particularly among patients experiencing hemianopia.

NIM04 | Evaluation of prodromal markers of Parkinson's disease in a progressive neurotoxic mouse model using multi-tracer PET-CT imaging

Margherita Tassan Mazzocco (1) - Sara Belloli (2) - Annalisa Pinna (3) - Marcello Serra (4) - Micaela Morelli (4) - Rosa Maria Moresco (1)

Medicine and Surgery Department, University of Milano-Bicocca, Monza, Italy (1) - Institute of Molecular Bioimaging and Physiology (IBFM), CNR, Milan, Italy (2) - Institute of Neuroscience (IN), CNR, Cagliari, Italy (3) - Department of Biomedical Sciences, University of Cagliari, Cagliari, Italy (4)

Parkinson's disease (PD) is a neurodegenerative disorder characterized by progressive loss of dopaminergic neurons affecting the nigrostriatal system. Diagnosis of PD relies on the clinical manifestation of motor symptoms, when neurodegeneration is already advanced, thus compromising the efficacy of disease-modifying treatment approaches. Therefore, research has been focusing on the study of prodromal PD, the stage preceding the appearance of motor signs. Here, we characterized the prodromal stage using a mouse model of PD obtained by sub-chronic treatment with the neurotoxin MPTP and the clearance inhibitor probenecid (MPTPp), by combining in-vivo PET-CT imaging and immunohistochemistry.

A group of 10 mice were injected with 100 mg/kg of probenecid followed by 25 mg/kg of MPTP, twice a week, for a total of 5 weeks. They were monitored longitudinally with PET-CT imaging before treatment and after 1, 3 and 10 MPTPp injections using two radiotracers: [^{18}F]-FP-CIT, a marker of dopamine transporter (DAT) and [^{18}F]-FDG, to assess brain glucose metabolism and metabolic connectivity. They were then sacrificed and brains were collected for post-mortem immunohistochemical analysis of DAT and tyrosine hydroxylase.

We found that both striatal DAT binding in-vivo assessed with [^{18}F]-FP-CIT PET and the density of striatal DAT-positive fibers observed post-mortem started to decrease significantly from the third MPTPp injection. [^{18}F]-FDG uptake was significantly decreased in the striatum and thalamus already at the first administration, while at 10 MPTPp injections [^{18}F]-FDG uptake was increased in the somatosensory and somatomotor cortex. Metabolic connectivity analysis revealed that already after 1 MPTPp injection all significant connections between cortical and subcortical regions were lost, while almost all connections were lost after 10 injections.

Our results suggest that glucose metabolism is an earlier marker than DAT-binding in detecting neurodegeneration in PD mice model.

NIM05 | RUBIK: a fluorescent reporter for combinatorial Cre and Flp recombination

Giada Pessina [(1)] - Mattia Camera [(2)] - Fabrizio Loiacono [(3)] - Antonio Uccelli [(1)] - Francesco Trovato [(4)] - Sebastian Sulis Sato [(1)]

IRCCS Ospedale Policlinico San Martino, Experimental Neuroscience, Genoa, Italy (1) - University of Genoa, Department of Neuroscience, Rehabilitation, Ophthalmology, Genetics, Maternal and Child Health (DINOGMI), Genoa, Italy (2) - IRCCS Ospedale Policlinico San Martino, Immunology, Genoa, Italy (3) - Lund University, Stem Cell Center, Lund, Sweden (4)

Current technologies for precise genetic manipulation of neurons mostly rely on the use of site-specific recombinases (SSR) and CRISPR-Cas9 gene editing tool. Together, these systems are routinely used to generate conditional transgenic models for studying gene functions within specific tissue and/or at specific time points, both *in vivo* and *in vitro*. In this context, the use of drug-inducible SSR is a widely diffused approach to obtain fine spatiotemporal control of specific gene expression. Here we present RUBIK, a fluorescent tool specifically tailored for the reporting of the combinatorial action of Cre and Flp recombinases depending on the spatiotemporal sequence of recombination events. To test this reporter, we generated and isolated a stable knock-in HeLa cell line using the CRISPR-Cas9 technology and single cell-sorting. Our preliminary data show proper functioning of our system upon recombination with both Cre and Flp recombinases alone or in combination. Additionally, we optimized a variant of the Trimethoprim (TMP)-inducible Flp recombinase (FlpDD) designed to decrease the activity of the recombinase without TMP. Overall, we were able to set up a system that could be exploited in the field of neuroscience by the generation of knock-in mice expressing RUBIK. These transgenic mice could be resourceful to study and precisely define the roles of specific neuronal populations depending on the activity of Cre and Flp.

NIM06 | Gradient of Dentate-Thalamo-Cortical Tract Microstructural Disruption: Applying Diffusion MRI Profilometry in Friedreich Ataxia

Mario Tranfa (1) - Sara Bosticardo (2) - Matteo Battocchio (2) - Louise Corben (3) - Martin Delatycki (3) - Gary Egan (4) - Nellie Georgiou-Karistianis (4) - Serena Monti (5) - Giuseppe Palma (6) - Chiara Pane (7) - Francesco Saccà (7) - Simona Schiavi (2) - Louisa Selvadurai (4) - Alessandro Daducci (2) - Arturo Brunetti (1) - Ian H. Harding (8) - Sirio Cocozza (1)

University of Naples, Department of Advanced Biomedical Sciences, Naples, Italy (1) - University of Verona, Diffusion Imaging and Connectivity Estimation (DICE) Lab, Verona, Italy (2) - Murdoch Children's Research Institute, Bruce Lefroy Centre for Genetic Health Research, Parkville, Australia (3) - Monash University, School of Psychological Sciences, Clayton, Australia (4) - National Research Council, Institute of Biostructures and Bioimaging, Naples, Italy (5) - National Research Council, Institute of Nanotechnology, Naples, Italy (6) - Università di Napoli, Department of Neurosciences Reproductive and Odontostomatological Sciences, Naples, Italy (7) - Monash University, Monash Biomedical Imaging, Clayton, Australia (8)

The dentate-thalamo-cortical tract (DTT) is the main cerebellar efferent pathway. Microstructural changes of the DTT are considered core features of Friedreich ataxia (FRDA). Nonetheless, whether some areas of the DTT are more impacted than others, possibly determining a gradient of disruption, is still a matter a debate. This study aimed to investigate microstructural integrity along the DTT in FRDA using a profilometry diffusion MRI (dMRI) approach.
MRI data from 45 FRDA patients (mean age: 33.2±13.2, M/F:26/19) and 37 healthy controls (HCs; mean age: 36.5±12.7, M/F:18/19) were included in this cross-sectional multicenter study. A profilometry analysis was performed on dMRI data by first using tractography to define the DTT as the white matter tract connecting the dentate nuclei to the contralateral motor cortex. The tract was then divided into 100 segments and diffusion tensor metrics of microstructural integrity (fractional anisotropy [FA], mean diffusivity [MD], and radial diffusivity [RD]) were extracted at each segment and compared between FRDA and HC groups. The process was replicated on the arcuate fasciculus for comparison.
The profilometry analysis indicated that, across all diffusion metrics, the region of the DTT connecting the dentate nucleus and thalamus was more impacted in FRDA than downstream cerebral sections from the thalamus to the cortex. The arcuate fasciculus was minimally impacted.
Our study further expands the current knowledge about brain involvement in FRDA, showing that the presence of microstructural abnormalities within the main cerebellar-cerebral tract are weighted to early segments of the tract (i.e., the superior cerebellar peduncle). These findings are in line with the hypothesis of the DTT undergoing anterograde degeneration arising from the dentate nuclei and progressing to the primary motor cortex.

NI01 | Investigation of neuronal Ca2+ hyperexcitability and neuro-inflammatory state in an Alzheimer's disease mouse model

Martina Bedetta [(1)] - Nikita Arnst [(1)] - Mariagrazia Mancuso [(1)] - Annamaria Lia [(1)] - Nelly Redolfi [(1)] - Elisa Greotti [(2)] - Paola Pizzo [(1)]

University of Padova, Department of Biomedical Sciences, Padova, Italy (1) - Italian National Research Council, Neuroscience Institute, Padova, Italy (2)

Alzheimer's disease (AD), the most common cause of dementia, is an irreversible neurodegenerative disease characterized by progressive memory loss and cognitive deficits. Despite intensive investigations, pathogenic mechanisms of AD are poorly known and effective treatments are still lacking. It is reasonable that some important neurophysiological processes are altered years before the onset of clinical symptoms, highlighting the importance of identifying early dysfunctions and biomarkers useful for both therapeutic and diagnostic purposes. Even though most AD cases are sporadic, a small percentage is due to autosomal dominant mutations in amyloid precursor protein (APP), presenilin-1 (PS1), and presenilin-2 (PS2) genes. We used an AD mouse model (B6.152H), double transgenic for PS2 (N141I) and APP (Swedish mutations), which is characterized by neuronal electrical hyperexcitability. In these mice, changes in brain circuitry, relevant to the development of AD, are detectable at both 3 and 6 months of age, earlier than the onset of spatial memory deficits, revealed at 8 months. We first analysed the state of excitatory and inhibitory synapses in the somatosensory cortex (SSCx) of AD and WT mice at 2, 6 and 9 months. Moreover, we evaluated Ca^{2+} activity in neurons from *ex vivo* and *in vivo* SSCx preparations by using advanced imaging techniques, to investigate spontaneous and evoked Ca^{2+} hyperexcitability at different disease stages. Moreover, since glial cells are actively involved in synaptic transmission, and microglia are emerging as critical negative regulators of neuronal Ca^{2+} hyperactivity in mice, we plan to assess and modulate microglia inflammatory state in order to possibly rescue neuronal Ca^{2+} phenotype in AD mice.

NI02 | Is Circulating AgRP Neuropeptide a Novel Mediator of Neuroimmune Communication in Multiple Sclerosis?

Eleonora Cornacchia [(1)] - Alice Laroni [(1,2)] - Caterina Bason [(1)] - Fabrizio Loiacono [(1)] - Tiziana Altosole [(1)] - Matilde Inglese [(1,2)] - Antonio Uccelli [(1,2)] - Tiziana Vigo [(1)]

IRCCS Ospedale Policlinico San Martino, Genoa (1) - University of Genoa (2)

The interplay between nervous and immune systems is emerging as an important mechanism of immune cell function control. Immune cells express the receptors for neurotransmitters and neuropeptides released by neurons of both peripheral and central nervous system. We have previously demonstrated that activation of hypothalamic neurons producing Agouti-related peptide (AgRP) impairs hematopoiesis and promotes the generation of Treg by reducing the release of norepinephrine in bone marrow and thymus, thus suggesting a possible immune-regulatory function of these neurons. AgRP neurons are activated in the mouse model of Multiple Sclerosis (MS), the experimental autoimmune encephalomyelitis. Activation of AgRP neurons leads to increased expression of AgRP, that is partially released into circulation, as these neurons lye out of the blood brain barrier. Accordingly, we have observed an increase of AgRP level in the serum of people with MS, suggesting that AgRP neurons are activated also in MS. Whether increase of circulating AgRP is specific of MS or also occurs in other neurological diseases of the central nervous system is completely unknown. AgRP is a potent competitive antagonist of the melanocortin receptors 3 and 4 (MCR), and previous studies indicated that immune cells express MC3R at mRNA level. However, a comprehensive expression map of MC3R receptor in immune cells at protein level is still missing, and a possible effect exerted by AgRP on immune cell functions has never been investigated.

Here we have quantified the levels of AgRP peptide in the serum of people with Parkinson disease (PD) and with ischemic stroke (IS), to understand whether increased level of AgRP is a common hallmark or neuronal damage or if it is specific of MS. Then, we have performed an extensive FACS analysis to assess the expression of MC3R in mouse and human immune cells. Our results reveal an increase of circulating AgRP in pwPD and 7 day after IS, when neuronal death is prominent, suggesting that AgRP may be a marker of neurodegeneration. Moreover, we have demonstrated that the AgRP receptor MC3R is expressed by human and mouse dendritic cells (DC) and NK cells. Functional in vitro assays suggest that AgRP may exert a regulatory effect on these cells.

These preliminary results appear promising both in suggesting AgRP as a marker of neuronal damage in neurological diseases and in describing a new mechanism of neuro-immune interplay mediated by AgRP and its receptor MC3R.

NI04 | Unveiling the role of exosomal miRNAs in the spreading of neuroinflammation

Francesca De Chirico (1) - Francesca Massenzio (1) - Eleonora Poeta (1) - Giorgia Babini (1) - Sabrina Petralla (1) - Manon Elise Libotte (1) - Giampaolo Zuccheri (1) - Barbara Monti (1)

University of Bologna, Department of Pharmacy and Biotechnology, Bologna, Italy (1)

Neuroinflammation is a crucial pathogenic mechanism that commonly underlies most neurodegenerative diseases. Microglia, the immune cells of the brain, play a critical role according to the stage of neuropathology. Indeed, at early phases of brain diseases microglia display the neuroprotective, alternatively activated phenotype which is switched to the classically activated pro-inflammatory subtype at later stages, when activated microglia contribute to neurodegeneration. The microglial phenotypic shift is characterized by a change in the release of bioactive molecules (especially proteins and miRNAs), both soluble and through extracellular vesicles. With the progression of neuropathology, microgliosis, i.e. an increase in number and reactivity of microglial cells, is always observed, thus suggesting a spreading of microglia activation. However, the role of extracellular vesicles released by activated microglia in neuroinflammation spreading has never been demonstrated. We took advantage of the in vitro model of murine microglia N9 cells to evaluate the effect of conditioned media or isolated vesicles to spread the neuroinflammation through the release of specific miRNAs. We demonstrated that conditioned media or exosomes obtained from pharmacologically activated microglia were able to promote a pro-inflammatory phenotype to control cells, leading us to prove, in vitro, the existence of a neuroinflammation spreading process mediated by the secretome of microglia with a crucial role of extracellular vesicles in terms of miRNAs content.
In this regard, the downregulation of the inflamma-miR-34a, by using cleaving sequences of anti-miR34a DNAzyme delivered by DNA nanostructures, led to a reduction of microglia polarization towards the neurotoxic phenotype confirming the involvement of miR-34a in the neuroinflammatory process.

NI08 | Elucidate the role of microglia in fmr1ko mice model

Antonella Borreca [(1)] - Michela Matteoli [(1)]

CNR, Institute of Neuroscience, Milan, Italy (1)

Fragile X Syndrome (FXS) is the most common neurodevelopmental disorder associated with intellectual disabilities. In FXS, an increased CGG triplet repetition (more than 200) in the *fmr1* gene, cause the hyper-methylation of the 5'UTR and, in turn leads to the gene silencing and the absence of the Fragile X mental retardation protein (FMRP). FMRP is an RNA binding protein, able to regulate the expression of different RNAs, and fundamental for synapses formation and function. FMRP is expressed in neurons but also in astrocytes and microglia, the other key cells of the brain. Despite many studies have focused their attentions on the role of FMRP in neurons and locally at synapses, only a few pieces of evidences have reported the role of the protein in other brain cells types. In preliminary results, performed in *fmr1*ko mice, revealed an hyperactivated state of microglia, measured with phagocytic marker CD68, and an increase of microglia-specific transcriptor factor PU.1/Spi1. Interestingly, activated microglia is present in many neurodegenerative disorders, such as Alzheimer's disease (AD), Huntington's disease, and Amyotrophic Lateral Sclerosis (ALS). Based on this rationale, we aim to clarify the role of FMRP in microglia through a deep transcriptional profiling of *fmr1*ko microglial cells compared to wild type (WT). Furthermore, increase level of transcriptional factor PU.1 in *fmr1ko* mice suggest an unbalance of epigenetic mechanism in absence of FMRP and a Chip-Seq analysis to study a PU.1-regulated genes in *fmr1ko* microglia will be fundamental. This approach will be instrumental to identify new molecular pathways responsible for the activated-state of microglia in *fmr1*ko mice and suggest that modulating PU.1 expression may be a valid therapeutic target to prevent microglial-mediated hyperactivation in *fmr1ko* mice model.

NI11 | Alginate displays anti-inflammatory properties in the ibotenic-lesioned rat brain

Federica Raimondi (1) - Stefania Bartoletti (1) - Elisa Ren (1) - Beatrice Casadei Garofani (1) - Arianna Capodiferro (1) - Giulia Della Rosa (2) - Gemma Palazzolo (2) - Giulia Curia (1)

University of Modena and Reggio Emilia, Department of Biomedical, Metabolic, and Neural Sciences, Modena, Italy (1) - Istituto Italiano di Tecnologia, Department of Neuroscience and Brain Technologies, Genova, Italy (2)

Neuroinflammation involves different populations of brain-resident or peripheral immune cells, which may be part of innate or adaptive immunity. The integrity of the blood-brain barrier is crucial to ensure the normal inflammatory framework of the brain, and its damage leads to increased recall of periphery immune cells. This condition is known to be related to several neurological and neurodegenerative diseases. Alginate is a marine biopolymer capable of gelation and is used for drug delivery, wound healing, and tissue engineering. While its structural, pro-differentiation, and neuroprotective properties have been shown in vitro, the in vivo anti-inflammatory effects have not been deeply described. The main purpose of this study is to highlight the anti-inflammatory and protective properties of alginate in the brain lesioned with ibotenic acid (IBO), an excitotoxic agent. Sprague-Dawley adult male rats were bilaterally injected in ventral *Cornus Ammonis 3* with IBO. Four days later, a subgroup of these rats received a further injection of saline or alginate. After ten weeks, neuronal damage and the presence of resident and infiltrating immune cells were evaluated by immunofluorescence using immune response biomarkers. Behavioral impairments in the different groups were also investigated. Our experiments revealed that alginate hydrogel is still present and filling the lesion ten weeks after the injection, suggesting that it is neither dissolved nor washed out by the liquor. Moreover, we found a low immune response *in vivo*, which makes it a promising candidate to support cellular differentiation and neuronal maturation in cell-based therapy. Our results shed light on the future application of alginate in innovative therapeutic interventions for neurological diseases aiming at repairing brain lesions.

NI12 | Neuroprotective effect of butyrate in Friedreich's Ataxia models

Francesca Sciarretta (1) - Veronica Ceci (2) - Katia Aquilano (2) - Daniele Lettieri Barbato (2)

IRCCS, Fondazione Santa Lucia, Rome, Italy (1) - University of Rome Tor Vergata, Biology Deparment, Rome, Italy (2)

Friedreich's Ataxia (FA) is an inherited autosomal neurodegenerative disorder caused by a mutation in the gene encoding for mitochondrial protein frataxin (FXN) leading to the decrease of FXN content. Neurological diseases are usually associated with microbiota dysfunction. Analysing the microbiota of FXN knock-in/knock-out (KIKO) mice, we found that KIKO mice show a decrease of bacteria producing the short chain fatty acid butyrate. This is a small, short chain fatty acid physiologically produced by gut microbiota and has been studied for its neuroprotective and anti-inflammatory role. Our preliminary data show that the cerebellum of KIKO mice has signs of inflammation and an increase of microglial population. We generated an *in vitro* model of FA consisting in a microglial cell line (BV2) with FXN downregulation (FXN-). We observed that FXN- cells are more susceptible to a pro-inflammatory stimulus (i.e., lipopolysaccharides, LPS) if compared to cells with normal FXN levels and that butyrate treatment can counteract the increase of pro-inflammatory cytokine production. By supplementing butyrate through diet for 16 weeks, we observed that KIKO mice underwent an amelioration of neuro-mobility as assessed by rotarod test. Overall, these data suggest the use of butyrate as a safe and valuable molecule to counteract neuroinflammation in FA.

NI13 | BTK inhibitors modulate microglial extracellular vesicles release to regulate remyelination in Multiple Sclerosis

Matteo Tartaglia (1) - Mariassunta De Luca (2) - Cristina Limatola (2) - Myriam Catalano (2)

Sapienza University of Rome, Department of Human Neurosciences, Rome, Italy (1) - Sapienza University of Rome, Department of Physiology and Pharmacology, Rome, Italy (2)

It is a common finding that myelin repairing mechanisms in people with MS are altered and ineffective. As myelin offers protection against stressors, demyelinated axons are exposed to damaging factors, leading to neurodegeneration commonly found especially in the progressive MS phenotypes. To date, remyelination is one of the greatest unmet needs in MS. Since its underlying mechanisms are still unclear, their deeper comprehension may help in understanding the disease modifying treatments (DMT) effects on myelin repairing mechanisms and offers food for thought for new therapeutic approach. Extracellular vesicles (EVs) represent a way of intercellular communication thanks to their contain of proteins, nucleic acids and more on. EVs derived from human mesenchymal stem cells and from neural stem cells have already shown the potential therapeutic ability to ameliorate both progressive and relapsing remitting models of MS. Bruton's tyrosine kinase inhibitors (BTKi) are a new DMT class primary acting on myeloid cells as microglia, the immune cells of the central nervous system. A recent study has shown that ibrutinib, one BTKi, is able to modulate EVs release from BV2 cells, a microglial murine cell line. Our first aim is to analyze EVs released by microglia (untreated, proinflammatory, anti- inflammatory and BTKi-treated cells) in single culture and in a complex system (brain cortex and spinal cord cultures), both in de- and a re-myelinating *in vitro* models. Second aim is to evaluate the role played by EVs released from microglial cells in a remyelination *in vitro* model of MS. First aim will provide further insights about microglial response to BTKi, mainly acting on myeloid cells and currently tested on MS patients. Second aim will clarify microglial role, exerted through EVs, in remyelination.

NI15 | A comprehensive molecular imaging study in a mouse model of CMT1B neuropathy

Mattia Camera (1) - Federico Zaottini (2) - Roberta Resaz (3) - Riccardo Picasso (2) - Marina Grandis (1) - Simonetta Astigiano (3) - Laura Emionite (3) - Simona Pigozzi (4) - Vanessa Cossu (5) - Gianmario Sambuceti (5) - Cecilia Camera (5) - Mehrnaz Hamedani (1) - Angelo Schenone (1) - Carlo Martinoli (2) - Lucilla Nobbio (1)

University of Genoa, Italy, DEPARTMENT OF NEUROLOGY, REHABILITATION, OPHTHALMOLOGY, GENETICS, MATERNAL AND CHILD HEALTH (DINOGMI, Genova, Italy (1) - IRCCS Ospedale Policlinico San Martino, Genoa, Italy, Radiology, Genova, Italy (2) - IRCCS Ospedale Policlinico San Martino, Genoa, Italy, Animal Facility, Genova, Italy (3) - IRCCS Ospedale Policlinico San Martino, Genoa, Italy, DISC, Genova, Italy (4) - IRCCS Ospedale Policlinico San Martino, Genoa, Italy, Medicina Nucleare, Genova, Italy (5)

We recently generated a mouse model carrying the D61N heterozygous mutation in the MPZ gene, which encodes a structural protein of peripheral myelin. This mutation causes, in humans, a severe early-onset form of CMT1B, characterized by extensive demyelination. Considering that previous studies described a potential role of innate immunity in the pathogenesis of neuropathy in CMT animal models, and molecular imaging has proved to be a valuable non-invasive means to explore the development of micro-inflammatory processes *in vivo*, we aimed at verifying the presence of immune/inflammatory cell infiltration in the nerves and muscles of this novel CMT1B model, and correlating the imaging findings with disease progression. To do this, we subjected MPZ-D61N heterozygous ($MPZ^{D61N/+}$) and homozygous ($MPZ^{D61N/D61N}$) mice to a sequential protocol including evaluation of motor performance, ultrasonography, magnetic resonance imaging (MRI) before and after administration of Ferumoxytol (a contrast agent classically used to label macrophages), and whole-body micro-PET imaging, after administration of a tracer for the TPSO protein. Finally, animals were sacrificed, and sciatic nerves and muscles were collected for histological analyses. Both MRI and micro-PET imaging indicated that macrophage infiltration is absent in our models. However, histological analysis revealed a marked increase in the number of neutrophils in the sciatic nerve of $MPZ^{D61N/D61N}$ mice. Moreover, our MRI scanning protocol was sensitive enough to detect structural alterations in the sciatic nerves of both $MPZ^{D61N/+}$ and $MPZ^{D61N/D61N}$ mice. Even if this study represents a pilot investigation of inflammatory response in the muscle and nerve in a severe form of CMT1B neuropathy, our results suggest that low-grade inflammation is present in the sciatic nerve of D61N-homozygous mice and may exacerbate the neuropathic phenotype.

NI16 | Butyrate decreases the regulatory function of human natural killer cells and promotes their maturation

Federico Carlini [(1)] - Margherita Squillario [(2)] - Matteo Capaia [(1)] - Valeria Lusi [(1)] - Davide Baganra [(3)] - Serena Palmeri [(4)] - Federico Ivaldi [(5)] - Michele Piana [(6)] - Alice Laroni [(1)]

IRCCS Ospedale Policlinico San Martino, U.O. Neuroscienze Sperimentali, Genoa, Italy (1) - IRCCS Ospedale Policlinico San Martino, LISCOMP Lab, Genoa, Italy (2) - University of Genoa, Department of Experimental Medicine, Genoa, Italy (3) - IRCCS Istituto Giannina Gaslini, Dinogmi, University of Genova, Genoa, Italy (4) - University of Genoa, Department of Internal Medicine and Medical Specialties, Genoa, Italy (5) - University of Genoa, Department of Mathematics, Genoa, Italy (6)

An increased amount of evidence suggest that bacteria-derived metabolites play a role in shaping immune cell function. We and others have shown dysfunction in regulation of the T-cell response by NK cells in multiple sclerosis (MS). The objective of this work was to assess whether microbial-derived metabolites affect the regulatory function of NK cells. We cultured NK cells from healthy controls (HC) in presence/absence of the butyrate and of different tryptophan derivatives in presence of activating cytokines and then co-cultured them with autologous T cells. We cultured PBMCs from HC in presence/absence of the butyrate and tryptophan derivatives and assessed the phenotype of NK cells through a 14-marker flow cytometry panel. We performed Assay for Transposase-Accessible Chromatin using sequencing (ATAC-seq) on NK cells sorted from PBMCs cultured in presence of butyrate. ATAC-Seq data were analyzed by GUAVA tool starting from raw data to the calls of the identified peaks and their functional characterization in Gene Ontology (GO). Further, data were compared to those from a public single-cell RNA sequencing dataset of NK cell clusters. We found that butyrate decreased the percentage of $CD56^{bright}$ NK cells, increased the percentage of $CD69^{+}$ NK cells and decreased the suppressor function of NK cells. Tryptophan derivatives did not affect the function, nor the phenotype of NK cells. ATAC-seq revealed that butyrate affects chromatin modifications in 218 genes causing demethylation in the promoter region of most (> 80%) genes, involved in (i) the regulation of specific populations of T cells within the immune system, (ii) the modulation of cellular processes in neurons and (iii) to inflammation. The epigenetic signature of butyrate-treated NK cells increased similarly to terminally differentiated $CD56^{dim}$ NK cells. In conclusion, we describe a novel effect of butyrate on human NK cells, whereby butyrate induces their maturation and decreases their regulatory function.

NI17 | Transcranial magnetic stimulation restores glial response and microvasculature integrity in experimental Parkinson’s disease

Maria De Carluccio (1) - Giuseppina Natale (1) - Federica Servillo (1) - Paolo Calabresi (2) - Maria Teresa Viscomi (3) - Veronica Ghiglieri (4)

Università Cattolica del Sacro Cuore, Neuroscience, Roma, Italia (1) - Policlinico Gemelli, Neurologia, Roma, Italia (2) - Università Cattolica del Sacro Cuore, Department of Life Science and Public Health, Roma, Italia (3) - Università Telematica San Raffaele, Department of Human Sciences and Quality of Life Promotion, Roma, Italia (4)

Transcranial magnetic stimulation (TMS) is a form of non-invasive brain stimulation used to induce neuroplasticity in the brain and a non-pharmacological treatment in different neuropathological conditions. Although TMS has been shown to modulate several aspects of neuronal plasticity, there is still limited information on how TMS works at the cellular and molecular levels, as well as its impact on non-neuronal cells, like astrocytes and microglia and other different components of the Blood-Brain-Barrier (BBB), the pericytes and the endothelial cells (EC).This study investigated changes in RNA and protein levels of the main components of the BBB following different treatments of theta-burst stimulation (TBS), repetitive TMS patterns, such as continuous (cTBS) and intermittent (iTBS) protocols, used in a 6-hydroxydopamine (6-OHDA)-lesioned hemiparkinsonian rat model. In the striatum of parkinsonian animals, mRNA and protein levels of markers of pericytes, such as PDGFβ, decreased significantly after the lesion compared to control animals. Notably, in 6-OHDA-lesioned animals, the decrease of pericytes markers correlated with a reduction of PECAM-1 (also known as CD31), a marker of vessel endothelial cells, and these changes were associated with impairments in brain microvasculature. After the injury, treatment with iTBS protected both pericytes and endothelial cells of the striatal microvasculature from 6-OHDA-induced damage. Similarly, cTBS treatment restores microvessels integrity by restoring lectin and PECAM-1 expression, thus improving microvasculature integrity, that was found similar to what was observed in unlesioned animals. Collectively, these findings demonstrate the ability of TMS to modulate specific aspects of non-neuronal cell phenotype and restore the microvasculature integrity, highly impaired after 6-OHDA lesion, potentially contributing to the known effects of TMS on neuroprotection.

NI18 | NLRP3-inflammasome inhibition by Leishmania-derived factors in the neuropathogenesis of Alzheimer's Disease (AD): assessing the molecular and therapeutic role

Francesca La Rosa [(1)] - ilaria Varotto [(2)] - Marina Saresella [(3)] - Claudio Bandi [(2)] - Mario Clerici [(1)]

IRCCS Fondazione Don Carlo Gnocchi-Onlus, Laboratorio di biologia molecolare e traslazionale, Milan, Italy (1) - University of Milan, Dipartimento di Bioscienze, Milan, Italy (2) - IRCCS Fondazione Don Carlo Gnocchi-Onlus, Laboratorio di biologia molecolare e traslazionale, Milano, Italy (3)

The hygiene hypothesis suggests that certain aspects of modern life are linked to lower rates of exposure to pathogens and lower immune system stimulation, being in turn positively related to Alzheimer's-disease risk. Amazonian tribes exhibit an exceptionally high prevalence of infections in individuals of all ages. It results in a better-regulated degree of inflammation and improved cognitive functions after infection with Leishmanias (L) parasite. Indeed, parasitic infections recognized the same receptors that sense misfolded-Aß in microglia; it is followed by subsequent regulation of the NLRP3, a cytosolic multiprotein complex mainly expressed in myeloid cells and composed of the Nod-like receptor (NLR), the adaptor apoptosis-associated speck-like protein (ASC), and the pro-caspase1, leading to the cleavage in bioactive IL1ß and IL18.
Since the N is considered a therapeutic target against neuroinflammation in AD, herein we investigated a possible connection between L *tarantulae (t)*, nonpathogenic for humans to reduce IL1ß, IL18, Caspase1 release and ASC-speck formation. THP1cells wild-type and ASC-knockout were cultured in unstimulated or Lypolisaccaride+Aß-treatment post *Lt-p10* infection. L phagocytosis were quantified by confocal mycroscopy, cytokines by ELLA and inflammasome formation and by AMNIS FlowSight. ASC-speck formation as well as IL18 and caspase1 were significant ($p<0.05$)downregulated in LPS+Aß THP1 wild-type and post L-infection compare to unstimulated cells; no significant differences were found for IL1ß and NLRP3 proteins between wild-type and ASC-knockout cells.
L is able to modulate the inflammasome-assembly suppressing the inflammatory response. Given this ability, we propose a protective role of L in Alzheimer's disease as a consequence of the molecular inhibition of the NLRP3-inflammasome.

NI09 | dCK intracellular localization is regulated by serine 11 phosphorylation and predicts the response to cladribine treatment in T cells

Federico Carlini (1) - Stella Maglio (1) - Marco Ponassi (2) - Erika Iervasi (2) - Gabriela Coronel Vargas (2) - Elena Cerutti (1) - Aldo Profumo (2) - Camillo Rosano (2) - Alice Laroni (1) - Paola Barboro (2) - Antonio Uccelli (1)

IRCCS Ospedale Policlinico San Martino, U.O. Neuroscienze Sperimentali, Genova, Italy (1) - IRCCS Ospedale Policlinico San Martino, U.O.S. Proteomica e Spettrometria di Massa, Genova, Italy (2)

Activation of cladribine (2CdA), a drug approved for multiple sclerosis, is driven by a high deoxycytidine kinase (dCK)/5'nucleotidase (5NT) ratio in the cytoplasm. Due to their high dCK/5NT ratio, lymphocytes are a preferential target for 2CdA. We have previously demonstrated that 2CdA-induced apoptosis in stimulated T cells is correlated with enhanced dCK expression and activity and that cladribine treatment of lymphocytes affects the phosphorylation of dCK in activated T cells. Little is known regarding how phosphorylation of dCK at different sites affects its activity and intracellular localization.
The objective of this work was to assess the composition of differently phosphorylated dCK isoforms in healthy donor peripheral blood mononuclear cells and T cells.
For this aim, protein lysates from CD4+ T cells treated with or without anti-CD3/CD28 dynabeads and cultured for 72h, were digested with trypsin after protein purification. Phosphopeptides were enriched using High-SelectTM Fe-NTA enrichment kit and analyzed using Q Exactive™ Plus Hybrid Quadrupole-Orbitrap™ mass spectrometer. Activated/not activated CD4+ T cells were mounted on microscope slides and, after staining with dCK, CD4+ and SYTO™ 16, confocal images were acquired with a Leica Stellaris confocal microscope to observe dCK nuclear Vs cytoplasmic localization.
Activation of T cells led to phosphorylation of dCK at Ser11 residue. In Ser11-P-enriched, activated CD4+ T cells, dCK was localized mostly in the cytoplasm, whereas in unstimulated cells the highest dCK signal density was in the nucleus. In conclusion, these results suggest that T cell activation leads to phosphorylation of dCK at Ser11 leading to dCK preferential localization in the cytoplasm. Assessing baseline phosphorylation of Ser11 in T cells from untreated patients with MS may permit to identify those who are more likely to respond to treatment.

NI20 | Microglia-released extracellular vesicles counteract age-related cognitive impairment and restore microglia homeostasis in the aging brain of male and female mice

Arianna Rinaldi (1) - Myriam Catalano (1) - Cristina Limatola (1)

University of Rome "La Sapienza", Dept. of Physiology and Pharmacology, Rome, Italy (1)

Aging is a time-related deterioration of physiological functions, associated with oxidative stress, chronic inflammation and high production of inflammatory compounds. During brain aging, among the first changes, there are modifications of microglia (MG), the resident immune cells of the central nervous system (CNS), which become hyper-responsive and nonfunctional. These cells undergo the most prominent aging-related changes in both the morphological and functional phenotypes; their progressive loss of neuroprotective functions results in chronic neuroinflammation (i.e., increase of inflammatory markers and dystrophic morphology), which impacts the whole brain homeostasis. All these alterations occur differently in aged males and females and cause cognitive functions deterioration, lack of motor coordination and memory loss. Extracellular vesicles (EVs) are key players of the inter-cellular communication between donor and target cells; EVs are exploited by the cells to exchange a package of molecular information consisting of lipids, proteins and nucleic acids. Given their properties, EVs are emerging as a promising tool to develop revolutionary non-invasive therapies for a wide range of diseases. We investigated the effect of EVs released by anti-inflammatory MG and intranasally administered to both male and female mice during the old age (16-18 months). We evaluated *in vivo* memory and anxiety-like behavior and *ex vivo* inflammatory and homeostatic MG profile by RT-qPCR and cellular morphology analysis. EVs treatment ameliorated brain functionality in terms of reduction of anxiety; we observed a reduction of pro-inflammatory genes (*Il-6, Tnf*α, *Il1*β, *Cd86*) and a recovery of the homeostatic MG morphology. The effects on microglia were dissimilar in male and female mice pointing out sex-differences in the anti-aging effects of EVs treatment. The findings indicate EVs as an innovative strategy to slow down the effects of aging on brain functioning.

NP01 | Cardiac functional and structural abnormalities in a mouse model of CDKL5 Deficiency Disorder

Giulia Candini [1] - Manuela Loi [1] - Stefano Bastianini [1] - Giorgio Medici [1] - Laura Gennaccaro [1] - Nicola Mottolese [1] - Marianna Tassinari [1] - Beatrice Uguagliati [1] - Giovanna Zoccoli [1] - Stefania Trazzi [1] - Christian Bergamini [2] - Elisabetta Ciani [1]

University of Bologna, Department of Biomedical and Neuromotor sciences, Bologna, Italy (1) - University of Bologna, Department of Pharmacy and Biotechnology, Bologna, Italy (2)

CDKL5 (cyclin-dependent kinase-like 5) deficiency disorder (CDD) is a rare and severe neurodevelopmental disease that mostly affects girls who are heterozygous for mutations in the X-linked CDKL5 gene. The lack of CDKL5 protein expression or function leads to the appearance of numerous clinical features, including early-onset seizures, marked hypotonia, autistic features, and severe neurodevelopmental impairment. Mouse models of CDD, *Cdkl5* KO mice, exhibit several behavioral phenotypes that mimic CDD features, such as impaired learning and memory, social interaction, and motor coordination. CDD symptomatology, along with the high CDKL5 expression levels in the brain, underscores the critical role that CDKL5 plays in proper brain development and function. Nevertheless, the improvement of the clinical overview of CDD in the past few years has defined a more detailed phenotypic spectrum; this includes very common alterations in peripheral organ and tissue function, such as gastrointestinal problems, irregular breathing, hypotonia, and scoliosis, suggesting that CDKL5 deficiency compromises not only CNS function but also that of other organs/tissues. Here we report, for the first time, that a mouse model of CDD, the heterozygous *Cdkl5* KO (*Cdkl5* +/-) female mouse, exhibits cardiac functional and structural abnormalities. The mice also showed QTc prolongation and increased heart rate. These changes correlate with a marked decrease in parasympathetic activity to the heart and in the expression of the Scn5a and Hcn4 voltage-gated channels. Moreover, the *Cdkl5* +/- heart shows typical signs of heart aging, including increased fibrosis, mitochondrial dysfunctions, and increased ROS production. Overall, our study not only contributes to the understanding of the role of CDKL5 in heart structure/function but also documents a novel preclinical phenotype for future therapeutic investigation.

NP02 | Epigenetic effects of exposure to the endocrine disruptor ethinyl estradiol in differentiated SH-SY5Y cells

Camilla Corrieri [1] - Agnese Graziosi [1] - Giulia Sita [1] - Luca Ghelli [1] - Patrizia Hrelia [1] - Fabiana Morroni [1]

Alma Mater Studiorum-University of Bologna, Pharmacology, Bologna, Italy (1)

Endocrine disruptors (EDs) are a heterogeneous group of chemicals that may interfere with several mechanisms of the endocrine system. Some of them derived from natural sources, but most are synthetic chemicals released by human activity into the environment, such as ethinyl estradiol (EE), an estrogen medication. Their danger lies in the constant exposure of the global population and in the possibility of an induction of toxic effects not only on the hormones, but also on various physiological systems, such as the central nervous system. Among the mechanisms by which EDs could impact human health, of interest is the modulation of microRNA (miRNA). Therefore, the aim of the study was to identify the cellular pathways deregulated following exposure to subtoxic concentrations of EE and the effects on the expression of miRNAs implicated in pathways involved in neurotoxicity mechanisms. A human neuroblastoma cell line, SH-SY5Y, was exposed to different concentrations of EE for 48h to identify the experimental conditions to which cytotoxicity was not induced and which did not affect the cellular redox state. Afterwards, a miRNA profiling unveiled an important modulation in the expression of some miRNAs implicated in neurotoxicity. The genes target of these miRNAs were identified thanks to a computational analysis and the results showed that several of them were members of the PI3K/Akt/mTOR pathway. The modulation of the genes and the proteins included in this pathway has been validated by Real-Time PCR and Western Blotting assays. The study identified upregulation in the phosphorylation of Akt and mTOR, while p53 underwent downregulation following exposure to EE. Although these analyses are preliminary, they allow to investigate the role of EE as ED able to modulate pathways involved in neurodegeneration and tumor development.

Funded by Project PON. Also, thank to Project PRIN 2017 (Prot. 2017MLC3NF) and Fondazione del Monte di Bologna e Ravenna (Prot. 1355bis/2021)

NP05 | Altered neuronal morphology and synaptic protein synthesis in brain cortex of mouse model for Angelman Syndrome: rescuing effect of serotonin receptor 7 stimulation

Amelia Pizzella [1] - Eduardo Penna [2] - Yan Liu [2] - Natalia Abate [1] - Mario De Gregorio [1] - Enza Lacivita [3] - Marcello Leopoldo [3] - Rossella Di Giaimo [1] - Carla Perrone-Capano [4] - Xiaoning Bi [2] - Michel Baudry [2] - Marianna Crispino [1]

University of Naples, Department of Biology, Naples, Italy (1) - Western University of Health Sciences, College of Osteopathic Medicine of the Pacific, Pomona, United States (2) - University of Bari Aldo Moro, Department of Pharmacy-Drug Sciences, Bari, Italy (3) - University of Naples, Department of Pharmacy, Naples, Italy (4)

The serotonin receptor 7 (5-HT7R) is a G-protein coupled receptor, which is involved in various forms of synaptic plasticity in the brain. This receptor has been associated with several neurological and neurodevelopmental disorders including schizophrenia, depression and Autism Spectrum Disorders (ASD), which are characterized by abnormal neuronal connectivity and intellectual disabilities. Angelman Syndrome (AS) is a rare neurodevelopmental disorder exhibiting a high comorbidity with ASD. Interestingly, our research has revealed that several signalling pathways affected in AS are positively regulated by 5-HT7R. We first demonstrated that 5-HT7R activation by acute systemic injection of LP-211, a potent and selective agonist, could rescue behavioral impairment (fear conditioning test) in AS mice, suggesting a potential involvement of 5-HT7Rs in AS pathogenesis. We next investigated synaptic protein synthesis using synaptosomes isolated from the cortex of AS and wild type (WT) mice. Our results showed a significant impairment in synaptosomal protein synthesis in AS as compared to WT mice and stimulation of synaptosomes with LP-211 rescued this impairment. Additionally, we examined neuronal morphology of hippocampal primary neurons from AS and WT mice brains. Our results revealed a decrease in the density of dendritic spines in neurons from AS mice as compared to WT. Interestingly, chronic stimulation for 3 days with LP-211 restored spine density to the value found in neurons from WT mice. Overall, our study demonstrates that activation of 5-HT7R can rescue multiple synaptic plasticity mechanisms that are disrupted in AS mice. These results thus provide a new perspective for therapeutical approaches of the disease, using 5-HT7R receptor as a potential target.

NP06 | Generation of induced pluripotent stem cells lines to study the cognitive deficits associated with Noonan Syndrome

Giulia Sbrini [(1)] - Zaira Tomasoni [(1)] - Raffaele Badolato [(2)] - Cristina Missale [(1)] - Chiara Fiorentini [(1)] - Federica Bono [(1)]

University of Brescia, Department of Molecular and Translational Medicine, Brescia, Italy (1) - Azienda Socio Sanitaria Spedali Civili di Brescia, Pediatrics clinics, Brescia, Italy (2)

Noonan syndrome (NS) is a rare, genetically inherited disease due to mutations on the PTPN11 gene, leading to the hyperactivity of the encoded Shp-2 tyrosine-kinase. Around 50% of NS patients show cognitive disabilities, such as learning and memory impairments. Unfortunately, drugs for the treatment of these symptoms are not available and how Shp-2 hyperactivation affects neuronal functions is still elusive. The comprehension of the molecular mechanisms underlying NS-associated cognitive deficits is needed for understanding their pathogenesis and for the identification of new molecular targets. Hence, we used the induced pluripotent stem cells (iPSCs) technology as an “in vitro” tool to model NS and study the molecular mechanisms underlying the cognitive deficits. Peripheral blood mononuclear cells were isolated from three different NS patients with cognitive deficits and the 188A>G mutation in the PTPN11 gene and treated with the Yamanaka factors (Oct3/4, c-Myc, Klf4, Sox2). We then selected two iPSCs lines for each patient and we confirmed their stemness and their pluripotency both by gene expression and immunofluorescence analyses. Moreover, iPSCs-derived embryoid bodies were analyzed for the expression of typical ectodermic endodermic and mesodermic genes, and therefore for their ability to generate all the body tissues. Each iPSCs line was also karyotyped and analyzed for the presence of the 188A>G mutation in the PTPN11 gene. Since it has been shown how Shp-2 hyperactivity impacts glutamatergic neuron function, we also developed a protocol to differentiate iPSCs into neuronal cells with a high percent of glutamatergic neurons.
Thus, iPSCs lines generated from NS-patients and cognitive deficits, and their corresponding genetic-manipulated lines with the corrected mutation used as control, represent a powerful tool to identify the molecular mechanisms underlying the neurological feature of the disease.

NP08 | Sub-toxic glyphosate treatment alters GABAergic synapses in murine hippocampal neurons

Giuseppe Chiantia (1) - Debora Comai (1) - Vita Cardinale (1) - Antonia Gurgone (1) - Andrea Marcantoni (2) - Maurizio Giustetto (1)

University of Turin, Department of Neuroscience "Rita Levi-Montalcini", Turin, Italy (1) - University of Turin, Department of Drug Science, Turin, Italy (2)

Glyphosate (Gly)-based herbicides (GBH), that are widely used worldwide, act by inhibiting the enolpyruvylshikimate-3-phosphate synthase of the shikimate pathway, an enzyme expressed in plants but not in mammals. We recently found that chronic *exposure* to toxic doses of GBH produces: reduced expression of neurotransmitters, increased cellular oxidative stress, augmented anxiety/depression-like behaviors, and impairments of learning and memory. Although the safety of Gly is actively investigated, so far very little is known on the mechanisms underlying Gly neurotoxicity. Importantly, only few studies addressed the neuronal consequences of the acceptable daily intake (ADI) dose. To fill this gap, we investigated the effects of an acute Gly (3µM) treatment on both structural and functional organization of synaptic connections by patch-clamp recordings, immunofluorescence and confocal microscopy of primary hippocampal neuronal cultures. The measure of evoked excitatory and inhibitory postsynaptic currents showed that Gly administration reduces the amplitude of inhibitory GABAergic neurotransmission without affecting glutamatergic responses. Moreover, both the amplitude and frequency of miniature inhibitory postsynaptic currentswere affected by Gly administration, suggesting that GABAergic transmission is affected both pre- and postsynaptically. In line with this, we found that Gly reduces the number of postsynaptic $GABA_A$ receptors channels, the size of the readily releasable pool of synaptic vesicles and the number of release sites. Consistently, morphological analyses showed that the density of both pre- (vGAT-positive) and post-synaptic (gephyrin-positive) inhibitory compartments are reduced in Gly-treated neurons while excitatory contacts are unaffected. In sum, these data disclose novel neuronal and synaptic abnormalities caused by the ADI dose of Gly, which strongly prompts for further investigations to assess the underlying molecular pathways.

NP09 | Exposure to interleukin 15 modulates hippocampal synaptic transmission and interfere with episodic memory formation via serotonin receptor activation

Maria Amalia Di Castro (*1) - Stefano Garofalo (*1) - Erika Di Pietro (1) - Alessandro Mormino (1) - Cristina Limatola (2,3)

Department of Physiology and Pharmacology, Sapienza University of Rome, Italy (1) - IRCCS Neuromed, Pozzilli, Italy (2) - Department of Physiology and Pharmacology, Sapienza University, Laboratory affiliated to Istituto Pasteur, Italy (3)

Cytokines are potent immunomodulators exerting several pleiotropic effects also in the CNS. They influence neuronal functions and circuit activity with effects on memory processes and behaviours. In particular, the interleukin 15 (IL-15) play a pivotal role in the differentiation, activation and viability of innate and adaptive lymphocytes. Here, we unravel the neuromodulatory function of the IL-15 in the brain. In *ex vivo* hippocampal slices, acute exposure to IL-15 enhances GABA release and reduces glutamatergic currents, while chronic delivery of IL-15 in vivo into the hippocampus alters synaptic transmission and impairs memory in the novel object recognition test. Moreover, we describe the involvement of serotonin receptor in the neuromodulatory effect of IL-15: indeed the pre-treatment with a selective 5HT3A receptor antagonist prevents the IL-15-mediated effects on inhibition and ameliorates performance in NOR. These findings provide new insights into the complex interaction between cytokines and CNS at the functional levels with implications on behaviour.

NP10 | 5-HT7R Stimulation Modulates Synaptic Protein Synthesis in Autism Spectrum Disorders

Kardelen Dalım Filiz [1] - Natalia Abate [2] - Amelia Pizzella [2] - Mario De Gregorio [2] - Floriana Volpicelli [1] - Marianna Crispino [2] - Carla Perrone-Capano [1]

University of Naples Federico II, Department of Pharmacy, Naples, Italy (1) - University of Naples Federico II, Department of Biology, Naples, Italy (2)

Autism Spectrum Disorders (ASD) is a complex neurodevelopmental disorder characterized by persistent deficits in social interaction and restricted patterns of behavior. The etiology of ASD remains poorly understood, but emerging evidence suggests a potential involvement of the serotonin system. Serotonin signaling, mediated by the serotonin receptor 7 (5-HT7R), plays a crucial role in neurodevelopment, synaptic plasticity, and social behavior. Our research focused on local synthesis at central nervous system synapses using synaptosomes, an *in vitro* model mimicking synaptic contacts in living organisms. We used a novel, non-radioactive protein labeling technique named SUnSET (Surface and Sensing of Translation) to investigate the synaptic system of protein synthesis. We examined the effects of a selective 5-HT7R agonist named LP-211 on protein synthesis of the synaptosomal fraction from the brain cortex of BTBR mice, a well-established animal model of ASD. We observed that LP-211 treatment significantly increased synaptic protein synthesis. These results suggest that the activation of 5-HT7R by LP-211, in an ASD animal model, promotes the molecular processes involved in protein synthesis at the synapses. To further investigate the specificity of this effect, synaptosomes were co-incubated with LP-211 and the selective 5-HT7R antagonist SB-269970. This treatment successfully restored protein synthesis levels to the baseline, indicating a 5-HT7R modulatory role in synaptic protein synthesis. These results shed light on the involvement of 5-HT7R in the molecular mechanisms underlying synaptic plasticity in ASD, opening a new perspective in the identification of therapeutic targets for ASD.

NP11 | Natural Killer cells modulate sleep pressure via Interferon-gamma

Alessandro Mormino [1] - Federico Tucci [1] - Claudio Babiloni [1] - Germana Cocozza [1] - Cristina Limatola [1] - Stefano Garofalo [1]

Sapienza, university of Rome, Department of Physiology and Pharmacology, Rome, Italy (1)

Sleep is a fundamental physiological process regulated by several mechanisms. In the last years it has been demonstrated that not only neurons, but also glial cells and immune system cells participate in in the sleep process through the release of cytokines, regulating neuronal activity.
In this study, we investigate the involvement of Natural Killer (NK) cells in the regulation of the sleep process. Through a combination of EEG analysis and behavioral assessments, we demonstrate that NK cells exert a significant influence on sleep process. In particular, NK cells depletion in mice induces a decrease in the sleeping time during the resting phase and a reduction in the sleep pressure, a mark of the necessity to sleep, during the active-to-resting phase transition. We, also, identify the interferon (IFN)-γ as key molecular mediator responsible for this regulation. Using XMG1.2 antibody, which specifically targets and blocks IFN-γ, we demonstrated that the blockade of the cytokine mimics the effect of NK cell-depletion in mice. Moreover, we demonstrated that IFN-γ and NK cells modulate the activation of $nNOS^{+}$ (neuronal nitric oxide synthase) inhibitory interneurons, that are directly responsible in the generation of the sleep pressure. These findings provide important insights into the complex network of factors involved in the regulation of sleep, and highlights the intricate interplay between the immune system and neuronal circuits in physiological processes.

NP12 | Voluntary running ameliorates brain development and behavioral performance in a mouse model of CDKL5 deficiency disorder

Beatrice Uguagliati (1) - Nicola Mottolese (1) - Camilla Bruna Cerchier (1) - Marianna Tassinari (1) - Manuela Loi (1) - Giulia Candini (1) - Giorgio Medici (1) - Stefania Trazzi (1) - Elisabetta Ciani (1)

Bologna University, Biomedical and Neuromotor Sciences, Bologna, Italy (1)

Cyclin-dependent kinase-like 5 (CDKL5) deficiency disorder (CDD) is a rare neurodevelopmental disease caused by mutations in the X-linked CDKL5 gene. CDD is characterized by a broad spectrum of clinical manifestations, including early-onset refractory epileptic seizures, intellectual disability, hypotonia, visual disturbances, and autism-like features. The Cdkl5 knockout (KO) mouse recapitulates several features of CDD, including autistic-like behavior, impaired learning and memory, and motor stereotypies. These behavioral alterations are accompanied by diminished neuronal maturation and survival, reduced dendritic branching and spine maturation, and marked microglia activation. There is currently no cure or effective treatment to ameliorate the symptoms of the disease. Aerobic exercise is known to exert multiple beneficial effects in the brain, not only by increasing neurogenesis, but also by improving motor and cognitive tasks. To date, no studies have analyzed the effect of physical exercise on the phenotype of a CDD mouse model. In view of the positive effects of voluntary running on the brain of mouse models of various human neurodevelopmental disorders, we sought to determine whether voluntary daily running, sustained over a month could improve brain development and behavioral defects in *Cdkl5* KO mice. Our study showed that long-term voluntary running improved hyperlocomotion and impulsivity behaviors, and memory performance of *Cdkl5* -/Y mice. This is correlated with increased hippocampal neurogenesis, neuronal survival, spine maturation, and inhibition of microglia activation. These behavioral and structural improvements were associated with increased BDNF levels. Given the positive effects of BDNF on brain development and function, the present findings support the positive benefits of exercise as an adjuvant therapy for CDD.

NP17 | Investigating Cerebellar Abnormalities in a mouse model of lysosomal lipid storage disease: Implication for Social Behavior

Greta Massa (1) - Serena Camuso (1) - Jessica Tiberi (1) - Roberta Stefanelli (2) - Piergiorgio La Rosa (2) - Maria Teresa Fiorenza (2) - Sonia Canterini (2)

Ph.D Program in Behavioral Neuroscience, Sapienza University of Rome, Department of Psychology, Division of Neuroscience, Rome, Italy (1) - Sapienza University of Rome, Department of Psychology, Division of Neuroscience, Rome, Italy (2)

The cerebellum is a versatile brain region that regulates various motor/non-motor behaviors. Thus, impairments in its architecture and circuitry lead to a wide range of neurodevelopmental/neuropsychiatric disorders. During postnatal development, the cerebellum undergoes changes in its cellular arrangement, guided by the Brain-derived Neurotrophic Factor (BDNF), which plays a role in appropriate development, synaptogenesis, and maintenance of cerebellar connectivity. In Niemann-Pick C1 disease (NPC1), a rare lysosomal lipid storage disease, we have previously shown that a decline in Sonic hedgehog (Shh) and BDNF expression in the first weeks of postnatal development disrupts cerebellar granule cell (GC) migration and maturation, influencing the final cerebellar cytoarchitecture. In *Npc1* mice, through immunohistochemistry/ Neurolucida analysis at various stages of early postnatal life, we observed a significant decrease in the amount, size and tortuosity of glomeruli, the main synaptic contact between GC dendrites and axons of mossy fibers. These results prompted us to investigate the presence of functional abnormalities in mature glutamatergic synapses. Therefore, by subcellular protein fractionation, we examined the expression levels of specific presynaptic (Syntaxin 1A, VAMP2, SNAP-25) and postsynaptic (Drebrin, Shank3) proteins during different stages of postnatal development, finding a general SNAP-25 deficiency in *Npc1* mice compared to *wild type* (wt) mice. Furthermore, through Golgi-Cox staining analysis, we characterized the density and morphology of GC dendritic spines in the internal granular layer, both in *wt* and mutant mice, to identify abnormalities in synapse maturation and pruning processes, during critical stages of cerebellar development. Finally, *Npc1* male mice showed no preference for social/nonsocial cues in a typical task used to study autistic-like behavior, consistent with studies indicating reduced levels of cerebellar BDNF in autistic patients.

NP19 | Dissecting the mechanism of action and neuroplastic potential of molecules targeting NMDA or 5-HT receptors

Sonia Sonda [(1)] - Marco Banzato [(2)] - Alberto Furlan [(2)] - Sara De Martin [(2)] - Andrea Mattarei [(2)] - Stefano Comai [(2)] - Marco Pappagallo [(3)] - Paolo L Manfredi [(3)] - Diana Pendin [(4)]

University of Padova, Department of Biomedical Sciences, Padova, Italy (1) - University of Padova, Department of Pharmaceutical and Pharmacological Sciences, Padova, Italy (2) - Relmada Therapeutics, Inc., Coral Gables, FL, United States (3) - National Research Council, Neuroscience Institute, Padova, Italy (4)

Major depressive disorder (MDD) is a debilitating illness characterized by depressed mood, anhedonia, and impaired cognitive function. In the last few years, compounds targeting serotonin receptors 5-HT_{2A} or N-methyl-D-aspartate (NMDA) receptors have emerged as promising rapid-onset antidepressant agents. These drugs, collectively known as fast-acting antidepressant, may show clinical efficacy within 24 hours after the first administration and their effects may last long after the compounds have been cleared from the body. Fast-acting antidepressants have been shown to promote structural and functional neuroplasticity in the prefrontal cortex (PFC), which is one of the brain areas that have been documented to undergo neuronal atrophy in MDD patients. Despite evidence for clinical efficacy is growing, the molecular mechanism underlying the neuroplastic effect of these drugs is not fully understood. Intriguingly, neuroplasticity-promoting molecules seem to share similar downstream pathways that recapitulate in the increase of dendritogenesis and spinogenesis; nonetheless, their upstream binding partners and interactors might be distinct. Our aim is to investigate the mechanism of action of fast-acting antidepressants targeting the serotonin receptor 5-HT_{2A} or the NMDA receptor, such as psilocin and esmethadone, respectively. By combining pharmacological, genetic, and imaging interventions on primary cortical neurons and neuron-differentiated cells, we aspire at dissecting the intracellular pathways underlying the neuroplastic potential of fast-acting antidepressants. Our approach may be useful to characterize the neuroplastic activity of new 5-HT_{2A} agonists or NMDA receptor antagonists.
